# Supplementary material for: Predictions of Milk Fatty Acid Contents by Mid-Infrared Spectroscopy in Chinese Holstein Cows
Source: Molecules. 2023 Jan 9;28(2):666. doi: 10.3390/molecules28020666 (PMC9864415; doi:10.3390/molecules28020666)
Supplement: Supplementary file 1 [file molecules-28-00666-s001.zip › Supplementary File S1.pdf]

| Fatty acid | Pre-processing algorithm | MIRS range ($cm^{-1}$) | Model | Basis (g/100g) | $R^2$ (Training set) | RPD (Training set) | $R^2$ (Test set) | RPD (Test set) |
|---|---|---|---|---|---|---|---|---|
| C10.0 | MSC | 3017~2823/1805~1734 | LassoR | Fat | 0.404 | 1.296 | 0.429 | 1.320 |
| C10.0 | DER1 | 3017~2823/1805~1734 | LassoR | Fat | 0.494 | 1.403 | 0.436 | 1.328 |
| C10.0 | DER2 | 3017~2823/1805~1734 | LassoR | Fat | 0.508 | 1.426 | 0.351 | 1.243 |
| C10.0 | SNV | 3017~2823/1805~1734 | LassoR | Fat | 0.421 | 1.315 | 0.433 | 1.327 |
| C10.0 | SG | 3017~2823/1805~1734 | LassoR | Fat | 0.382 | 1.271 | 0.423 | 1.299 |
| C10.0 | MSC | 3017~2823/1805~1734 | RidgeR | Fat | 0.281 | 1.176 | 0.366 | 1.227 |
| C10.0 | DER1 | 3017~2823/1805~1734 | RidgeR | Fat | 0.308 | 1.199 | 0.387 | 1.258 |
| C10.0 | DER2 | 3017~2823/1805~1734 | RidgeR | Fat | 0.391 | 1.273 | 0.432 | 1.309 |
| C10.0 | SNV | 3017~2823/1805~1734 | RidgeR | Fat | 0.296 | 1.189 | 0.319 | 1.203 |
| C10.0 | SG | 3017~2823/1805~1734 | RidgeR | Fat | 0.208 | 1.122 | 0.210 | 1.119 |
| C10.0 | MSC | 3017~2823/1805~1734 | RFR | Fat | 0.182 | 1.104 | 0.185 | 1.110 |
| C10.0 | DER1 | 3017~2823/1805~1734 | RFR | Fat | 0.151 | 1.080 | 0.134 | 1.080 |
| C10.0 | DER2 | 3017~2823/1805~1734 | RFR | Fat | 0.154 | 1.088 | 0.230 | 1.133 |
| C10.0 | SNV | 3017~2823/1805~1734 | RFR | Fat | 0.179 | 1.098 | 0.150 | 1.082 |
| C10.0 | SG | 3017~2823/1805~1734 | RFR | Fat | 0.104 | 1.047 | 0.073 | 1.020 |
| C10.0 | MSC | 3017~2823/1805~1734 | PLSR | Fat | 0.269 | 1.177 | 0.407 | 1.306 |
| C10.0 | DER1 | 3017~2823/1805~1734 | PLSR | Fat | 0.196 | 1.126 | 0.410 | 1.310 |
| C10.0 | DER2 | 3017~2823/1805~1734 | PLSR | Fat | 0.211 | 1.138 | 0.308 | 1.209 |
| C10.0 | SNV | 3017~2823/1805~1734 | PLSR | Fat | 0.291 | 1.195 | 0.425 | 1.328 |
| C10.0 | SG | 3017~2823/1805~1734 | PLSR | Fat | 0.243 | 1.159 | 0.386 | 1.284 |
| C10.0 | MSC | 3017~2823/1805~1734 | LassoR | Milk | 0.765 | 2.068 | 0.724 | 1.836 |
| C10.0 | DER1 | 3017~2823/1805~1734 | LassoR | Milk | 0.785 | 2.160 | 0.684 | 1.653 |
| C10.0 | DER2 | 3017~2823/1805~1734 | LassoR | Milk | 0.776 | 2.114 | 0.685 | 1.697 |
| C10.0 | SNV | 3017~2823/1805~1734 | LassoR | Milk | 0.753 | 2.017 | 0.742 | 1.939 |
| C10.0 | SG | 3017~2823/1805~1734 | LassoR | Milk | 0.741 | 1.970 | 0.642 | 1.590 |
| C10.0 | MSC | 3017~2823/1805~1734 | PLSR | Milk | 0.717 | 1.894 | 0.673 | 1.758 |
| C10.0 | DER1 | 3017~2823/1805~1734 | PLSR | Milk | 0.704 | 1.852 | 0.610 | 1.612 |
| C10.0 | DER2 | 3017~2823/1805~1734 | PLSR | Milk | 0.692 | 1.820 | 0.639 | 1.675 |
| C10.0 | SNV | 3017~2823/1805~1734 | PLSR | Milk | 0.709 | 1.872 | 0.697 | 1.828 |
| C10.0 | SG | 3017~2823/1805~1734 | PLSR | Milk | 0.715 | 1.884 | 0.595 | 1.581 |
| C10.0 | MSC | 3017~2823/1805~1734 | RFR | Milk | 0.588 | 1.556 | 0.720 | 1.895 |
| C10.0 | DER1 | 3017~2823/1805~1734 | RFR | Milk | 0.610 | 1.603 | 0.770 | 2.066 |
| C10.0 | DER2 | 3017~2823/1805~1734 | RFR | Milk | 0.624 | 1.634 | 0.744 | 1.894 |
| C10.0 | SNV | 3017~2823/1805~1734 | RFR | Milk | 0.592 | 1.562 | 0.712 | 1.851 |
| C10.0 | SG | 3017~2823/1805~1734 | RFR | Milk | 0.558 | 1.503 | 0.731 | 1.876 |
| C10.0 | MSC | 3017~2823/1805~1734 | RidgeR | Milk | 0.689 | 1.793 | 0.713 | 1.863 |
| C10.0 | DER1 | 3017~2823/1805~1734 | RidgeR | Milk | 0.715 | 1.875 | 0.625 | 1.563 |
| C10.0 | DER2 | 3017~2823/1805~1734 | RidgeR | Milk | 0.738 | 1.954 | 0.645 | 1.600 |
| C10.0 | SNV | 3017~2823/1805~1734 | RidgeR | Milk | 0.683 | 1.779 | 0.715 | 1.869 |
| C10.0 | SG | 3017~2823/1805~1734 | RidgeR | Milk | 0.643 | 1.676 | 0.591 | 1.517 |
| C10.0 | MSC | 4000~400 | RidgeR | Fat | 0.400 | 1.231 | 0.286 | 1.151 |
| C10.0 | DER1 | 4000~400 | RidgeR | Fat | 0.522 | 1.365 | 0.324 | 1.185 |
| C10.0 | DER2 | 4000~400 | RidgeR | Fat | 0.682 | 1.538 | 0.291 | 1.162 |
| C10.0 | SNV | 4000~400 | RidgeR | Fat | 0.394 | 1.243 | 0.253 | 1.144 |
| C10.0 | SG | 4000~400 | RidgeR | Fat | 0.345 | 1.203 | 0.252 | 1.130 |
| C10.0 | MSC | 4000~400 | RFR | Fat | 0.148 | 1.085 | 0.158 | 1.092 |
| C10.0 | DER1 | 4000~400 | RFR | Fat | 0.201 | 1.121 | 0.337 | 1.192 |

| | | | | | | | | |
|---|---|---|---|---|---|---|---|---|
| C10.0 | DER2 | 4000~400 | RFR | Fat | 0.182 | 1.106 | 0.206 | 1.112 |
| C10.0 | SNV | 4000~400 | RFR | Fat | 0.146 | 1.082 | 0.188 | 1.111 |
| C10.0 | SG | 4000~400 | RFR | Fat | 0.133 | 1.074 | 0.186 | 1.105 |
| C10.0 | MSC | 4000~400 | PLSR | Fat | 0.172 | 1.111 | 0.353 | 1.251 |
| C10.0 | DER1 | 4000~400 | PLSR | Fat | 0.209 | 1.153 | 0.338 | 1.237 |
| C10.0 | DER2 | 4000~400 | PLSR | Fat | 0.181 | 1.128 | 0.327 | 1.226 |
| C10.0 | SNV | 4000~400 | PLSR | Fat | 0.182 | 1.111 | 0.179 | 1.111 |
| C10.0 | SG | 4000~400 | PLSR | Fat | 0.125 | 1.080 | 0.201 | 1.126 |
| C10.0 | MSC | 4000~400 | LassoR | Fat | 0.864 | 2.646 | 0.205 | 0.974 |
| C10.0 | DER1 | 4000~400 | LassoR | Fat | 0.940 | 3.749 | 0.165 | 0.976 |
| C10.0 | DER2 | 4000~400 | LassoR | Fat | 0.991 | 8.437 | 0.125 | 0.949 |
| C10.0 | SNV | 4000~400 | LassoR | Fat | 0.870 | 2.712 | 0.199 | 0.959 |
| C10.0 | SG | 4000~400 | LassoR | Fat | 0.750 | 1.957 | 0.228 | 1.064 |
| C10.0 | MSC | 4000~400 | LassoR | Milk | 0.931 | 3.779 | 0.671 | 1.685 |
| C10.0 | DER1 | 4000~400 | LassoR | Milk | 0.973 | 5.851 | 0.691 | 1.692 |
| C10.0 | DER2 | 4000~400 | LassoR | Milk | 0.998 | 19.886 | 0.695 | 1.720 |
| C10.0 | SNV | 4000~400 | LassoR | Milk | 0.927 | 3.676 | 0.693 | 1.760 |
| C10.0 | SG | 4000~400 | LassoR | Milk | 0.875 | 2.820 | 0.676 | 1.717 |
| C10.0 | MSC | 4000~400 | PLSR | Milk | 0.655 | 1.728 | 0.713 | 1.879 |
| C10.0 | DER1 | 4000~400 | PLSR | Milk | 0.635 | 1.665 | 0.700 | 1.838 |
| C10.0 | DER2 | 4000~400 | PLSR | Milk | 0.654 | 1.763 | 0.670 | 1.752 |
| C10.0 | SNV | 4000~400 | PLSR | Milk | 0.654 | 1.735 | 0.718 | 1.896 |
| C10.0 | SG | 4000~400 | PLSR | Milk | 0.620 | 1.639 | 0.671 | 1.754 |
| C10.0 | MSC | 4000~400 | RFR | Milk | 0.598 | 1.579 | 0.707 | 1.790 |
| C10.0 | DER1 | 4000~400 | RFR | Milk | 0.645 | 1.680 | 0.763 | 2.017 |
| C10.0 | DER2 | 4000~400 | RFR | Milk | 0.650 | 1.693 | 0.730 | 1.858 |
| C10.0 | SNV | 4000~400 | RFR | Milk | 0.610 | 1.602 | 0.697 | 1.785 |
| C10.0 | SG | 4000~400 | RFR | Milk | 0.609 | 1.603 | 0.737 | 1.897 |
| C10.0 | MSC | 4000~400 | RidgeR | Milk | 0.673 | 1.735 | 0.660 | 1.681 |
| C10.0 | DER1 | 4000~400 | RidgeR | Milk | 0.723 | 1.888 | 0.679 | 1.729 |
| C10.0 | DER2 | 4000~400 | RidgeR | Milk | 0.776 | 2.060 | 0.684 | 1.735 |
| C10.0 | SNV | 4000~400 | RidgeR | Milk | 0.688 | 1.772 | 0.686 | 1.748 |
| C10.0 | SG | 4000~400 | RidgeR | Milk | 0.663 | 1.720 | 0.613 | 1.564 |
| C11.0 | MSC | 3017~2823/1805~1734 | LassoR | Fat | 0.433 | 1.330 | 0.520 | 1.421 |
| C11.0 | DER1 | 3017~2823/1805~1734 | LassoR | Fat | 0.514 | 1.434 | 0.547 | 1.478 |
| C11.0 | DER2 | 3017~2823/1805~1734 | LassoR | Fat | 0.451 | 1.349 | 0.520 | 1.428 |
| C11.0 | SNV | 3017~2823/1805~1734 | LassoR | Fat | 0.431 | 1.327 | 0.526 | 1.429 |
| C11.0 | SG | 3017~2823/1805~1734 | LassoR | Fat | 0.446 | 1.343 | 0.532 | 1.449 |
| C11.0 | MSC | 3017~2823/1805~1734 | RidgeR | Fat | 0.328 | 1.222 | 0.502 | 1.383 |
| C11.0 | DER1 | 3017~2823/1805~1734 | RidgeR | Fat | 0.367 | 1.257 | 0.494 | 1.388 |
| C11.0 | DER2 | 3017~2823/1805~1734 | RidgeR | Fat | 0.410 | 1.301 | 0.525 | 1.433 |
| C11.0 | SNV | 3017~2823/1805~1734 | RidgeR | Fat | 0.325 | 1.219 | 0.509 | 1.390 |
| C11.0 | SG | 3017~2823/1805~1734 | RidgeR | Fat | 0.294 | 1.192 | 0.428 | 1.289 |
| C11.0 | MSC | 3017~2823/1805~1734 | RFR | Fat | 0.185 | 1.087 | 0.437 | 1.274 |
| C11.0 | DER1 | 3017~2823/1805~1734 | RFR | Fat | 0.204 | 1.114 | 0.387 | 1.202 |
| C11.0 | DER2 | 3017~2823/1805~1734 | RFR | Fat | 0.247 | 1.152 | 0.500 | 1.311 |
| C11.0 | SNV | 3017~2823/1805~1734 | RFR | Fat | 0.173 | 1.080 | 0.441 | 1.280 |
| C11.0 | SG | 3017~2823/1805~1734 | RFR | Fat | 0.192 | 1.104 | 0.295 | 1.155 |
| C11.0 | MSC | 3017~2823/1805~1734 | PLSR | Fat | 0.330 | 1.237 | 0.521 | 1.454 |
| C11.0 | DER1 | 3017~2823/1805~1734 | PLSR | Fat | 0.342 | 1.243 | 0.470 | 1.382 |

| | | | | | | | | |
|---|---|---|---|---|---|---|---|---|
| C11.0 | DER2 | 3017~2823/1805~1734 | PLSR | Fat | 0.299 | 1.210 | 0.410 | 1.311 |
| C11.0 | SNV | 3017~2823/1805~1734 | PLSR | Fat | 0.351 | 1.255 | 0.538 | 1.480 |
| C11.0 | SG | 3017~2823/1805~1734 | PLSR | Fat | 0.375 | 1.282 | 0.511 | 1.440 |
| C11.0 | MSC | 3017~2823/1805~1734 | LassoR | Milk | 0.493 | 1.407 | 0.405 | 1.292 |
| C11.0 | DER1 | 3017~2823/1805~1734 | LassoR | Milk | 0.569 | 1.522 | 0.491 | 1.407 |
| C11.0 | DER2 | 3017~2823/1805~1734 | LassoR | Milk | 0.574 | 1.531 | 0.525 | 1.460 |
| C11.0 | SNV | 3017~2823/1805~1734 | LassoR | Milk | 0.549 | 1.490 | 0.463 | 1.365 |
| C11.0 | SG | 3017~2823/1805~1734 | LassoR | Milk | 0.483 | 1.392 | 0.476 | 1.387 |
| C11.0 | MSC | 3017~2823/1805~1734 | PLSR | Milk | 0.446 | 1.349 | 0.395 | 1.294 |
| C11.0 | DER1 | 3017~2823/1805~1734 | PLSR | Milk | 0.364 | 1.274 | 0.484 | 1.401 |
| C11.0 | DER2 | 3017~2823/1805~1734 | PLSR | Milk | 0.393 | 1.297 | 0.520 | 1.452 |
| C11.0 | SNV | 3017~2823/1805~1734 | PLSR | Milk | 0.435 | 1.337 | 0.436 | 1.340 |
| C11.0 | SG | 3017~2823/1805~1734 | PLSR | Milk | 0.417 | 1.326 | 0.456 | 1.364 |
| C11.0 | MSC | 3017~2823/1805~1734 | RFR | Milk | 0.317 | 1.198 | 0.368 | 1.230 |
| C11.0 | DER1 | 3017~2823/1805~1734 | RFR | Milk | 0.372 | 1.260 | 0.352 | 1.190 |
| C11.0 | DER2 | 3017~2823/1805~1734 | RFR | Milk | 0.368 | 1.257 | 0.449 | 1.320 |
| C11.0 | SNV | 3017~2823/1805~1734 | RFR | Milk | 0.314 | 1.194 | 0.363 | 1.226 |
| C11.0 | SG | 3017~2823/1805~1734 | RFR | Milk | 0.297 | 1.180 | 0.349 | 1.213 |
| C11.0 | MSC | 3017~2823/1805~1734 | RidgeR | Milk | 0.441 | 1.339 | 0.359 | 1.237 |
| C11.0 | DER1 | 3017~2823/1805~1734 | RidgeR | Milk | 0.442 | 1.340 | 0.435 | 1.331 |
| C11.0 | DER2 | 3017~2823/1805~1734 | RidgeR | Milk | 0.494 | 1.406 | 0.487 | 1.403 |
| C11.0 | SNV | 3017~2823/1805~1734 | RidgeR | Milk | 0.430 | 1.326 | 0.362 | 1.239 |
| C11.0 | SG | 3017~2823/1805~1734 | RidgeR | Milk | 0.372 | 1.264 | 0.346 | 1.234 |
| C11.0 | MSC | 4000~400 | RidgeR | Fat | 0.403 | 1.284 | 0.434 | 1.298 |
| C11.0 | DER1 | 4000~400 | RidgeR | Fat | 0.534 | 1.420 | 0.457 | 1.305 |
| C11.0 | DER2 | 4000~400 | RidgeR | Fat | 0.665 | 1.598 | 0.496 | 1.339 |
| C11.0 | SNV | 4000~400 | RidgeR | Fat | 0.402 | 1.281 | 0.421 | 1.283 |
| C11.0 | SG | 4000~400 | RidgeR | Fat | 0.385 | 1.266 | 0.408 | 1.264 |
| C11.0 | MSC | 4000~400 | RFR | Fat | 0.262 | 1.166 | 0.454 | 1.297 |
| C11.0 | DER1 | 4000~400 | RFR | Fat | 0.294 | 1.192 | 0.463 | 1.326 |
| C11.0 | DER2 | 4000~400 | RFR | Fat | 0.286 | 1.185 | 0.536 | 1.387 |
| C11.0 | SNV | 4000~400 | RFR | Fat | 0.259 | 1.163 | 0.467 | 1.336 |
| C11.0 | SG | 4000~400 | RFR | Fat | 0.232 | 1.140 | 0.453 | 1.302 |
| C11.0 | MSC | 4000~400 | PLSR | Fat | 0.250 | 1.167 | 0.269 | 1.177 |
| C11.0 | DER1 | 4000~400 | PLSR | Fat | 0.249 | 1.163 | 0.377 | 1.275 |
| C11.0 | DER2 | 4000~400 | PLSR | Fat | 0.280 | 1.186 | 0.393 | 1.292 |
| C11.0 | SNV | 4000~400 | PLSR | Fat | 0.244 | 1.165 | 0.277 | 1.183 |
| C11.0 | SG | 4000~400 | PLSR | Fat | 0.228 | 1.152 | 0.217 | 1.138 |
| C11.0 | MSC | 4000~400 | LassoR | Fat | 0.859 | 2.587 | 0.285 | 1.037 |
| C11.0 | DER1 | 4000~400 | LassoR | Fat | 0.975 | 5.740 | 0.286 | 1.010 |
| C11.0 | DER2 | 4000~400 | LassoR | Fat | 0.993 | 10.298 | 0.347 | 1.060 |
| C11.0 | SNV | 4000~400 | LassoR | Fat | 0.866 | 2.642 | 0.310 | 1.062 |
| C11.0 | SG | 4000~400 | LassoR | Fat | 0.782 | 2.113 | 0.259 | 1.017 |
| C11.0 | MSC | 4000~400 | LassoR | Milk | 0.856 | 2.583 | 0.357 | 1.177 |
| C11.0 | DER1 | 4000~400 | LassoR | Milk | 0.982 | 6.953 | 0.281 | 0.961 |
| C11.0 | DER2 | 4000~400 | LassoR | Milk | 0.998 | 19.870 | 0.301 | 1.069 |
| C11.0 | SNV | 4000~400 | LassoR | Milk | 0.857 | 2.599 | 0.363 | 1.180 |
| C11.0 | SG | 4000~400 | LassoR | Milk | 0.790 | 2.157 | 0.361 | 1.209 |
| C11.0 | MSC | 4000~400 | PLSR | Milk | 0.402 | 1.305 | 0.411 | 1.312 |
| C11.0 | DER1 | 4000~400 | PLSR | Milk | 0.386 | 1.320 | 0.240 | 1.154 |

| | | | | | | | | |
|---|---|---|---|---|---|---|---|---|
| C11.0 | DER2 | 4000~400 | PLSR | Milk | 0.432 | 1.378 | 0.306 | 1.208 |
| C11.0 | SNV | 4000~400 | PLSR | Milk | 0.410 | 1.313 | 0.446 | 1.352 |
| C11.0 | SG | 4000~400 | PLSR | Milk | 0.368 | 1.269 | 0.382 | 1.280 |
| C11.0 | MSC | 4000~400 | RFR | Milk | 0.321 | 1.212 | 0.420 | 1.308 |
| C11.0 | DER1 | 4000~400 | RFR | Milk | 0.416 | 1.311 | 0.441 | 1.322 |
| C11.0 | DER2 | 4000~400 | RFR | Milk | 0.416 | 1.311 | 0.457 | 1.343 |
| C11.0 | SNV | 4000~400 | RFR | Milk | 0.336 | 1.226 | 0.417 | 1.297 |
| C11.0 | SG | 4000~400 | RFR | Milk | 0.348 | 1.237 | 0.378 | 1.256 |
| C11.0 | MSC | 4000~400 | RidgeR | Milk | 0.476 | 1.366 | 0.388 | 1.277 |
| C11.0 | DER1 | 4000~400 | RidgeR | Milk | 0.615 | 1.565 | 0.452 | 1.347 |
| C11.0 | DER2 | 4000~400 | RidgeR | Milk | 0.696 | 1.709 | 0.457 | 1.351 |
| C11.0 | SNV | 4000~400 | RidgeR | Milk | 0.489 | 1.384 | 0.404 | 1.292 |
| C11.0 | SG | 4000~400 | RidgeR | Milk | 0.471 | 1.363 | 0.429 | 1.318 |
| C12.0 | MSC | 3017~2823/1805~1734 | LassoR | Fat | 0.535 | 1.468 | 0.266 | 1.161 |
| C12.0 | DER1 | 3017~2823/1805~1734 | LassoR | Fat | 0.580 | 1.543 | 0.205 | 1.118 |
| C12.0 | DER2 | 3017~2823/1805~1734 | LassoR | Fat | 0.568 | 1.522 | 0.214 | 1.130 |
| C12.0 | SNV | 3017~2823/1805~1734 | LassoR | Fat | 0.546 | 1.485 | 0.272 | 1.167 |
| C12.0 | SG | 3017~2823/1805~1734 | LassoR | Fat | 0.472 | 1.373 | 0.156 | 1.084 |
| C12.0 | MSC | 3017~2823/1805~1734 | RidgeR | Fat | 0.335 | 1.223 | 0.183 | 1.105 |
| C12.0 | DER1 | 3017~2823/1805~1734 | RidgeR | Fat | 0.422 | 1.310 | 0.157 | 1.090 |
| C12.0 | DER2 | 3017~2823/1805~1734 | RidgeR | Fat | 0.492 | 1.396 | 0.179 | 1.105 |
| C12.0 | SNV | 3017~2823/1805~1734 | RidgeR | Fat | 0.331 | 1.220 | 0.207 | 1.119 |
| C12.0 | SG | 3017~2823/1805~1734 | RidgeR | Fat | 0.264 | 1.163 | 0.124 | 1.072 |
| C12.0 | MSC | 3017~2823/1805~1734 | RFR | Fat | 0.204 | 1.119 | 0.127 | 1.063 |
| C12.0 | DER1 | 3017~2823/1805~1734 | RFR | Fat | 0.144 | 1.080 | 0.193 | 1.109 |
| C12.0 | DER2 | 3017~2823/1805~1734 | RFR | Fat | 0.191 | 1.113 | 0.192 | 1.112 |
| C12.0 | SNV | 3017~2823/1805~1734 | RFR | Fat | 0.196 | 1.113 | 0.112 | 1.053 |
| C12.0 | SG | 3017~2823/1805~1734 | RFR | Fat | 0.077 | 1.022 | 0.084 | 1.043 |
| C12.0 | MSC | 3017~2823/1805~1734 | PLSR | Fat | 0.440 | 1.347 | 0.220 | 1.139 |
| C12.0 | DER1 | 3017~2823/1805~1734 | PLSR | Fat | 0.439 | 1.345 | 0.164 | 1.101 |
| C12.0 | DER2 | 3017~2823/1805~1734 | PLSR | Fat | 0.386 | 1.288 | 0.181 | 1.112 |
| C12.0 | SNV | 3017~2823/1805~1734 | PLSR | Fat | 0.445 | 1.352 | 0.233 | 1.149 |
| C12.0 | SG | 3017~2823/1805~1734 | PLSR | Fat | 0.452 | 1.359 | 0.138 | 1.084 |
| C12.0 | MSC | 3017~2823/1805~1734 | LassoR | Milk | 0.732 | 1.936 | 0.791 | 2.193 |
| C12.0 | DER1 | 3017~2823/1805~1734 | LassoR | Milk | 0.790 | 2.182 | 0.841 | 2.495 |
| C12.0 | DER2 | 3017~2823/1805~1734 | LassoR | Milk | 0.780 | 2.132 | 0.833 | 2.417 |
| C12.0 | SNV | 3017~2823/1805~1734 | LassoR | Milk | 0.740 | 1.964 | 0.800 | 2.242 |
| C12.0 | SG | 3017~2823/1805~1734 | LassoR | Milk | 0.708 | 1.854 | 0.785 | 2.151 |
| C12.0 | MSC | 3017~2823/1805~1734 | PLSR | Milk | 0.681 | 1.779 | 0.759 | 2.050 |
| C12.0 | DER1 | 3017~2823/1805~1734 | PLSR | Milk | 0.596 | 1.607 | 0.815 | 2.340 |
| C12.0 | DER2 | 3017~2823/1805~1734 | PLSR | Milk | 0.623 | 1.649 | 0.785 | 2.170 |
| C12.0 | SNV | 3017~2823/1805~1734 | PLSR | Milk | 0.689 | 1.803 | 0.778 | 2.135 |
| C12.0 | SG | 3017~2823/1805~1734 | PLSR | Milk | 0.648 | 1.714 | 0.816 | 2.345 |
| C12.0 | MSC | 3017~2823/1805~1734 | RFR | Milk | 0.645 | 1.680 | 0.700 | 1.834 |
| C12.0 | DER1 | 3017~2823/1805~1734 | RFR | Milk | 0.663 | 1.725 | 0.719 | 1.895 |
| C12.0 | DER2 | 3017~2823/1805~1734 | RFR | Milk | 0.660 | 1.718 | 0.717 | 1.869 |
| C12.0 | SNV | 3017~2823/1805~1734 | RFR | Milk | 0.640 | 1.668 | 0.709 | 1.865 |
| C12.0 | SG | 3017~2823/1805~1734 | RFR | Milk | 0.618 | 1.618 | 0.687 | 1.782 |
| C12.0 | MSC | 3017~2823/1805~1734 | RidgeR | Milk | 0.667 | 1.734 | 0.751 | 1.992 |
| C12.0 | DER1 | 3017~2823/1805~1734 | RidgeR | Milk | 0.654 | 1.702 | 0.771 | 2.038 |

| | | | | | | | | |
|---|---|---|---|---|---|---|---|---|
| C12.0 | DER2 | 3017~2823/1805~1734 | RidgeR | Milk | 0.689 | 1.792 | 0.792 | 2.143 |
| C12.0 | SNV | 3017~2823/1805~1734 | RidgeR | Milk | 0.667 | 1.734 | 0.748 | 1.983 |
| C12.0 | SG | 3017~2823/1805~1734 | RidgeR | Milk | 0.588 | 1.560 | 0.716 | 1.834 |
| C12.0 | MSC | 4000~400 | RidgeR | Fat | 0.411 | 1.261 | 0.138 | 1.082 |
| C12.0 | DER1 | 4000~400 | RidgeR | Fat | 0.544 | 1.414 | 0.194 | 1.117 |
| C12.0 | DER2 | 4000~400 | RidgeR | Fat | 0.689 | 1.598 | 0.178 | 1.105 |
| C12.0 | SNV | 4000~400 | RidgeR | Fat | 0.412 | 1.266 | 0.176 | 1.104 |
| C12.0 | SG | 4000~400 | RidgeR | Fat | 0.390 | 1.248 | 0.132 | 1.078 |
| C12.0 | MSC | 4000~400 | RFR | Fat | 0.158 | 1.090 | 0.152 | 1.091 |
| C12.0 | DER1 | 4000~400 | RFR | Fat | 0.283 | 1.182 | 0.117 | 1.070 |
| C12.0 | DER2 | 4000~400 | RFR | Fat | 0.251 | 1.158 | 0.138 | 1.082 |
| C12.0 | SNV | 4000~400 | RFR | Fat | 0.132 | 1.066 | 0.126 | 1.074 |
| C12.0 | SG | 4000~400 | RFR | Fat | 0.140 | 1.077 | 0.118 | 1.070 |
| C12.0 | MSC | 4000~400 | PLSR | Fat | 0.277 | 1.192 | 0.111 | 1.067 |
| C12.0 | DER1 | 4000~400 | PLSR | Fat | 0.275 | 1.205 | 0.150 | 1.091 |
| C12.0 | DER2 | 4000~400 | PLSR | Fat | 0.273 | 1.195 | 0.151 | 1.092 |
| C12.0 | SNV | 4000~400 | PLSR | Fat | 0.275 | 1.189 | 0.141 | 1.086 |
| C12.0 | SG | 4000~400 | PLSR | Fat | 0.211 | 1.142 | 0.108 | 1.066 |
| C12.0 | MSC | 4000~400 | LassoR | Fat | 0.898 | 3.062 | 0.153 | 0.977 |
| C12.0 | DER1 | 4000~400 | LassoR | Fat | 0.953 | 4.255 | 0.161 | 1.008 |
| C12.0 | DER2 | 4000~400 | LassoR | Fat | 0.997 | 15.942 | 0.083 | 0.942 |
| C12.0 | SNV | 4000~400 | LassoR | Fat | 0.889 | 2.932 | 0.157 | 0.984 |
| C12.0 | SG | 4000~400 | LassoR | Fat | 0.816 | 2.280 | 0.167 | 1.024 |
| C12.0 | MSC | 4000~400 | LassoR | Milk | 0.904 | 3.195 | 0.738 | 1.918 |
| C12.0 | DER1 | 4000~400 | LassoR | Milk | 0.970 | 5.592 | 0.721 | 1.871 |
| C12.0 | DER2 | 4000~400 | LassoR | Milk | 0.998 | 19.128 | 0.684 | 1.738 |
| C12.0 | SNV | 4000~400 | LassoR | Milk | 0.907 | 3.264 | 0.723 | 1.853 |
| C12.0 | SG | 4000~400 | LassoR | Milk | 0.864 | 2.697 | 0.760 | 2.049 |
| C12.0 | MSC | 4000~400 | PLSR | Milk | 0.591 | 1.583 | 0.767 | 2.086 |
| C12.0 | DER1 | 4000~400 | PLSR | Milk | 0.587 | 1.568 | 0.779 | 2.142 |
| C12.0 | DER2 | 4000~400 | PLSR | Milk | 0.608 | 1.618 | 0.774 | 2.116 |
| C12.0 | SNV | 4000~400 | PLSR | Milk | 0.634 | 1.662 | 0.703 | 1.847 |
| C12.0 | SG | 4000~400 | PLSR | Milk | 0.544 | 1.499 | 0.752 | 2.021 |
| C12.0 | MSC | 4000~400 | RFR | Milk | 0.616 | 1.614 | 0.725 | 1.899 |
| C12.0 | DER1 | 4000~400 | RFR | Milk | 0.676 | 1.760 | 0.723 | 1.877 |
| C12.0 | DER2 | 4000~400 | RFR | Milk | 0.698 | 1.818 | 0.708 | 1.824 |
| C12.0 | SNV | 4000~400 | RFR | Milk | 0.621 | 1.626 | 0.717 | 1.860 |
| C12.0 | SG | 4000~400 | RFR | Milk | 0.632 | 1.650 | 0.705 | 1.830 |
| C12.0 | MSC | 4000~400 | RidgeR | Milk | 0.642 | 1.661 | 0.748 | 1.912 |
| C12.0 | DER1 | 4000~400 | RidgeR | Milk | 0.709 | 1.837 | 0.778 | 2.031 |
| C12.0 | DER2 | 4000~400 | RidgeR | Milk | 0.772 | 2.030 | 0.789 | 2.029 |
| C12.0 | SNV | 4000~400 | RidgeR | Milk | 0.684 | 1.764 | 0.734 | 1.914 |
| C12.0 | SG | 4000~400 | RidgeR | Milk | 0.627 | 1.632 | 0.742 | 1.887 |
| C13.0 | MSC | 3017~2823/1805~1734 | LassoR | Fat | 0.547 | 1.487 | 0.405 | 1.286 |
| C13.0 | DER1 | 3017~2823/1805~1734 | LassoR | Fat | 0.584 | 1.550 | 0.389 | 1.267 |
| C13.0 | DER2 | 3017~2823/1805~1734 | LassoR | Fat | 0.579 | 1.543 | 0.373 | 1.238 |
| C13.0 | SNV | 3017~2823/1805~1734 | LassoR | Fat | 0.557 | 1.504 | 0.418 | 1.301 |
| C13.0 | SG | 3017~2823/1805~1734 | LassoR | Fat | 0.516 | 1.437 | 0.385 | 1.262 |
| C13.0 | MSC | 3017~2823/1805~1734 | RidgeR | Fat | 0.395 | 1.287 | 0.329 | 1.226 |
| C13.0 | DER1 | 3017~2823/1805~1734 | RidgeR | Fat | 0.454 | 1.353 | 0.341 | 1.230 |

| | | | | | | | | |
|---|---|---|---|---|---|---|---|---|
| C13.0 | DER2 | 3017~2823/1805~1734 | RidgeR | Fat | 0.485 | 1.393 | 0.331 | 1.214 |
| C13.0 | SNV | 3017~2823/1805~1734 | RidgeR | Fat | 0.387 | 1.279 | 0.333 | 1.232 |
| C13.0 | SG | 3017~2823/1805~1734 | RidgeR | Fat | 0.369 | 1.262 | 0.268 | 1.165 |
| C13.0 | MSC | 3017~2823/1805~1734 | RFR | Fat | 0.263 | 1.155 | 0.397 | 1.292 |
| C13.0 | DER1 | 3017~2823/1805~1734 | RFR | Fat | 0.265 | 1.158 | 0.340 | 1.224 |
| C13.0 | DER2 | 3017~2823/1805~1734 | RFR | Fat | 0.304 | 1.199 | 0.405 | 1.299 |
| C13.0 | SNV | 3017~2823/1805~1734 | RFR | Fat | 0.285 | 1.174 | 0.334 | 1.228 |
| C13.0 | SG | 3017~2823/1805~1734 | RFR | Fat | 0.290 | 1.182 | 0.290 | 1.183 |
| C13.0 | MSC | 3017~2823/1805~1734 | PLSR | Fat | 0.432 | 1.337 | 0.400 | 1.299 |
| C13.0 | DER1 | 3017~2823/1805~1734 | PLSR | Fat | 0.395 | 1.303 | 0.397 | 1.296 |
| C13.0 | DER2 | 3017~2823/1805~1734 | PLSR | Fat | 0.392 | 1.289 | 0.291 | 1.196 |
| C13.0 | SNV | 3017~2823/1805~1734 | PLSR | Fat | 0.430 | 1.334 | 0.408 | 1.308 |
| C13.0 | SG | 3017~2823/1805~1734 | PLSR | Fat | 0.434 | 1.343 | 0.330 | 1.229 |
| C13.0 | MSC | 3017~2823/1805~1734 | LassoR | Milk | 0.458 | 1.360 | 0.553 | 1.483 |
| C13.0 | DER1 | 3017~2823/1805~1734 | LassoR | Milk | 0.413 | 1.306 | 0.544 | 1.448 |
| C13.0 | DER2 | 3017~2823/1805~1734 | LassoR | Milk | 0.507 | 1.424 | 0.576 | 1.516 |
| C13.0 | SNV | 3017~2823/1805~1734 | LassoR | Milk | 0.426 | 1.321 | 0.522 | 1.442 |
| C13.0 | SG | 3017~2823/1805~1734 | LassoR | Milk | 0.372 | 1.263 | 0.570 | 1.460 |
| C13.0 | MSC | 3017~2823/1805~1734 | PLSR | Milk | 0.325 | 1.228 | 0.559 | 1.515 |
| C13.0 | DER1 | 3017~2823/1805~1734 | PLSR | Milk | 0.293 | 1.195 | 0.523 | 1.457 |
| C13.0 | DER2 | 3017~2823/1805~1734 | PLSR | Milk | 0.259 | 1.175 | 0.512 | 1.440 |
| C13.0 | SNV | 3017~2823/1805~1734 | PLSR | Milk | 0.312 | 1.217 | 0.558 | 1.514 |
| C13.0 | SG | 3017~2823/1805~1734 | PLSR | Milk | 0.240 | 1.163 | 0.659 | 1.722 |
| C13.0 | MSC | 3017~2823/1805~1734 | RFR | Milk | 0.250 | 1.147 | 0.481 | 1.390 |
| C13.0 | DER1 | 3017~2823/1805~1734 | RFR | Milk | 0.275 | 1.171 | 0.542 | 1.464 |
| C13.0 | DER2 | 3017~2823/1805~1734 | RFR | Milk | 0.261 | 1.156 | 0.520 | 1.435 |
| C13.0 | SNV | 3017~2823/1805~1734 | RFR | Milk | 0.244 | 1.139 | 0.495 | 1.407 |
| C13.0 | SG | 3017~2823/1805~1734 | RFR | Milk | 0.191 | 1.092 | 0.496 | 1.395 |
| C13.0 | MSC | 3017~2823/1805~1734 | RidgeR | Milk | 0.357 | 1.249 | 0.432 | 1.333 |
| C13.0 | DER1 | 3017~2823/1805~1734 | RidgeR | Milk | 0.374 | 1.264 | 0.536 | 1.423 |
| C13.0 | DER2 | 3017~2823/1805~1734 | RidgeR | Milk | 0.383 | 1.272 | 0.500 | 1.381 |
| C13.0 | SNV | 3017~2823/1805~1734 | RidgeR | Milk | 0.360 | 1.252 | 0.439 | 1.341 |
| C13.0 | SG | 3017~2823/1805~1734 | RidgeR | Milk | 0.299 | 1.197 | 0.436 | 1.320 |
| C13.0 | MSC | 4000~400 | RidgeR | Fat | 0.441 | 1.331 | 0.305 | 1.203 |
| C13.0 | DER1 | 4000~400 | RidgeR | Fat | 0.549 | 1.460 | 0.275 | 1.164 |
| C13.0 | DER2 | 4000~400 | RidgeR | Fat | 0.655 | 1.606 | 0.332 | 1.222 |
| C13.0 | SNV | 4000~400 | RidgeR | Fat | 0.438 | 1.327 | 0.307 | 1.205 |
| C13.0 | SG | 4000~400 | RidgeR | Fat | 0.436 | 1.328 | 0.253 | 1.153 |
| C13.0 | MSC | 4000~400 | RFR | Fat | 0.308 | 1.203 | 0.352 | 1.247 |
| C13.0 | DER1 | 4000~400 | RFR | Fat | 0.358 | 1.250 | 0.381 | 1.278 |
| C13.0 | DER2 | 4000~400 | RFR | Fat | 0.345 | 1.237 | 0.408 | 1.305 |
| C13.0 | SNV | 4000~400 | RFR | Fat | 0.327 | 1.220 | 0.382 | 1.279 |
| C13.0 | SG | 4000~400 | RFR | Fat | 0.322 | 1.216 | 0.303 | 1.200 |
| C13.0 | MSC | 4000~400 | PLSR | Fat | 0.338 | 1.245 | 0.094 | 1.057 |
| C13.0 | DER1 | 4000~400 | PLSR | Fat | 0.325 | 1.222 | 0.242 | 1.156 |
| C13.0 | DER2 | 4000~400 | PLSR | Fat | 0.324 | 1.223 | 0.271 | 1.178 |
| C13.0 | SNV | 4000~400 | PLSR | Fat | 0.321 | 1.233 | 0.102 | 1.062 |
| C13.0 | SG | 4000~400 | PLSR | Fat | 0.314 | 1.223 | 0.001 | 1.007 |
| C13.0 | MSC | 4000~400 | LassoR | Fat | 0.852 | 2.540 | 0.206 | 1.001 |
| C13.0 | DER1 | 4000~400 | LassoR | Fat | 0.947 | 4.031 | 0.199 | 1.000 |

| | | | | | | | | |
|---|---|---|---|---|---|---|---|---|
| C13.0 | DER2 | 4000~400 | LassoR | Fat | 0.994 | 11.337 | 0.218 | 0.932 |
| C13.0 | SNV | 4000~400 | LassoR | Fat | 0.854 | 2.557 | 0.208 | 1.008 |
| C13.0 | SG | 4000~400 | LassoR | Fat | 0.757 | 2.005 | 0.186 | 0.995 |
| C13.0 | MSC | 4000~400 | LassoR | Milk | 0.826 | 2.330 | 0.388 | 1.201 |
| C13.0 | DER1 | 4000~400 | LassoR | Milk | 0.963 | 4.788 | 0.413 | 1.125 |
| C13.0 | DER2 | 4000~400 | LassoR | Milk | 0.994 | 11.512 | 0.436 | 1.253 |
| C13.0 | SNV | 4000~400 | LassoR | Milk | 0.838 | 2.419 | 0.385 | 1.170 |
| C13.0 | SG | 4000~400 | LassoR | Milk | 0.762 | 2.009 | 0.412 | 1.259 |
| C13.0 | MSC | 4000~400 | PLSR | Milk | 0.264 | 1.179 | 0.423 | 1.325 |
| C13.0 | DER1 | 4000~400 | PLSR | Milk | 0.288 | 1.194 | 0.441 | 1.346 |
| C13.0 | DER2 | 4000~400 | PLSR | Milk | 0.298 | 1.218 | 0.516 | 1.446 |
| C13.0 | SNV | 4000~400 | PLSR | Milk | 0.283 | 1.198 | 0.464 | 1.375 |
| C13.0 | SG | 4000~400 | PLSR | Milk | 0.237 | 1.156 | 0.423 | 1.325 |
| C13.0 | MSC | 4000~400 | RFR | Milk | 0.286 | 1.181 | 0.502 | 1.403 |
| C13.0 | DER1 | 4000~400 | RFR | Milk | 0.335 | 1.227 | 0.582 | 1.510 |
| C13.0 | DER2 | 4000~400 | RFR | Milk | 0.354 | 1.246 | 0.555 | 1.458 |
| C13.0 | SNV | 4000~400 | RFR | Milk | 0.302 | 1.197 | 0.532 | 1.436 |
| C13.0 | SG | 4000~400 | RFR | Milk | 0.291 | 1.182 | 0.537 | 1.448 |
| C13.0 | MSC | 4000~400 | RidgeR | Milk | 0.418 | 1.301 | 0.441 | 1.336 |
| C13.0 | DER1 | 4000~400 | RidgeR | Milk | 0.535 | 1.435 | 0.520 | 1.427 |
| C13.0 | DER2 | 4000~400 | RidgeR | Milk | 0.649 | 1.592 | 0.554 | 1.460 |
| C13.0 | SNV | 4000~400 | RidgeR | Milk | 0.434 | 1.317 | 0.477 | 1.378 |
| C13.0 | SG | 4000~400 | RidgeR | Milk | 0.404 | 1.286 | 0.479 | 1.362 |
| C14.0 | MSC | 3017~2823/1805~1734 | LassoR | Fat | 0.367 | 1.257 | 0.397 | 1.296 |
| C14.0 | DER1 | 3017~2823/1805~1734 | LassoR | Fat | 0.364 | 1.250 | 0.410 | 1.303 |
| C14.0 | DER2 | 3017~2823/1805~1734 | LassoR | Fat | 0.390 | 1.277 | 0.363 | 1.259 |
| C14.0 | SNV | 3017~2823/1805~1734 | LassoR | Fat | 0.367 | 1.257 | 0.399 | 1.298 |
| C14.0 | SG | 3017~2823/1805~1734 | LassoR | Fat | 0.361 | 1.248 | 0.397 | 1.290 |
| C14.0 | MSC | 3017~2823/1805~1734 | RidgeR | Fat | 0.202 | 1.113 | 0.213 | 1.122 |
| C14.0 | DER1 | 3017~2823/1805~1734 | RidgeR | Fat | 0.236 | 1.141 | 0.303 | 1.191 |
| C14.0 | DER2 | 3017~2823/1805~1734 | RidgeR | Fat | 0.294 | 1.183 | 0.354 | 1.225 |
| C14.0 | SNV | 3017~2823/1805~1734 | RidgeR | Fat | 0.193 | 1.107 | 0.214 | 1.119 |
| C14.0 | SG | 3017~2823/1805~1734 | RidgeR | Fat | 0.129 | 1.073 | 0.114 | 1.062 |
| C14.0 | MSC | 3017~2823/1805~1734 | RFR | Fat | 0.023 | 0.970 | 0.075 | 1.031 |
| C14.0 | DER1 | 3017~2823/1805~1734 | RFR | Fat | 0.040 | 0.995 | 0.035 | 0.993 |
| C14.0 | DER2 | 3017~2823/1805~1734 | RFR | Fat | 0.027 | 0.998 | 0.210 | 1.121 |
| C14.0 | SNV | 3017~2823/1805~1734 | RFR | Fat | 0.029 | 0.982 | 0.085 | 1.041 |
| C14.0 | SG | 3017~2823/1805~1734 | RFR | Fat | 0.035 | 0.989 | 0.039 | 0.996 |
| C14.0 | MSC | 3017~2823/1805~1734 | PLSR | Fat | 0.250 | 1.161 | 0.403 | 1.302 |
| C14.0 | DER1 | 3017~2823/1805~1734 | PLSR | Fat | 0.160 | 1.100 | 0.434 | 1.338 |
| C14.0 | DER2 | 3017~2823/1805~1734 | PLSR | Fat | 0.125 | 1.079 | 0.332 | 1.231 |
| C14.0 | SNV | 3017~2823/1805~1734 | PLSR | Fat | 0.256 | 1.165 | 0.375 | 1.273 |
| C14.0 | SG | 3017~2823/1805~1734 | PLSR | Fat | 0.223 | 1.142 | 0.393 | 1.292 |
| C14.0 | MSC | 3017~2823/1805~1734 | LassoR | Milk | 0.767 | 2.078 | 0.710 | 1.853 |
| C14.0 | DER1 | 3017~2823/1805~1734 | LassoR | Milk | 0.782 | 2.147 | 0.644 | 1.629 |
| C14.0 | DER2 | 3017~2823/1805~1734 | LassoR | Milk | 0.773 | 2.100 | 0.626 | 1.589 |
| C14.0 | SNV | 3017~2823/1805~1734 | LassoR | Milk | 0.770 | 2.090 | 0.719 | 1.888 |
| C14.0 | SG | 3017~2823/1805~1734 | LassoR | Milk | 0.744 | 1.979 | 0.614 | 1.550 |
| C14.0 | MSC | 3017~2823/1805~1734 | PLSR | Milk | 0.727 | 1.922 | 0.701 | 1.840 |
| C14.0 | DER1 | 3017~2823/1805~1734 | PLSR | Milk | 0.688 | 1.807 | 0.558 | 1.514 |

| | | | | | | | | | |
|---|---|---|---|---|---|---|---|---|---|
| C14.0 | DER2 | 3017~2823/1805~1734 | PLSR | Milk | 0.682 | 1.790 | 0.602 | 1.595 |
| C14.0 | SNV | 3017~2823/1805~1734 | PLSR | Milk | 0.728 | 1.924 | 0.719 | 1.898 |
| C14.0 | SG | 3017~2823/1805~1734 | PLSR | Milk | 0.712 | 1.873 | 0.584 | 1.560 |
| C14.0 | MSC | 3017~2823/1805~1734 | RFR | Milk | 0.634 | 1.653 | 0.755 | 1.948 |
| C14.0 | DER1 | 3017~2823/1805~1734 | RFR | Milk | 0.650 | 1.688 | 0.741 | 1.896 |
| C14.0 | DER2 | 3017~2823/1805~1734 | RFR | Milk | 0.663 | 1.725 | 0.731 | 1.853 |
| C14.0 | SNV | 3017~2823/1805~1734 | RFR | Milk | 0.635 | 1.656 | 0.757 | 1.968 |
| C14.0 | SG | 3017~2823/1805~1734 | RFR | Milk | 0.622 | 1.626 | 0.718 | 1.840 |
| C14.0 | MSC | 3017~2823/1805~1734 | RidgeR | Milk | 0.678 | 1.765 | 0.701 | 1.811 |
| C14.0 | DER1 | 3017~2823/1805~1734 | RidgeR | Milk | 0.709 | 1.855 | 0.609 | 1.569 |
| C14.0 | DER2 | 3017~2823/1805~1734 | RidgeR | Milk | 0.733 | 1.938 | 0.604 | 1.542 |
| C14.0 | SNV | 3017~2823/1805~1734 | RidgeR | Milk | 0.674 | 1.754 | 0.710 | 1.827 |
| C14.0 | SG | 3017~2823/1805~1734 | RidgeR | Milk | 0.644 | 1.680 | 0.595 | 1.552 |
| C14.0 | MSC | 4000~400 | RidgeR | Fat | 0.145 | 1.080 | 0.093 | 1.050 |
| C14.0 | DER1 | 4000~400 | RidgeR | Fat | 0.428 | 1.261 | 0.117 | 1.066 |
| C14.0 | DER2 | 4000~400 | RidgeR | Fat | 0.516 | 1.299 | 0.136 | 1.077 |
| C14.0 | SNV | 4000~400 | RidgeR | Fat | 0.146 | 1.079 | 0.093 | 1.050 |
| C14.0 | SG | 4000~400 | RidgeR | Fat | 0.162 | 1.090 | 0.085 | 1.046 |
| C14.0 | MSC | 4000~400 | RFR | Fat | 0.064 | 1.030 | 0.119 | 1.069 |
| C14.0 | DER1 | 4000~400 | RFR | Fat | 0.113 | 1.061 | 0.156 | 1.093 |
| C14.0 | DER2 | 4000~400 | RFR | Fat | 0.108 | 1.060 | 0.112 | 1.063 |
| C14.0 | SNV | 4000~400 | RFR | Fat | 0.056 | 1.024 | 0.129 | 1.073 |
| C14.0 | SG | 4000~400 | RFR | Fat | 0.060 | 1.022 | 0.076 | 1.040 |
| C14.0 | MSC | 4000~400 | PLSR | Fat | 0.059 | 1.047 | 0.053 | 1.034 |
| C14.0 | DER1 | 4000~400 | PLSR | Fat | 0.088 | 1.050 | 0.102 | 1.062 |
| C14.0 | DER2 | 4000~400 | PLSR | Fat | 0.073 | 1.047 | 0.082 | 1.050 |
| C14.0 | SNV | 4000~400 | PLSR | Fat | 0.052 | 1.031 | 0.094 | 1.057 |
| C14.0 | SG | 4000~400 | PLSR | Fat | 0.074 | 1.042 | 0.055 | 1.035 |
| C14.0 | MSC | 4000~400 | LassoR | Fat | 0.681 | 1.682 | 0.172 | 1.072 |
| C14.0 | DER1 | 4000~400 | LassoR | Fat | 0.887 | 2.665 | 0.048 | 0.942 |
| C14.0 | DER2 | 4000~400 | LassoR | Fat | 0.986 | 6.671 | 0.031 | 0.879 |
| C14.0 | SNV | 4000~400 | LassoR | Fat | 0.657 | 1.622 | 0.168 | 1.078 |
| C14.0 | SG | 4000~400 | LassoR | Fat | 0.585 | 1.497 | 0.140 | 1.055 |
| C14.0 | MSC | 4000~400 | LassoR | Milk | 0.920 | 3.514 | 0.615 | 1.583 |
| C14.0 | DER1 | 4000~400 | LassoR | Milk | 0.985 | 7.993 | 0.592 | 1.509 |
| C14.0 | DER2 | 4000~400 | LassoR | Milk | 0.997 | 18.127 | 0.592 | 1.513 |
| C14.0 | SNV | 4000~400 | LassoR | Milk | 0.917 | 3.450 | 0.631 | 1.636 |
| C14.0 | SG | 4000~400 | LassoR | Milk | 0.857 | 2.641 | 0.608 | 1.566 |
| C14.0 | MSC | 4000~400 | PLSR | Milk | 0.620 | 1.628 | 0.620 | 1.633 |
| C14.0 | DER1 | 4000~400 | PLSR | Milk | 0.631 | 1.653 | 0.611 | 1.614 |
| C14.0 | DER2 | 4000~400 | PLSR | Milk | 0.643 | 1.689 | 0.665 | 1.739 |
| C14.0 | SNV | 4000~400 | PLSR | Milk | 0.639 | 1.694 | 0.664 | 1.737 |
| C14.0 | SG | 4000~400 | PLSR | Milk | 0.620 | 1.629 | 0.583 | 1.558 |
| C14.0 | MSC | 4000~400 | RFR | Milk | 0.643 | 1.677 | 0.738 | 1.884 |
| C14.0 | DER1 | 4000~400 | RFR | Milk | 0.662 | 1.722 | 0.782 | 2.047 |
| C14.0 | DER2 | 4000~400 | RFR | Milk | 0.663 | 1.725 | 0.775 | 2.004 |
| C14.0 | SNV | 4000~400 | RFR | Milk | 0.644 | 1.679 | 0.750 | 1.945 |
| C14.0 | SG | 4000~400 | RFR | Milk | 0.677 | 1.762 | 0.761 | 1.955 |
| C14.0 | MSC | 4000~400 | RidgeR | Milk | 0.674 | 1.744 | 0.648 | 1.671 |
| C14.0 | DER1 | 4000~400 | RidgeR | Milk | 0.727 | 1.903 | 0.656 | 1.690 |

| | | | | | | | | |
|---|---|---|---|---|---|---|---|---|
| C14.0 | DER2 | 4000~400 | RidgeR | Milk | 0.778 | 2.075 | 0.674 | 1.731 |
| C14.0 | SNV | 4000~400 | RidgeR | Milk | 0.689 | 1.780 | 0.681 | 1.734 |
| C14.0 | SG | 4000~400 | RidgeR | Milk | 0.672 | 1.744 | 0.622 | 1.611 |
| C14.1 | MSC | 3017~2823/1805~1734 | LassoR | Fat | 0.502 | 1.419 | 0.613 | 1.603 |
| C14.1 | DER1 | 3017~2823/1805~1734 | LassoR | Fat | 0.487 | 1.395 | 0.572 | 1.524 |
| C14.1 | DER2 | 3017~2823/1805~1734 | LassoR | Fat | 0.468 | 1.370 | 0.483 | 1.397 |
| C14.1 | SNV | 3017~2823/1805~1734 | LassoR | Fat | 0.471 | 1.376 | 0.555 | 1.504 |
| C14.1 | SG | 3017~2823/1805~1734 | LassoR | Fat | 0.430 | 1.326 | 0.544 | 1.488 |
| C14.1 | MSC | 3017~2823/1805~1734 | RidgeR | Fat | 0.364 | 1.255 | 0.482 | 1.391 |
| C14.1 | DER1 | 3017~2823/1805~1734 | RidgeR | Fat | 0.344 | 1.235 | 0.387 | 1.284 |
| C14.1 | DER2 | 3017~2823/1805~1734 | RidgeR | Fat | 0.385 | 1.275 | 0.419 | 1.316 |
| C14.1 | SNV | 3017~2823/1805~1734 | RidgeR | Fat | 0.365 | 1.257 | 0.457 | 1.363 |
| C14.1 | SG | 3017~2823/1805~1734 | RidgeR | Fat | 0.354 | 1.246 | 0.435 | 1.337 |
| C14.1 | MSC | 3017~2823/1805~1734 | RFR | Fat | 0.222 | 1.123 | 0.326 | 1.214 |
| C14.1 | DER1 | 3017~2823/1805~1734 | RFR | Fat | 0.150 | 1.078 | 0.283 | 1.178 |
| C14.1 | DER2 | 3017~2823/1805~1734 | RFR | Fat | 0.169 | 1.097 | 0.407 | 1.304 |
| C14.1 | SNV | 3017~2823/1805~1734 | RFR | Fat | 0.190 | 1.099 | 0.338 | 1.226 |
| C14.1 | SG | 3017~2823/1805~1734 | RFR | Fat | 0.227 | 1.133 | 0.208 | 1.109 |
| C14.1 | MSC | 3017~2823/1805~1734 | PLSR | Fat | 0.382 | 1.282 | 0.618 | 1.628 |
| C14.1 | DER1 | 3017~2823/1805~1734 | PLSR | Fat | 0.278 | 1.190 | 0.544 | 1.490 |
| C14.1 | DER2 | 3017~2823/1805~1734 | PLSR | Fat | 0.271 | 1.185 | 0.437 | 1.341 |
| C14.1 | SNV | 3017~2823/1805~1734 | PLSR | Fat | 0.391 | 1.287 | 0.529 | 1.467 |
| C14.1 | SG | 3017~2823/1805~1734 | PLSR | Fat | 0.354 | 1.249 | 0.516 | 1.446 |
| C14.1 | MSC | 3017~2823/1805~1734 | LassoR | Milk | 0.619 | 1.623 | 0.479 | 1.325 |
| C14.1 | DER1 | 3017~2823/1805~1734 | LassoR | Milk | 0.633 | 1.650 | 0.340 | 1.126 |
| C14.1 | DER2 | 3017~2823/1805~1734 | LassoR | Milk | 0.611 | 1.604 | 0.376 | 1.221 |
| C14.1 | SNV | 3017~2823/1805~1734 | LassoR | Milk | 0.630 | 1.646 | 0.513 | 1.398 |
| C14.1 | SG | 3017~2823/1805~1734 | LassoR | Milk | 0.524 | 1.450 | 0.408 | 1.262 |
| C14.1 | MSC | 3017~2823/1805~1734 | PLSR | Milk | 0.551 | 1.500 | 0.401 | 1.300 |
| C14.1 | DER1 | 3017~2823/1805~1734 | PLSR | Milk | 0.477 | 1.399 | 0.246 | 1.159 |
| C14.1 | DER2 | 3017~2823/1805~1734 | PLSR | Milk | 0.392 | 1.298 | 0.381 | 1.279 |
| C14.1 | SNV | 3017~2823/1805~1734 | PLSR | Milk | 0.552 | 1.504 | 0.458 | 1.366 |
| C14.1 | SG | 3017~2823/1805~1734 | PLSR | Milk | 0.513 | 1.448 | 0.003 | 1.008 |
| C14.1 | MSC | 3017~2823/1805~1734 | RFR | Milk | 0.305 | 1.199 | 0.407 | 1.305 |
| C14.1 | DER1 | 3017~2823/1805~1734 | RFR | Milk | 0.246 | 1.149 | 0.284 | 1.180 |
| C14.1 | DER2 | 3017~2823/1805~1734 | RFR | Milk | 0.258 | 1.162 | 0.364 | 1.261 |
| C14.1 | SNV | 3017~2823/1805~1734 | RFR | Milk | 0.320 | 1.212 | 0.471 | 1.380 |
| C14.1 | SG | 3017~2823/1805~1734 | RFR | Milk | 0.234 | 1.137 | 0.436 | 1.337 |
| C14.1 | MSC | 3017~2823/1805~1734 | RidgeR | Milk | 0.451 | 1.349 | 0.461 | 1.366 |
| C14.1 | DER1 | 3017~2823/1805~1734 | RidgeR | Milk | 0.410 | 1.300 | 0.339 | 1.229 |
| C14.1 | DER2 | 3017~2823/1805~1734 | RidgeR | Milk | 0.450 | 1.347 | 0.338 | 1.218 |
| C14.1 | SNV | 3017~2823/1805~1734 | RidgeR | Milk | 0.452 | 1.352 | 0.473 | 1.382 |
| C14.1 | SG | 3017~2823/1805~1734 | RidgeR | Milk | 0.427 | 1.322 | 0.387 | 1.276 |
| C14.1 | MSC | 4000~400 | RidgeR | Fat | 0.389 | 1.274 | 0.401 | 1.299 |
| C14.1 | DER1 | 4000~400 | RidgeR | Fat | 0.535 | 1.438 | 0.418 | 1.317 |
| C14.1 | DER2 | 4000~400 | RidgeR | Fat | 0.677 | 1.650 | 0.406 | 1.303 |
| C14.1 | SNV | 4000~400 | RidgeR | Fat | 0.386 | 1.270 | 0.397 | 1.295 |
| C14.1 | SG | 4000~400 | RidgeR | Fat | 0.395 | 1.282 | 0.399 | 1.296 |
| C14.1 | MSC | 4000~400 | RFR | Fat | 0.310 | 1.204 | 0.448 | 1.354 |
| C14.1 | DER1 | 4000~400 | RFR | Fat | 0.295 | 1.192 | 0.462 | 1.371 |

| | | | | | | | | |
|---|---|---|---|---|---|---|---|---|
| C14.1 | DER2 | 4000~400 | RFR | Fat | 0.271 | 1.172 | 0.385 | 1.281 |
| C14.1 | SNV | 4000~400 | RFR | Fat | 0.327 | 1.220 | 0.444 | 1.350 |
| C14.1 | SG | 4000~400 | RFR | Fat | 0.259 | 1.163 | 0.411 | 1.311 |
| C14.1 | MSC | 4000~400 | PLSR | Fat | 0.315 | 1.213 | 0.398 | 1.297 |
| C14.1 | DER1 | 4000~400 | PLSR | Fat | 0.305 | 1.203 | 0.360 | 1.258 |
| C14.1 | DER2 | 4000~400 | PLSR | Fat | 0.279 | 1.213 | 0.375 | 1.273 |
| C14.1 | SNV | 4000~400 | PLSR | Fat | 0.313 | 1.210 | 0.395 | 1.294 |
| C14.1 | SG | 4000~400 | PLSR | Fat | 0.302 | 1.202 | 0.384 | 1.283 |
| C14.1 | MSC | 4000~400 | LassoR | Fat | 0.755 | 1.972 | 0.437 | 1.325 |
| C14.1 | DER1 | 4000~400 | LassoR | Fat | 0.969 | 5.274 | 0.265 | 1.027 |
| C14.1 | DER2 | 4000~400 | LassoR | Fat | 0.996 | 14.468 | 0.300 | 1.042 |
| C14.1 | SNV | 4000~400 | LassoR | Fat | 0.756 | 1.972 | 0.416 | 1.300 |
| C14.1 | SG | 4000~400 | LassoR | Fat | 0.699 | 1.791 | 0.447 | 1.334 |
| C14.1 | MSC | 4000~400 | LassoR | Milk | 0.836 | 2.410 | 0.407 | 1.241 |
| C14.1 | DER1 | 4000~400 | LassoR | Milk | 0.952 | 4.326 | 0.431 | 1.251 |
| C14.1 | DER2 | 4000~400 | LassoR | Milk | 0.992 | 10.064 | 0.378 | 1.196 |
| C14.1 | SNV | 4000~400 | LassoR | Milk | 0.836 | 2.400 | 0.418 | 1.263 |
| C14.1 | SG | 4000~400 | LassoR | Milk | 0.776 | 2.083 | 0.431 | 1.290 |
| C14.1 | MSC | 4000~400 | PLSR | Milk | 0.389 | 1.284 | 0.351 | 1.249 |
| C14.1 | DER1 | 4000~400 | PLSR | Milk | 0.372 | 1.268 | 0.289 | 1.193 |
| C14.1 | DER2 | 4000~400 | PLSR | Milk | 0.351 | 1.269 | 0.450 | 1.356 |
| C14.1 | SNV | 4000~400 | PLSR | Milk | 0.385 | 1.279 | 0.355 | 1.252 |
| C14.1 | SG | 4000~400 | PLSR | Milk | 0.370 | 1.264 | 0.319 | 1.219 |
| C14.1 | MSC | 4000~400 | RFR | Milk | 0.383 | 1.275 | 0.416 | 1.316 |
| C14.1 | DER1 | 4000~400 | RFR | Milk | 0.376 | 1.266 | 0.448 | 1.349 |
| C14.1 | DER2 | 4000~400 | RFR | Milk | 0.391 | 1.277 | 0.399 | 1.294 |
| C14.1 | SNV | 4000~400 | RFR | Milk | 0.383 | 1.276 | 0.459 | 1.367 |
| C14.1 | SG | 4000~400 | RFR | Milk | 0.380 | 1.271 | 0.445 | 1.345 |
| C14.1 | MSC | 4000~400 | RidgeR | Milk | 0.489 | 1.393 | 0.369 | 1.264 |
| C14.1 | DER1 | 4000~400 | RidgeR | Milk | 0.585 | 1.520 | 0.397 | 1.291 |
| C14.1 | DER2 | 4000~400 | RidgeR | Milk | 0.670 | 1.663 | 0.418 | 1.318 |
| C14.1 | SNV | 4000~400 | RidgeR | Milk | 0.482 | 1.383 | 0.367 | 1.262 |
| C14.1 | SG | 4000~400 | RidgeR | Milk | 0.479 | 1.375 | 0.356 | 1.251 |
| C15.0 | MSC | 3017~2823/1805~1734 | LassoR | Fat | 0.379 | 1.264 | 0.316 | 1.207 |
| C15.0 | DER1 | 3017~2823/1805~1734 | LassoR | Fat | 0.451 | 1.344 | 0.281 | 1.145 |
| C15.0 | DER2 | 3017~2823/1805~1734 | LassoR | Fat | 0.432 | 1.326 | 0.259 | 1.104 |
| C15.0 | SNV | 3017~2823/1805~1734 | LassoR | Fat | 0.393 | 1.278 | 0.320 | 1.210 |
| C15.0 | SG | 3017~2823/1805~1734 | LassoR | Fat | 0.398 | 1.285 | 0.257 | 1.135 |
| C15.0 | MSC | 3017~2823/1805~1734 | RidgeR | Fat | 0.165 | 1.095 | 0.168 | 1.100 |
| C15.0 | DER1 | 3017~2823/1805~1734 | RidgeR | Fat | 0.278 | 1.175 | 0.271 | 1.169 |
| C15.0 | DER2 | 3017~2823/1805~1734 | RidgeR | Fat | 0.318 | 1.208 | 0.266 | 1.158 |
| C15.0 | SNV | 3017~2823/1805~1734 | RidgeR | Fat | 0.160 | 1.092 | 0.162 | 1.097 |
| C15.0 | SG | 3017~2823/1805~1734 | RidgeR | Fat | 0.163 | 1.095 | 0.127 | 1.068 |
| C15.0 | MSC | 3017~2823/1805~1734 | RFR | Fat | 0.113 | 1.051 | 0.111 | 1.040 |
| C15.0 | DER1 | 3017~2823/1805~1734 | RFR | Fat | 0.077 | 1.034 | 0.114 | 1.048 |
| C15.0 | DER2 | 3017~2823/1805~1734 | RFR | Fat | 0.099 | 1.044 | 0.202 | 1.123 |
| C15.0 | SNV | 3017~2823/1805~1734 | RFR | Fat | 0.098 | 1.040 | 0.109 | 1.051 |
| C15.0 | SG | 3017~2823/1805~1734 | RFR | Fat | 0.083 | 1.030 | 0.085 | 1.016 |
| C15.0 | MSC | 3017~2823/1805~1734 | PLSR | Fat | 0.253 | 1.169 | 0.321 | 1.221 |
| C15.0 | DER1 | 3017~2823/1805~1734 | PLSR | Fat | 0.216 | 1.146 | 0.114 | 1.069 |

| | | | | | | | | |
|---|---|---|---|---|---|---|---|---|
| C15.0 | DER2 | 3017~2823/1805~1734 | PLSR | Fat | 0.177 | 1.111 | 0.087 | 1.053 |
| C15.0 | SNV | 3017~2823/1805~1734 | PLSR | Fat | 0.237 | 1.156 | 0.312 | 1.213 |
| C15.0 | SG | 3017~2823/1805~1734 | PLSR | Fat | 0.300 | 1.208 | 0.196 | 1.122 |
| C15.0 | MSC | 3017~2823/1805~1734 | LassoR | Milk | 0.579 | 1.543 | 0.500 | 1.416 |
| C15.0 | DER1 | 3017~2823/1805~1734 | LassoR | Milk | 0.650 | 1.691 | 0.552 | 1.499 |
| C15.0 | DER2 | 3017~2823/1805~1734 | LassoR | Milk | 0.621 | 1.625 | 0.498 | 1.418 |
| C15.0 | SNV | 3017~2823/1805~1734 | LassoR | Milk | 0.573 | 1.531 | 0.501 | 1.420 |
| C15.0 | SG | 3017~2823/1805~1734 | LassoR | Milk | 0.483 | 1.393 | 0.482 | 1.375 |
| C15.0 | MSC | 3017~2823/1805~1734 | PLSR | Milk | 0.459 | 1.377 | 0.552 | 1.503 |
| C15.0 | DER1 | 3017~2823/1805~1734 | PLSR | Milk | 0.392 | 1.302 | 0.516 | 1.446 |
| C15.0 | DER2 | 3017~2823/1805~1734 | PLSR | Milk | 0.404 | 1.312 | 0.485 | 1.402 |
| C15.0 | SNV | 3017~2823/1805~1734 | PLSR | Milk | 0.451 | 1.367 | 0.545 | 1.492 |
| C15.0 | SG | 3017~2823/1805~1734 | PLSR | Milk | 0.452 | 1.368 | 0.568 | 1.531 |
| C15.0 | MSC | 3017~2823/1805~1734 | RFR | Milk | 0.343 | 1.226 | 0.335 | 1.223 |
| C15.0 | DER1 | 3017~2823/1805~1734 | RFR | Milk | 0.383 | 1.270 | 0.464 | 1.375 |
| C15.0 | DER2 | 3017~2823/1805~1734 | RFR | Milk | 0.408 | 1.299 | 0.411 | 1.310 |
| C15.0 | SNV | 3017~2823/1805~1734 | RFR | Milk | 0.336 | 1.221 | 0.354 | 1.242 |
| C15.0 | SG | 3017~2823/1805~1734 | RFR | Milk | 0.357 | 1.233 | 0.348 | 1.244 |
| C15.0 | MSC | 3017~2823/1805~1734 | RidgeR | Milk | 0.425 | 1.320 | 0.433 | 1.332 |
| C15.0 | DER1 | 3017~2823/1805~1734 | RidgeR | Milk | 0.458 | 1.359 | 0.480 | 1.367 |
| C15.0 | DER2 | 3017~2823/1805~1734 | RidgeR | Milk | 0.511 | 1.430 | 0.470 | 1.364 |
| C15.0 | SNV | 3017~2823/1805~1734 | RidgeR | Milk | 0.423 | 1.319 | 0.444 | 1.346 |
| C15.0 | SG | 3017~2823/1805~1734 | RidgeR | Milk | 0.360 | 1.251 | 0.330 | 1.226 |
| C15.0 | MSC | 4000~400 | RidgeR | Fat | 0.300 | 1.177 | 0.117 | 1.064 |
| C15.0 | DER1 | 4000~400 | RidgeR | Fat | 0.486 | 1.322 | 0.171 | 1.091 |
| C15.0 | DER2 | 4000~400 | RidgeR | Fat | 0.586 | 1.386 | 0.229 | 1.140 |
| C15.0 | SNV | 4000~400 | RidgeR | Fat | 0.304 | 1.178 | 0.113 | 1.058 |
| C15.0 | SG | 4000~400 | RidgeR | Fat | 0.290 | 1.176 | 0.115 | 1.057 |
| C15.0 | MSC | 4000~400 | RFR | Fat | 0.113 | 1.059 | 0.154 | 1.090 |
| C15.0 | DER1 | 4000~400 | RFR | Fat | 0.151 | 1.087 | 0.132 | 1.073 |
| C15.0 | DER2 | 4000~400 | RFR | Fat | 0.147 | 1.082 | 0.133 | 1.075 |
| C15.0 | SNV | 4000~400 | RFR | Fat | 0.118 | 1.065 | 0.125 | 1.072 |
| C15.0 | SG | 4000~400 | RFR | Fat | 0.149 | 1.084 | 0.143 | 1.081 |
| C15.0 | MSC | 4000~400 | PLSR | Fat | 0.089 | 1.050 | 0.132 | 1.080 |
| C15.0 | DER1 | 4000~400 | PLSR | Fat | 0.118 | 1.068 | 0.083 | 1.051 |
| C15.0 | DER2 | 4000~400 | PLSR | Fat | 0.140 | 1.085 | 0.157 | 1.096 |
| C15.0 | SNV | 4000~400 | PLSR | Fat | 0.086 | 1.049 | 0.129 | 1.078 |
| C15.0 | SG | 4000~400 | PLSR | Fat | 0.115 | 1.067 | 0.083 | 1.051 |
| C15.0 | MSC | 4000~400 | LassoR | Fat | 0.772 | 1.997 | 0.081 | 0.903 |
| C15.0 | DER1 | 4000~400 | LassoR | Fat | 0.915 | 3.016 | 0.144 | 0.956 |
| C15.0 | DER2 | 4000~400 | LassoR | Fat | 0.993 | 9.966 | 0.159 | 0.957 |
| C15.0 | SNV | 4000~400 | LassoR | Fat | 0.777 | 2.024 | 0.085 | 0.897 |
| C15.0 | SG | 4000~400 | LassoR | Fat | 0.752 | 1.961 | 0.090 | 0.889 |
| C15.0 | MSC | 4000~400 | LassoR | Milk | 0.842 | 2.442 | 0.309 | 1.148 |
| C15.0 | DER1 | 4000~400 | LassoR | Milk | 0.974 | 5.666 | 0.282 | 1.074 |
| C15.0 | DER2 | 4000~400 | LassoR | Milk | 0.994 | 11.485 | 0.281 | 1.146 |
| C15.0 | SNV | 4000~400 | LassoR | Milk | 0.839 | 2.429 | 0.366 | 1.203 |
| C15.0 | SG | 4000~400 | LassoR | Milk | 0.751 | 1.976 | 0.374 | 1.251 |
| C15.0 | MSC | 4000~400 | PLSR | Milk | 0.285 | 1.199 | 0.369 | 1.267 |
| C15.0 | DER1 | 4000~400 | PLSR | Milk | 0.355 | 1.263 | 0.446 | 1.352 |

| | | | | | | | | |
|---|---|---|---|---|---|---|---|---|
| C15.0 | DER2 | 4000~400 | PLSR | Milk | 0.394 | 1.295 | 0.401 | 1.300 |
| C15.0 | SNV | 4000~400 | PLSR | Milk | 0.346 | 1.245 | 0.434 | 1.337 |
| C15.0 | SG | 4000~400 | PLSR | Milk | 0.304 | 1.213 | 0.428 | 1.330 |
| C15.0 | MSC | 4000~400 | RFR | Milk | 0.386 | 1.277 | 0.461 | 1.370 |
| C15.0 | DER1 | 4000~400 | RFR | Milk | 0.441 | 1.339 | 0.486 | 1.402 |
| C15.0 | DER2 | 4000~400 | RFR | Milk | 0.460 | 1.363 | 0.451 | 1.357 |
| C15.0 | SNV | 4000~400 | RFR | Milk | 0.377 | 1.267 | 0.435 | 1.336 |
| C15.0 | SG | 4000~400 | RFR | Milk | 0.424 | 1.319 | 0.438 | 1.341 |
| C15.0 | MSC | 4000~400 | RidgeR | Milk | 0.439 | 1.331 | 0.436 | 1.323 |
| C15.0 | DER1 | 4000~400 | RidgeR | Milk | 0.550 | 1.471 | 0.460 | 1.352 |
| C15.0 | DER2 | 4000~400 | RidgeR | Milk | 0.679 | 1.678 | 0.466 | 1.355 |
| C15.0 | SNV | 4000~400 | RidgeR | Milk | 0.467 | 1.361 | 0.465 | 1.357 |
| C15.0 | SG | 4000~400 | RidgeR | Milk | 0.440 | 1.332 | 0.412 | 1.295 |
| C16.0 | MSC | 3017~2823/1805~1734 | LassoR | Fat | 0.241 | 1.149 | 0.178 | 1.086 |
| C16.0 | DER1 | 3017~2823/1805~1734 | LassoR | Fat | 0.286 | 1.183 | 0.172 | 1.082 |
| C16.0 | DER2 | 3017~2823/1805~1734 | LassoR | Fat | 0.272 | 1.171 | 0.163 | 1.074 |
| C16.0 | SNV | 3017~2823/1805~1734 | LassoR | Fat | 0.249 | 1.155 | 0.197 | 1.101 |
| C16.0 | SG | 3017~2823/1805~1734 | LassoR | Fat | 0.278 | 1.173 | 0.193 | 1.098 |
| C16.0 | MSC | 3017~2823/1805~1734 | RidgeR | Fat | 0.163 | 1.091 | 0.108 | 1.053 |
| C16.0 | DER1 | 3017~2823/1805~1734 | RidgeR | Fat | 0.233 | 1.137 | 0.160 | 1.079 |
| C16.0 | DER2 | 3017~2823/1805~1734 | RidgeR | Fat | 0.223 | 1.130 | 0.146 | 1.069 |
| C16.0 | SNV | 3017~2823/1805~1734 | RidgeR | Fat | 0.150 | 1.084 | 0.107 | 1.053 |
| C16.0 | SG | 3017~2823/1805~1734 | RidgeR | Fat | 0.101 | 1.056 | 0.115 | 1.056 |
| C16.0 | MSC | 3017~2823/1805~1734 | RFR | Fat | 0.074 | 1.017 | 0.049 | 0.965 |
| C16.0 | DER1 | 3017~2823/1805~1734 | RFR | Fat | 0.051 | 1.004 | 0.105 | 1.023 |
| C16.0 | DER2 | 3017~2823/1805~1734 | RFR | Fat | 0.103 | 1.055 | 0.064 | 0.978 |
| C16.0 | SNV | 3017~2823/1805~1734 | RFR | Fat | 0.069 | 1.009 | 0.033 | 0.956 |
| C16.0 | SG | 3017~2823/1805~1734 | RFR | Fat | 0.012 | 0.947 | 0.037 | 0.972 |
| C16.0 | MSC | 3017~2823/1805~1734 | PLSR | Fat | 0.159 | 1.097 | 0.154 | 1.094 |
| C16.0 | DER1 | 3017~2823/1805~1734 | PLSR | Fat | 0.148 | 1.091 | 0.090 | 1.055 |
| C16.0 | DER2 | 3017~2823/1805~1734 | PLSR | Fat | 0.134 | 1.079 | 0.083 | 1.051 |
| C16.0 | SNV | 3017~2823/1805~1734 | PLSR | Fat | 0.152 | 1.094 | 0.097 | 1.059 |
| C16.0 | SG | 3017~2823/1805~1734 | PLSR | Fat | 0.185 | 1.114 | 0.131 | 1.079 |
| C16.0 | MSC | 3017~2823/1805~1734 | LassoR | Milk | 0.722 | 1.902 | 0.640 | 1.626 |
| C16.0 | DER1 | 3017~2823/1805~1734 | LassoR | Milk | 0.738 | 1.957 | 0.604 | 1.484 |
| C16.0 | DER2 | 3017~2823/1805~1734 | LassoR | Milk | 0.741 | 1.967 | 0.632 | 1.560 |
| C16.0 | SNV | 3017~2823/1805~1734 | LassoR | Milk | 0.723 | 1.902 | 0.644 | 1.633 |
| C16.0 | SG | 3017~2823/1805~1734 | LassoR | Milk | 0.688 | 1.794 | 0.639 | 1.586 |
| C16.0 | MSC | 3017~2823/1805~1734 | PLSR | Milk | 0.672 | 1.755 | 0.626 | 1.644 |
| C16.0 | DER1 | 3017~2823/1805~1734 | PLSR | Milk | 0.688 | 1.797 | 0.564 | 1.524 |
| C16.0 | DER2 | 3017~2823/1805~1734 | PLSR | Milk | 0.684 | 1.785 | 0.582 | 1.557 |
| C16.0 | SNV | 3017~2823/1805~1734 | PLSR | Milk | 0.677 | 1.763 | 0.642 | 1.683 |
| C16.0 | SG | 3017~2823/1805~1734 | PLSR | Milk | 0.689 | 1.800 | 0.549 | 1.498 |
| C16.0 | MSC | 3017~2823/1805~1734 | RFR | Milk | 0.649 | 1.690 | 0.717 | 1.860 |
| C16.0 | DER1 | 3017~2823/1805~1734 | RFR | Milk | 0.655 | 1.705 | 0.735 | 1.931 |
| C16.0 | DER2 | 3017~2823/1805~1734 | RFR | Milk | 0.644 | 1.678 | 0.737 | 1.943 |
| C16.0 | SNV | 3017~2823/1805~1734 | RFR | Milk | 0.644 | 1.679 | 0.721 | 1.866 |
| C16.0 | SG | 3017~2823/1805~1734 | RFR | Milk | 0.637 | 1.661 | 0.752 | 1.975 |
| C16.0 | MSC | 3017~2823/1805~1734 | RidgeR | Milk | 0.674 | 1.755 | 0.703 | 1.803 |
| C16.0 | DER1 | 3017~2823/1805~1734 | RidgeR | Milk | 0.706 | 1.848 | 0.639 | 1.591 |

| | | | | | | | | |
|---|---|---|---|---|---|---|---|---|
| C16.0 | DER2 | 3017~2823/1805~1734 | RidgeR | Milk | 0.711 | 1.864 | 0.640 | 1.589 |
| C16.0 | SNV | 3017~2823/1805~1734 | RidgeR | Milk | 0.678 | 1.766 | 0.691 | 1.761 |
| C16.0 | SG | 3017~2823/1805~1734 | RidgeR | Milk | 0.672 | 1.750 | 0.642 | 1.606 |
| C16.0 | MSC | 4000~400 | RidgeR | Fat | 0.157 | 1.079 | 0.157 | 1.073 |
| C16.0 | DER1 | 4000~400 | RidgeR | Fat | 0.533 | 1.330 | 0.210 | 1.116 |
| C16.0 | DER2 | 4000~400 | RidgeR | Fat | 0.552 | 1.330 | 0.218 | 1.117 |
| C16.0 | SNV | 4000~400 | RidgeR | Fat | 0.152 | 1.075 | 0.151 | 1.067 |
| C16.0 | SG | 4000~400 | RidgeR | Fat | 0.143 | 1.073 | 0.150 | 1.069 |
| C16.0 | MSC | 4000~400 | RFR | Fat | 0.127 | 1.069 | 0.099 | 1.036 |
| C16.0 | DER1 | 4000~400 | RFR | Fat | 0.157 | 1.088 | 0.094 | 1.036 |
| C16.0 | DER2 | 4000~400 | RFR | Fat | 0.127 | 1.072 | 0.127 | 1.063 |
| C16.0 | SNV | 4000~400 | RFR | Fat | 0.118 | 1.063 | 0.087 | 1.015 |
| C16.0 | SG | 4000~400 | RFR | Fat | 0.126 | 1.067 | 0.092 | 1.036 |
| C16.0 | MSC | 4000~400 | PLSR | Fat | 0.047 | 1.028 | 0.125 | 1.076 |
| C16.0 | DER1 | 4000~400 | PLSR | Fat | 0.032 | 1.039 | 0.039 | 1.026 |
| C16.0 | DER2 | 4000~400 | PLSR | Fat | 0.080 | 1.052 | 0.207 | 1.130 |
| C16.0 | SNV | 4000~400 | PLSR | Fat | 0.044 | 1.026 | 0.120 | 1.073 |
| C16.0 | SG | 4000~400 | PLSR | Fat | 0.035 | 1.022 | 0.152 | 1.093 |
| C16.0 | MSC | 4000~400 | LassoR | Fat | 0.724 | 1.781 | 0.097 | 0.954 |
| C16.0 | DER1 | 4000~400 | LassoR | Fat | 0.924 | 3.261 | 0.132 | 0.946 |
| C16.0 | DER2 | 4000~400 | LassoR | Fat | 0.990 | 8.348 | 0.185 | 0.975 |
| C16.0 | SNV | 4000~400 | LassoR | Fat | 0.727 | 1.799 | 0.089 | 0.944 |
| C16.0 | SG | 4000~400 | LassoR | Fat | 0.628 | 1.573 | 0.148 | 1.028 |
| C16.0 | MSC | 4000~400 | LassoR | Milk | 0.933 | 3.830 | 0.537 | 1.263 |
| C16.0 | DER1 | 4000~400 | LassoR | Milk | 0.984 | 7.597 | 0.616 | 1.363 |
| C16.0 | DER2 | 4000~400 | LassoR | Milk | 0.997 | 16.683 | 0.595 | 1.437 |
| C16.0 | SNV | 4000~400 | LassoR | Milk | 0.910 | 3.299 | 0.586 | 1.419 |
| C16.0 | SG | 4000~400 | LassoR | Milk | 0.909 | 3.301 | 0.557 | 1.327 |
| C16.0 | MSC | 4000~400 | PLSR | Milk | 0.654 | 1.705 | 0.636 | 1.667 |
| C16.0 | DER1 | 4000~400 | PLSR | Milk | 0.669 | 1.743 | 0.618 | 1.627 |
| C16.0 | DER2 | 4000~400 | PLSR | Milk | 0.673 | 1.764 | 0.630 | 1.655 |
| C16.0 | SNV | 4000~400 | PLSR | Milk | 0.655 | 1.706 | 0.640 | 1.676 |
| C16.0 | SG | 4000~400 | PLSR | Milk | 0.664 | 1.732 | 0.603 | 1.597 |
| C16.0 | MSC | 4000~400 | RFR | Milk | 0.649 | 1.691 | 0.716 | 1.879 |
| C16.0 | DER1 | 4000~400 | RFR | Milk | 0.674 | 1.755 | 0.743 | 1.977 |
| C16.0 | DER2 | 4000~400 | RFR | Milk | 0.668 | 1.739 | 0.738 | 1.951 |
| C16.0 | SNV | 4000~400 | RFR | Milk | 0.659 | 1.715 | 0.732 | 1.939 |
| C16.0 | SG | 4000~400 | RFR | Milk | 0.669 | 1.741 | 0.748 | 1.999 |
| C16.0 | MSC | 4000~400 | RidgeR | Milk | 0.696 | 1.811 | 0.671 | 1.731 |
| C16.0 | DER1 | 4000~400 | RidgeR | Milk | 0.750 | 1.987 | 0.680 | 1.732 |
| C16.0 | DER2 | 4000~400 | RidgeR | Milk | 0.794 | 2.164 | 0.681 | 1.743 |
| C16.0 | SNV | 4000~400 | RidgeR | Milk | 0.699 | 1.819 | 0.684 | 1.770 |
| C16.0 | SG | 4000~400 | RidgeR | Milk | 0.705 | 1.842 | 0.654 | 1.662 |
| C16.1 | MSC | 3017~2823/1805~1734 | LassoR | Fat | 0.384 | 1.274 | 0.553 | 1.496 |
| C16.1 | DER1 | 3017~2823/1805~1734 | LassoR | Fat | 0.460 | 1.354 | 0.455 | 1.361 |
| C16.1 | DER2 | 3017~2823/1805~1734 | LassoR | Fat | 0.458 | 1.356 | 0.376 | 1.261 |
| C16.1 | SNV | 3017~2823/1805~1734 | LassoR | Fat | 0.392 | 1.282 | 0.536 | 1.468 |
| C16.1 | SG | 3017~2823/1805~1734 | LassoR | Fat | 0.331 | 1.221 | 0.482 | 1.397 |
| C16.1 | MSC | 3017~2823/1805~1734 | RidgeR | Fat | 0.254 | 1.150 | 0.363 | 1.224 |
| C16.1 | DER1 | 3017~2823/1805~1734 | RidgeR | Fat | 0.214 | 1.123 | 0.313 | 1.178 |

| | | | | | | | | |
|---|---|---|---|---|---|---|---|---|
| C16.1 | DER2 | 3017~2823/1805~1734 | RidgeR | Fat | 0.273 | 1.165 | 0.355 | 1.211 |
| C16.1 | SNV | 3017~2823/1805~1734 | RidgeR | Fat | 0.241 | 1.146 | 0.239 | 1.150 |
| C16.1 | SG | 3017~2823/1805~1734 | RidgeR | Fat | 0.136 | 1.076 | 0.193 | 1.106 |
| C16.1 | MSC | 3017~2823/1805~1734 | RFR | Fat | 0.063 | 1.003 | 0.077 | 1.037 |
| C16.1 | DER1 | 3017~2823/1805~1734 | RFR | Fat | 0.032 | 0.985 | 0.060 | 1.031 |
| C16.1 | DER2 | 3017~2823/1805~1734 | RFR | Fat | 0.092 | 1.041 | 0.029 | 0.985 |
| C16.1 | SNV | 3017~2823/1805~1734 | RFR | Fat | 0.045 | 0.989 | 0.082 | 1.036 |
| C16.1 | SG | 3017~2823/1805~1734 | RFR | Fat | 0.023 | 0.967 | 0.007 | 0.958 |
| C16.1 | MSC | 3017~2823/1805~1734 | PLSR | Fat | 0.275 | 1.185 | 0.468 | 1.380 |
| C16.1 | DER1 | 3017~2823/1805~1734 | PLSR | Fat | 0.172 | 1.113 | 0.315 | 1.216 |
| C16.1 | DER2 | 3017~2823/1805~1734 | PLSR | Fat | 0.110 | 1.070 | 0.357 | 1.255 |
| C16.1 | SNV | 3017~2823/1805~1734 | PLSR | Fat | 0.268 | 1.182 | 0.424 | 1.326 |
| C16.1 | SG | 3017~2823/1805~1734 | PLSR | Fat | 0.268 | 1.181 | 0.359 | 1.257 |
| C16.1 | MSC | 3017~2823/1805~1734 | LassoR | Milk | 0.499 | 1.413 | 0.587 | 1.560 |
| C16.1 | DER1 | 3017~2823/1805~1734 | LassoR | Milk | 0.500 | 1.413 | 0.533 | 1.465 |
| C16.1 | DER2 | 3017~2823/1805~1734 | LassoR | Milk | 0.501 | 1.415 | 0.520 | 1.447 |
| C16.1 | SNV | 3017~2823/1805~1734 | LassoR | Milk | 0.537 | 1.470 | 0.624 | 1.638 |
| C16.1 | SG | 3017~2823/1805~1734 | LassoR | Milk | 0.478 | 1.384 | 0.520 | 1.448 |
| C16.1 | MSC | 3017~2823/1805~1734 | PLSR | Milk | 0.443 | 1.347 | 0.577 | 1.547 |
| C16.1 | DER1 | 3017~2823/1805~1734 | PLSR | Milk | 0.339 | 1.247 | 0.483 | 1.399 |
| C16.1 | DER2 | 3017~2823/1805~1734 | PLSR | Milk | 0.302 | 1.211 | 0.517 | 1.448 |
| C16.1 | SNV | 3017~2823/1805~1734 | PLSR | Milk | 0.441 | 1.345 | 0.569 | 1.533 |
| C16.1 | SG | 3017~2823/1805~1734 | PLSR | Milk | 0.392 | 1.290 | 0.514 | 1.443 |
| C16.1 | MSC | 3017~2823/1805~1734 | RFR | Milk | 0.316 | 1.208 | 0.409 | 1.306 |
| C16.1 | DER1 | 3017~2823/1805~1734 | RFR | Milk | 0.305 | 1.200 | 0.311 | 1.207 |
| C16.1 | DER2 | 3017~2823/1805~1734 | RFR | Milk | 0.314 | 1.209 | 0.412 | 1.311 |
| C16.1 | SNV | 3017~2823/1805~1734 | RFR | Milk | 0.340 | 1.228 | 0.439 | 1.343 |
| C16.1 | SG | 3017~2823/1805~1734 | RFR | Milk | 0.259 | 1.156 | 0.318 | 1.216 |
| C16.1 | MSC | 3017~2823/1805~1734 | RidgeR | Milk | 0.407 | 1.294 | 0.511 | 1.419 |
| C16.1 | DER1 | 3017~2823/1805~1734 | RidgeR | Milk | 0.399 | 1.288 | 0.518 | 1.398 |
| C16.1 | DER2 | 3017~2823/1805~1734 | RidgeR | Milk | 0.425 | 1.318 | 0.503 | 1.400 |
| C16.1 | SNV | 3017~2823/1805~1734 | RidgeR | Milk | 0.388 | 1.278 | 0.510 | 1.411 |
| C16.1 | SG | 3017~2823/1805~1734 | RidgeR | Milk | 0.326 | 1.219 | 0.473 | 1.330 |
| C16.1 | MSC | 4000~400 | RidgeR | Fat | 0.331 | 1.177 | 0.140 | 1.080 |
| C16.1 | DER1 | 4000~400 | RidgeR | Fat | 0.428 | 1.222 | 0.103 | 1.061 |
| C16.1 | DER2 | 4000~400 | RidgeR | Fat | 0.741 | 1.553 | 0.118 | 1.071 |
| C16.1 | SNV | 4000~400 | RidgeR | Fat | 0.337 | 1.183 | 0.116 | 1.069 |
| C16.1 | SG | 4000~400 | RidgeR | Fat | 0.270 | 1.143 | 0.081 | 1.049 |
| C16.1 | MSC | 4000~400 | RFR | Fat | 0.120 | 1.062 | 0.092 | 1.053 |
| C16.1 | DER1 | 4000~400 | RFR | Fat | 0.097 | 1.051 | 0.047 | 1.017 |
| C16.1 | DER2 | 4000~400 | RFR | Fat | 0.097 | 1.053 | 0.035 | 1.016 |
| C16.1 | SNV | 4000~400 | RFR | Fat | 0.147 | 1.081 | 0.063 | 1.028 |
| C16.1 | SG | 4000~400 | RFR | Fat | 0.088 | 1.038 | 0.067 | 1.037 |
| C16.1 | MSC | 4000~400 | PLSR | Fat | 0.058 | 1.033 | 0.018 | 1.015 |
| C16.1 | DER1 | 4000~400 | PLSR | Fat | 0.039 | 1.023 | 0.015 | 1.014 |
| C16.1 | DER2 | 4000~400 | PLSR | Fat | 0.041 | 1.051 | 0.120 | 1.073 |
| C16.1 | SNV | 4000~400 | PLSR | Fat | 0.109 | 1.076 | 0.115 | 1.069 |
| C16.1 | SG | 4000~400 | PLSR | Fat | 0.046 | 1.026 | 0.011 | 1.012 |
| C16.1 | MSC | 4000~400 | LassoR | Fat | 0.763 | 1.966 | 0.182 | 1.059 |
| C16.1 | DER1 | 4000~400 | LassoR | Fat | 0.929 | 3.218 | 0.108 | 0.955 |

| | | | | | | | | |
|---|---|---|---|---|---|---|---|---|
| C16.1 | DER2 | 4000~400 | LassoR | Fat | 0.998 | 16.482 | 0.120 | 0.941 |
| C16.1 | SNV | 4000~400 | LassoR | Fat | 0.756 | 1.941 | 0.139 | 1.007 |
| C16.1 | SG | 4000~400 | LassoR | Fat | 0.642 | 1.617 | 0.225 | 1.124 |
| C16.1 | MSC | 4000~400 | LassoR | Milk | 0.847 | 2.494 | 0.339 | 1.138 |
| C16.1 | DER1 | 4000~400 | LassoR | Milk | 0.977 | 6.074 | 0.241 | 1.013 |
| C16.1 | DER2 | 4000~400 | LassoR | Milk | 1.000 | 39.455 | 0.229 | 1.007 |
| C16.1 | SNV | 4000~400 | LassoR | Milk | 0.828 | 2.349 | 0.378 | 1.206 |
| C16.1 | SG | 4000~400 | LassoR | Milk | 0.789 | 2.141 | 0.383 | 1.232 |
| C16.1 | MSC | 4000~400 | PLSR | Milk | 0.251 | 1.180 | 0.252 | 1.164 |
| C16.1 | DER1 | 4000~400 | PLSR | Milk | 0.231 | 1.171 | 0.223 | 1.142 |
| C16.1 | DER2 | 4000~400 | PLSR | Milk | 0.345 | 1.282 | 0.291 | 1.195 |
| C16.1 | SNV | 4000~400 | PLSR | Milk | 0.245 | 1.168 | 0.223 | 1.142 |
| C16.1 | SG | 4000~400 | PLSR | Milk | 0.210 | 1.148 | 0.258 | 1.168 |
| C16.1 | MSC | 4000~400 | RFR | Milk | 0.377 | 1.269 | 0.325 | 1.221 |
| C16.1 | DER1 | 4000~400 | RFR | Milk | 0.344 | 1.237 | 0.365 | 1.261 |
| C16.1 | DER2 | 4000~400 | RFR | Milk | 0.343 | 1.235 | 0.343 | 1.237 |
| C16.1 | SNV | 4000~400 | RFR | Milk | 0.378 | 1.270 | 0.377 | 1.274 |
| C16.1 | SG | 4000~400 | RFR | Milk | 0.325 | 1.219 | 0.342 | 1.232 |
| C16.1 | MSC | 4000~400 | RidgeR | Milk | 0.412 | 1.293 | 0.446 | 1.322 |
| C16.1 | DER1 | 4000~400 | RidgeR | Milk | 0.520 | 1.404 | 0.427 | 1.297 |
| C16.1 | DER2 | 4000~400 | RidgeR | Milk | 0.669 | 1.600 | 0.472 | 1.330 |
| C16.1 | SNV | 4000~400 | RidgeR | Milk | 0.414 | 1.296 | 0.443 | 1.319 |
| C16.1 | SG | 4000~400 | RidgeR | Milk | 0.374 | 1.258 | 0.444 | 1.306 |
| C17.0 | MSC | 3017~2823/1805~1734 | LassoR | Fat | 0.517 | 1.441 | 0.518 | 1.427 |
| C17.0 | DER1 | 3017~2823/1805~1734 | LassoR | Fat | 0.606 | 1.596 | 0.451 | 1.356 |
| C17.0 | DER2 | 3017~2823/1805~1734 | LassoR | Fat | 0.498 | 1.413 | 0.459 | 1.356 |
| C17.0 | SNV | 3017~2823/1805~1734 | LassoR | Fat | 0.530 | 1.460 | 0.527 | 1.437 |
| C17.0 | SG | 3017~2823/1805~1734 | LassoR | Fat | 0.505 | 1.422 | 0.495 | 1.401 |
| C17.0 | MSC | 3017~2823/1805~1734 | RidgeR | Fat | 0.399 | 1.293 | 0.496 | 1.384 |
| C17.0 | DER1 | 3017~2823/1805~1734 | RidgeR | Fat | 0.439 | 1.332 | 0.463 | 1.344 |
| C17.0 | DER2 | 3017~2823/1805~1734 | RidgeR | Fat | 0.451 | 1.348 | 0.462 | 1.344 |
| C17.0 | SNV | 3017~2823/1805~1734 | RidgeR | Fat | 0.406 | 1.300 | 0.505 | 1.395 |
| C17.0 | SG | 3017~2823/1805~1734 | RidgeR | Fat | 0.395 | 1.288 | 0.439 | 1.326 |
| C17.0 | MSC | 3017~2823/1805~1734 | RFR | Fat | 0.317 | 1.208 | 0.475 | 1.383 |
| C17.0 | DER1 | 3017~2823/1805~1734 | RFR | Fat | 0.290 | 1.183 | 0.385 | 1.262 |
| C17.0 | DER2 | 3017~2823/1805~1734 | RFR | Fat | 0.317 | 1.211 | 0.458 | 1.327 |
| C17.0 | SNV | 3017~2823/1805~1734 | RFR | Fat | 0.309 | 1.198 | 0.489 | 1.392 |
| C17.0 | SG | 3017~2823/1805~1734 | RFR | Fat | 0.253 | 1.151 | 0.395 | 1.284 |
| C17.0 | MSC | 3017~2823/1805~1734 | PLSR | Fat | 0.398 | 1.319 | 0.585 | 1.562 |
| C17.0 | DER1 | 3017~2823/1805~1734 | PLSR | Fat | 0.395 | 1.298 | 0.418 | 1.319 |
| C17.0 | DER2 | 3017~2823/1805~1734 | PLSR | Fat | 0.382 | 1.278 | 0.456 | 1.365 |
| C17.0 | SNV | 3017~2823/1805~1734 | PLSR | Fat | 0.420 | 1.326 | 0.565 | 1.527 |
| C17.0 | SG | 3017~2823/1805~1734 | PLSR | Fat | 0.438 | 1.342 | 0.503 | 1.428 |
| C17.0 | MSC | 3017~2823/1805~1734 | LassoR | Milk | 0.617 | 1.619 | 0.704 | 1.787 |
| C17.0 | DER1 | 3017~2823/1805~1734 | LassoR | Milk | 0.734 | 1.942 | 0.685 | 1.770 |
| C17.0 | DER2 | 3017~2823/1805~1734 | LassoR | Milk | 0.741 | 1.967 | 0.690 | 1.776 |
| C17.0 | SNV | 3017~2823/1805~1734 | LassoR | Milk | 0.677 | 1.761 | 0.692 | 1.759 |
| C17.0 | SG | 3017~2823/1805~1734 | LassoR | Milk | 0.652 | 1.699 | 0.732 | 1.887 |
| C17.0 | MSC | 3017~2823/1805~1734 | PLSR | Milk | 0.599 | 1.598 | 0.647 | 1.693 |
| C17.0 | DER1 | 3017~2823/1805~1734 | PLSR | Milk | 0.644 | 1.691 | 0.654 | 1.710 |

| | | | | | | | | |
|---|---|---|---|---|---|---|---|---|
| C17.0 | DER2 | 3017~2823/1805~1734 | PLSR | Milk | 0.614 | 1.623 | 0.660 | 1.726 |
| C17.0 | SNV | 3017~2823/1805~1734 | PLSR | Milk | 0.601 | 1.601 | 0.635 | 1.665 |
| C17.0 | SG | 3017~2823/1805~1734 | PLSR | Milk | 0.639 | 1.680 | 0.681 | 1.781 |
| C17.0 | MSC | 3017~2823/1805~1734 | RFR | Milk | 0.580 | 1.545 | 0.676 | 1.719 |
| C17.0 | DER1 | 3017~2823/1805~1734 | RFR | Milk | 0.593 | 1.572 | 0.648 | 1.648 |
| C17.0 | DER2 | 3017~2823/1805~1734 | RFR | Milk | 0.585 | 1.555 | 0.619 | 1.611 |
| C17.0 | SNV | 3017~2823/1805~1734 | RFR | Milk | 0.578 | 1.542 | 0.654 | 1.657 |
| C17.0 | SG | 3017~2823/1805~1734 | RFR | Milk | 0.569 | 1.525 | 0.638 | 1.616 |
| C17.0 | MSC | 3017~2823/1805~1734 | RidgeR | Milk | 0.597 | 1.579 | 0.683 | 1.722 |
| C17.0 | DER1 | 3017~2823/1805~1734 | RidgeR | Milk | 0.642 | 1.672 | 0.706 | 1.788 |
| C17.0 | DER2 | 3017~2823/1805~1734 | RidgeR | Milk | 0.674 | 1.753 | 0.680 | 1.741 |
| C17.0 | SNV | 3017~2823/1805~1734 | RidgeR | Milk | 0.600 | 1.584 | 0.684 | 1.723 |
| C17.0 | SG | 3017~2823/1805~1734 | RidgeR | Milk | 0.626 | 1.637 | 0.718 | 1.832 |
| C17.0 | MSC | 4000~400 | RidgeR | Fat | 0.461 | 1.353 | 0.437 | 1.308 |
| C17.0 | DER1 | 4000~400 | RidgeR | Fat | 0.548 | 1.465 | 0.541 | 1.428 |
| C17.0 | DER2 | 4000~400 | RidgeR | Fat | 0.551 | 1.455 | 0.494 | 1.352 |
| C17.0 | SNV | 4000~400 | RidgeR | Fat | 0.462 | 1.354 | 0.455 | 1.324 |
| C17.0 | SG | 4000~400 | RidgeR | Fat | 0.454 | 1.343 | 0.431 | 1.308 |
| C17.0 | MSC | 4000~400 | RFR | Fat | 0.334 | 1.227 | 0.420 | 1.305 |
| C17.0 | DER1 | 4000~400 | RFR | Fat | 0.348 | 1.241 | 0.490 | 1.355 |
| C17.0 | DER2 | 4000~400 | RFR | Fat | 0.363 | 1.254 | 0.452 | 1.306 |
| C17.0 | SNV | 4000~400 | RFR | Fat | 0.320 | 1.215 | 0.454 | 1.341 |
| C17.0 | SG | 4000~400 | RFR | Fat | 0.328 | 1.222 | 0.510 | 1.377 |
| C17.0 | MSC | 4000~400 | PLSR | Fat | 0.340 | 1.235 | 0.397 | 1.296 |
| C17.0 | DER1 | 4000~400 | PLSR | Fat | 0.360 | 1.255 | 0.515 | 1.445 |
| C17.0 | DER2 | 4000~400 | PLSR | Fat | 0.353 | 1.247 | 0.398 | 1.297 |
| C17.0 | SNV | 4000~400 | PLSR | Fat | 0.339 | 1.234 | 0.396 | 1.295 |
| C17.0 | SG | 4000~400 | PLSR | Fat | 0.342 | 1.238 | 0.418 | 1.319 |
| C17.0 | MSC | 4000~400 | LassoR | Fat | 0.784 | 2.109 | 0.413 | 1.283 |
| C17.0 | DER1 | 4000~400 | LassoR | Fat | 0.969 | 5.266 | 0.438 | 1.265 |
| C17.0 | DER2 | 4000~400 | LassoR | Fat | 0.996 | 14.516 | 0.439 | 1.270 |
| C17.0 | SNV | 4000~400 | LassoR | Fat | 0.780 | 2.090 | 0.416 | 1.291 |
| C17.0 | SG | 4000~400 | LassoR | Fat | 0.741 | 1.943 | 0.407 | 1.280 |
| C17.0 | MSC | 4000~400 | LassoR | Milk | 0.885 | 2.909 | 0.547 | 1.391 |
| C17.0 | DER1 | 4000~400 | LassoR | Milk | 0.970 | 5.613 | 0.581 | 1.457 |
| C17.0 | DER2 | 4000~400 | LassoR | Milk | 0.993 | 10.843 | 0.563 | 1.480 |
| C17.0 | SNV | 4000~400 | LassoR | Milk | 0.884 | 2.909 | 0.564 | 1.403 |
| C17.0 | SG | 4000~400 | LassoR | Milk | 0.862 | 2.679 | 0.598 | 1.504 |
| C17.0 | MSC | 4000~400 | PLSR | Milk | 0.573 | 1.535 | 0.649 | 1.699 |
| C17.0 | DER1 | 4000~400 | PLSR | Milk | 0.587 | 1.565 | 0.675 | 1.766 |
| C17.0 | DER2 | 4000~400 | PLSR | Milk | 0.546 | 1.526 | 0.624 | 1.641 |
| C17.0 | SNV | 4000~400 | PLSR | Milk | 0.575 | 1.540 | 0.651 | 1.704 |
| C17.0 | SG | 4000~400 | PLSR | Milk | 0.581 | 1.552 | 0.684 | 1.790 |
| C17.0 | MSC | 4000~400 | RFR | Milk | 0.571 | 1.529 | 0.664 | 1.687 |
| C17.0 | DER1 | 4000~400 | RFR | Milk | 0.617 | 1.619 | 0.683 | 1.742 |
| C17.0 | DER2 | 4000~400 | RFR | Milk | 0.605 | 1.593 | 0.652 | 1.663 |
| C17.0 | SNV | 4000~400 | RFR | Milk | 0.567 | 1.523 | 0.663 | 1.692 |
| C17.0 | SG | 4000~400 | RFR | Milk | 0.598 | 1.581 | 0.684 | 1.729 |
| C17.0 | MSC | 4000~400 | RidgeR | Milk | 0.621 | 1.618 | 0.663 | 1.683 |
| C17.0 | DER1 | 4000~400 | RidgeR | Milk | 0.711 | 1.850 | 0.714 | 1.823 |

| | | | | | | | | |
|---|---|---|---|---|---|---|---|---|
| C17.0 | DER2 | 4000~400 | RidgeR | Milk | 0.756 | 1.971 | 0.690 | 1.715 |
| C17.0 | SNV | 4000~400 | RidgeR | Milk | 0.622 | 1.621 | 0.668 | 1.692 |
| C17.0 | SG | 4000~400 | RidgeR | Milk | 0.633 | 1.650 | 0.697 | 1.764 |
| C18.0 | MSC | 3017~2823/1805~1734 | LassoR | Fat | 0.587 | 1.557 | 0.540 | 1.482 |
| C18.0 | DER1 | 3017~2823/1805~1734 | LassoR | Fat | 0.609 | 1.599 | 0.517 | 1.444 |
| C18.0 | DER2 | 3017~2823/1805~1734 | LassoR | Fat | 0.661 | 1.720 | 0.522 | 1.435 |
| C18.0 | SNV | 3017~2823/1805~1734 | LassoR | Fat | 0.600 | 1.583 | 0.545 | 1.490 |
| C18.0 | SG | 3017~2823/1805~1734 | LassoR | Fat | 0.595 | 1.575 | 0.533 | 1.459 |
| C18.0 | MSC | 3017~2823/1805~1734 | RidgeR | Fat | 0.468 | 1.371 | 0.398 | 1.288 |
| C18.0 | DER1 | 3017~2823/1805~1734 | RidgeR | Fat | 0.529 | 1.445 | 0.424 | 1.325 |
| C18.0 | DER2 | 3017~2823/1805~1734 | RidgeR | Fat | 0.564 | 1.509 | 0.461 | 1.369 |
| C18.0 | SNV | 3017~2823/1805~1734 | RidgeR | Fat | 0.463 | 1.366 | 0.388 | 1.275 |
| C18.0 | SG | 3017~2823/1805~1734 | RidgeR | Fat | 0.465 | 1.368 | 0.378 | 1.256 |
| C18.0 | MSC | 3017~2823/1805~1734 | RFR | Fat | 0.330 | 1.217 | 0.289 | 1.182 |
| C18.0 | DER1 | 3017~2823/1805~1734 | RFR | Fat | 0.246 | 1.154 | 0.188 | 1.114 |
| C18.0 | DER2 | 3017~2823/1805~1734 | RFR | Fat | 0.332 | 1.226 | 0.390 | 1.277 |
| C18.0 | SNV | 3017~2823/1805~1734 | RFR | Fat | 0.325 | 1.217 | 0.342 | 1.229 |
| C18.0 | SG | 3017~2823/1805~1734 | RFR | Fat | 0.217 | 1.123 | 0.342 | 1.236 |
| C18.0 | MSC | 3017~2823/1805~1734 | PLSR | Fat | 0.487 | 1.405 | 0.518 | 1.449 |
| C18.0 | DER1 | 3017~2823/1805~1734 | PLSR | Fat | 0.507 | 1.432 | 0.514 | 1.444 |
| C18.0 | DER2 | 3017~2823/1805~1734 | PLSR | Fat | 0.487 | 1.424 | 0.511 | 1.440 |
| C18.0 | SNV | 3017~2823/1805~1734 | PLSR | Fat | 0.485 | 1.403 | 0.526 | 1.462 |
| C18.0 | SG | 3017~2823/1805~1734 | PLSR | Fat | 0.519 | 1.451 | 0.534 | 1.474 |
| C18.0 | MSC | 3017~2823/1805~1734 | LassoR | Milk | 0.740 | 1.965 | 0.624 | 1.641 |
| C18.0 | DER1 | 3017~2823/1805~1734 | LassoR | Milk | 0.783 | 2.149 | 0.702 | 1.839 |
| C18.0 | DER2 | 3017~2823/1805~1734 | LassoR | Milk | 0.788 | 2.177 | 0.717 | 1.892 |
| C18.0 | SNV | 3017~2823/1805~1734 | LassoR | Milk | 0.727 | 1.917 | 0.651 | 1.700 |
| C18.0 | SG | 3017~2823/1805~1734 | LassoR | Milk | 0.748 | 1.998 | 0.714 | 1.882 |
| C18.0 | MSC | 3017~2823/1805~1734 | PLSR | Milk | 0.664 | 1.742 | 0.576 | 1.546 |
| C18.0 | DER1 | 3017~2823/1805~1734 | PLSR | Milk | 0.719 | 1.898 | 0.719 | 1.898 |
| C18.0 | DER2 | 3017~2823/1805~1734 | PLSR | Milk | 0.696 | 1.830 | 0.707 | 1.859 |
| C18.0 | SNV | 3017~2823/1805~1734 | PLSR | Milk | 0.678 | 1.774 | 0.649 | 1.699 |
| C18.0 | SG | 3017~2823/1805~1734 | PLSR | Milk | 0.706 | 1.858 | 0.731 | 1.941 |
| C18.0 | MSC | 3017~2823/1805~1734 | RFR | Milk | 0.550 | 1.489 | 0.651 | 1.693 |
| C18.0 | DER1 | 3017~2823/1805~1734 | RFR | Milk | 0.560 | 1.510 | 0.627 | 1.636 |
| C18.0 | DER2 | 3017~2823/1805~1734 | RFR | Milk | 0.591 | 1.566 | 0.703 | 1.779 |
| C18.0 | SNV | 3017~2823/1805~1734 | RFR | Milk | 0.546 | 1.483 | 0.670 | 1.743 |
| C18.0 | SG | 3017~2823/1805~1734 | RFR | Milk | 0.594 | 1.572 | 0.700 | 1.805 |
| C18.0 | MSC | 3017~2823/1805~1734 | RidgeR | Milk | 0.654 | 1.704 | 0.711 | 1.825 |
| C18.0 | DER1 | 3017~2823/1805~1734 | RidgeR | Milk | 0.687 | 1.783 | 0.717 | 1.882 |
| C18.0 | DER2 | 3017~2823/1805~1734 | RidgeR | Milk | 0.718 | 1.882 | 0.721 | 1.897 |
| C18.0 | SNV | 3017~2823/1805~1734 | RidgeR | Milk | 0.660 | 1.718 | 0.713 | 1.847 |
| C18.0 | SG | 3017~2823/1805~1734 | RidgeR | Milk | 0.680 | 1.772 | 0.748 | 1.998 |
| C18.0 | MSC | 4000~400 | RidgeR | Fat | 0.491 | 1.390 | 0.348 | 1.243 |
| C18.0 | DER1 | 4000~400 | RidgeR | Fat | 0.636 | 1.607 | 0.445 | 1.349 |
| C18.0 | DER2 | 4000~400 | RidgeR | Fat | 0.722 | 1.778 | 0.469 | 1.381 |
| C18.0 | SNV | 4000~400 | RidgeR | Fat | 0.491 | 1.389 | 0.349 | 1.244 |
| C18.0 | SG | 4000~400 | RidgeR | Fat | 0.501 | 1.405 | 0.370 | 1.263 |
| C18.0 | MSC | 4000~400 | RFR | Fat | 0.381 | 1.273 | 0.349 | 1.245 |
| C18.0 | DER1 | 4000~400 | RFR | Fat | 0.404 | 1.297 | 0.534 | 1.466 |

| | | | | | | | | |
|---|---|---|---|---|---|---|---|---|
| C18.0 | DER2 | 4000~400 | RFR | Fat | 0.394 | 1.281 | 0.436 | 1.330 |
| C18.0 | SNV | 4000~400 | RFR | Fat | 0.368 | 1.259 | 0.377 | 1.275 |
| C18.0 | SG | 4000~400 | RFR | Fat | 0.335 | 1.228 | 0.437 | 1.340 |
| C18.0 | MSC | 4000~400 | PLSR | Fat | 0.386 | 1.281 | 0.324 | 1.224 |
| C18.0 | DER1 | 4000~400 | PLSR | Fat | 0.468 | 1.415 | 0.372 | 1.270 |
| C18.0 | DER2 | 4000~400 | PLSR | Fat | 0.440 | 1.372 | 0.415 | 1.315 |
| C18.0 | SNV | 4000~400 | PLSR | Fat | 0.384 | 1.279 | 0.324 | 1.223 |
| C18.0 | SG | 4000~400 | PLSR | Fat | 0.406 | 1.303 | 0.336 | 1.235 |
| C18.0 | MSC | 4000~400 | LassoR | Fat | 0.868 | 2.703 | 0.269 | 1.044 |
| C18.0 | DER1 | 4000~400 | LassoR | Fat | 0.964 | 5.081 | 0.374 | 1.102 |
| C18.0 | DER2 | 4000~400 | LassoR | Fat | 0.993 | 10.866 | 0.424 | 1.236 |
| C18.0 | SNV | 4000~400 | LassoR | Fat | 0.868 | 2.696 | 0.289 | 1.062 |
| C18.0 | SG | 4000~400 | LassoR | Fat | 0.811 | 2.265 | 0.342 | 1.137 |
| C18.0 | MSC | 4000~400 | LassoR | Milk | 0.880 | 2.857 | 0.608 | 1.606 |
| C18.0 | DER1 | 4000~400 | LassoR | Milk | 0.981 | 6.997 | 0.576 | 1.510 |
| C18.0 | DER2 | 4000~400 | LassoR | Milk | 0.999 | 25.552 | 0.568 | 1.505 |
| C18.0 | SNV | 4000~400 | LassoR | Milk | 0.875 | 2.793 | 0.623 | 1.636 |
| C18.0 | SG | 4000~400 | LassoR | Milk | 0.858 | 2.639 | 0.719 | 1.899 |
| C18.0 | MSC | 4000~400 | PLSR | Milk | 0.610 | 1.606 | 0.699 | 1.836 |
| C18.0 | DER1 | 4000~400 | PLSR | Milk | 0.659 | 1.720 | 0.765 | 2.076 |
| C18.0 | DER2 | 4000~400 | PLSR | Milk | 0.655 | 1.750 | 0.680 | 1.780 |
| C18.0 | SNV | 4000~400 | PLSR | Milk | 0.610 | 1.606 | 0.702 | 1.844 |
| C18.0 | SG | 4000~400 | PLSR | Milk | 0.632 | 1.654 | 0.722 | 1.910 |
| C18.0 | MSC | 4000~400 | RFR | Milk | 0.583 | 1.551 | 0.735 | 1.898 |
| C18.0 | DER1 | 4000~400 | RFR | Milk | 0.669 | 1.735 | 0.763 | 2.014 |
| C18.0 | DER2 | 4000~400 | RFR | Milk | 0.620 | 1.617 | 0.736 | 1.909 |
| C18.0 | SNV | 4000~400 | RFR | Milk | 0.577 | 1.541 | 0.690 | 1.769 |
| C18.0 | SG | 4000~400 | RFR | Milk | 0.602 | 1.587 | 0.735 | 1.909 |
| C18.0 | MSC | 4000~400 | RidgeR | Milk | 0.662 | 1.717 | 0.712 | 1.838 |
| C18.0 | DER1 | 4000~400 | RidgeR | Milk | 0.745 | 1.968 | 0.754 | 2.017 |
| C18.0 | DER2 | 4000~400 | RidgeR | Milk | 0.789 | 2.104 | 0.718 | 1.876 |
| C18.0 | SNV | 4000~400 | RidgeR | Milk | 0.663 | 1.720 | 0.715 | 1.845 |
| C18.0 | SG | 4000~400 | RidgeR | Milk | 0.679 | 1.765 | 0.734 | 1.940 |
| C18.1n9 | MSC | 3017~2823/1805~1734 | LassoR | Fat | 0.524 | 1.451 | 0.341 | 1.197 |
| C18.1n9 | DER1 | 3017~2823/1805~1734 | LassoR | Fat | 0.560 | 1.507 | 0.328 | 1.158 |
| C18.1n9 | DER2 | 3017~2823/1805~1734 | LassoR | Fat | 0.568 | 1.522 | 0.310 | 1.137 |
| C18.1n9 | SNV | 3017~2823/1805~1734 | LassoR | Fat | 0.529 | 1.458 | 0.340 | 1.194 |
| C18.1n9 | SG | 3017~2823/1805~1734 | LassoR | Fat | 0.467 | 1.371 | 0.320 | 1.179 |
| C18.1n9 | MSC | 3017~2823/1805~1734 | RidgeR | Fat | 0.381 | 1.271 | 0.226 | 1.111 |
| C18.1n9 | DER1 | 3017~2823/1805~1734 | RidgeR | Fat | 0.449 | 1.343 | 0.273 | 1.148 |
| C18.1n9 | DER2 | 3017~2823/1805~1734 | RidgeR | Fat | 0.505 | 1.419 | 0.305 | 1.157 |
| C18.1n9 | SNV | 3017~2823/1805~1734 | RidgeR | Fat | 0.377 | 1.267 | 0.236 | 1.115 |
| C18.1n9 | SG | 3017~2823/1805~1734 | RidgeR | Fat | 0.341 | 1.232 | 0.186 | 1.064 |
| C18.1n9 | MSC | 3017~2823/1805~1734 | RFR | Fat | 0.197 | 1.106 | 0.153 | 1.001 |
| C18.1n9 | DER1 | 3017~2823/1805~1734 | RFR | Fat | 0.164 | 1.086 | 0.119 | 1.048 |
| C18.1n9 | DER2 | 3017~2823/1805~1734 | RFR | Fat | 0.203 | 1.111 | 0.122 | 1.046 |
| C18.1n9 | SNV | 3017~2823/1805~1734 | RFR | Fat | 0.200 | 1.108 | 0.152 | 1.051 |
| C18.1n9 | SG | 3017~2823/1805~1734 | RFR | Fat | 0.198 | 1.109 | 0.116 | 1.013 |
| C18.1n9 | MSC | 3017~2823/1805~1734 | PLSR | Fat | 0.438 | 1.348 | 0.252 | 1.164 |
| C18.1n9 | DER1 | 3017~2823/1805~1734 | PLSR | Fat | 0.417 | 1.325 | 0.185 | 1.115 |

| | | | | | | | | |
|---|---|---|---|---|---|---|---|---|
| C18.1n9 | DER2 | 3017~2823/1805~1734 | PLSR | Fat | 0.407 | 1.319 | 0.205 | 1.128 |
| C18.1n9 | SNV | 3017~2823/1805~1734 | PLSR | Fat | 0.434 | 1.343 | 0.231 | 1.147 |
| C18.1n9 | SG | 3017~2823/1805~1734 | PLSR | Fat | 0.402 | 1.308 | 0.258 | 1.168 |
| C18.1n9 | MSC | 3017~2823/1805~1734 | LassoR | Milk | 0.620 | 1.624 | 0.746 | 1.896 |
| C18.1n9 | DER1 | 3017~2823/1805~1734 | LassoR | Milk | 0.626 | 1.637 | 0.752 | 1.952 |
| C18.1n9 | DER2 | 3017~2823/1805~1734 | LassoR | Milk | 0.687 | 1.790 | 0.745 | 1.923 |
| C18.1n9 | SNV | 3017~2823/1805~1734 | LassoR | Milk | 0.621 | 1.627 | 0.748 | 1.908 |
| C18.1n9 | SG | 3017~2823/1805~1734 | LassoR | Milk | 0.598 | 1.580 | 0.770 | 2.000 |
| C18.1n9 | MSC | 3017~2823/1805~1734 | PLSR | Milk | 0.561 | 1.526 | 0.642 | 1.683 |
| C18.1n9 | DER1 | 3017~2823/1805~1734 | PLSR | Milk | 0.551 | 1.500 | 0.754 | 2.029 |
| C18.1n9 | DER2 | 3017~2823/1805~1734 | PLSR | Milk | 0.561 | 1.520 | 0.704 | 1.850 |
| C18.1n9 | SNV | 3017~2823/1805~1734 | PLSR | Milk | 0.561 | 1.526 | 0.647 | 1.694 |
| C18.1n9 | SG | 3017~2823/1805~1734 | PLSR | Milk | 0.541 | 1.490 | 0.736 | 1.960 |
| C18.1n9 | MSC | 3017~2823/1805~1734 | RFR | Milk | 0.485 | 1.391 | 0.501 | 1.396 |
| C18.1n9 | DER1 | 3017~2823/1805~1734 | RFR | Milk | 0.522 | 1.448 | 0.623 | 1.629 |
| C18.1n9 | DER2 | 3017~2823/1805~1734 | RFR | Milk | 0.538 | 1.474 | 0.668 | 1.711 |
| C18.1n9 | SNV | 3017~2823/1805~1734 | RFR | Milk | 0.473 | 1.376 | 0.528 | 1.443 |
| C18.1n9 | SG | 3017~2823/1805~1734 | RFR | Milk | 0.502 | 1.418 | 0.561 | 1.508 |
| C18.1n9 | MSC | 3017~2823/1805~1734 | RidgeR | Milk | 0.562 | 1.514 | 0.655 | 1.679 |
| C18.1n9 | DER1 | 3017~2823/1805~1734 | RidgeR | Milk | 0.589 | 1.562 | 0.759 | 1.926 |
| C18.1n9 | DER2 | 3017~2823/1805~1734 | RidgeR | Milk | 0.627 | 1.638 | 0.756 | 1.954 |
| C18.1n9 | SNV | 3017~2823/1805~1734 | RidgeR | Milk | 0.559 | 1.508 | 0.653 | 1.679 |
| C18.1n9 | SG | 3017~2823/1805~1734 | RidgeR | Milk | 0.550 | 1.494 | 0.701 | 1.789 |
| C18.1n9 | MSC | 4000~400 | RidgeR | Fat | 0.431 | 1.304 | 0.201 | 1.096 |
| C18.1n9 | DER1 | 4000~400 | RidgeR | Fat | 0.570 | 1.479 | 0.304 | 1.178 |
| C18.1n9 | DER2 | 4000~400 | RidgeR | Fat | 0.660 | 1.615 | 0.296 | 1.173 |
| C18.1n9 | SNV | 4000~400 | RidgeR | Fat | 0.443 | 1.320 | 0.208 | 1.098 |
| C18.1n9 | SG | 4000~400 | RidgeR | Fat | 0.409 | 1.285 | 0.197 | 1.088 |
| C18.1n9 | MSC | 4000~400 | RFR | Fat | 0.257 | 1.162 | 0.235 | 1.115 |
| C18.1n9 | DER1 | 4000~400 | RFR | Fat | 0.278 | 1.179 | 0.254 | 1.136 |
| C18.1n9 | DER2 | 4000~400 | RFR | Fat | 0.291 | 1.189 | 0.271 | 1.161 |
| C18.1n9 | SNV | 4000~400 | RFR | Fat | 0.247 | 1.154 | 0.178 | 1.054 |
| C18.1n9 | SG | 4000~400 | RFR | Fat | 0.238 | 1.148 | 0.191 | 1.088 |
| C18.1n9 | MSC | 4000~400 | PLSR | Fat | 0.299 | 1.206 | 0.008 | 1.010 |
| C18.1n9 | DER1 | 4000~400 | PLSR | Fat | 0.311 | 1.216 | 0.172 | 1.106 |
| C18.1n9 | DER2 | 4000~400 | PLSR | Fat | 0.291 | 1.196 | 0.145 | 1.088 |
| C18.1n9 | SNV | 4000~400 | PLSR | Fat | 0.288 | 1.195 | 0.037 | 1.026 |
| C18.1n9 | SG | 4000~400 | PLSR | Fat | 0.290 | 1.195 | 0.048 | 1.031 |
| C18.1n9 | MSC | 4000~400 | LassoR | Fat | 0.841 | 2.455 | 0.177 | 0.941 |
| C18.1n9 | DER1 | 4000~400 | LassoR | Fat | 0.970 | 5.368 | 0.217 | 0.871 |
| C18.1n9 | DER2 | 4000~400 | LassoR | Fat | 0.995 | 12.134 | 0.177 | 0.872 |
| C18.1n9 | SNV | 4000~400 | LassoR | Fat | 0.840 | 2.444 | 0.171 | 0.936 |
| C18.1n9 | SG | 4000~400 | LassoR | Fat | 0.786 | 2.128 | 0.197 | 0.955 |
| C18.1n9 | MSC | 4000~400 | LassoR | Milk | 0.859 | 2.629 | 0.456 | 1.312 |
| C18.1n9 | DER1 | 4000~400 | LassoR | Milk | 0.973 | 5.818 | 0.438 | 1.267 |
| C18.1n9 | DER2 | 4000~400 | LassoR | Milk | 0.994 | 11.576 | 0.497 | 1.367 |
| C18.1n9 | SNV | 4000~400 | LassoR | Milk | 0.863 | 2.659 | 0.461 | 1.318 |
| C18.1n9 | SG | 4000~400 | LassoR | Milk | 0.811 | 2.282 | 0.497 | 1.375 |
| C18.1n9 | MSC | 4000~400 | PLSR | Milk | 0.514 | 1.440 | 0.660 | 1.727 |
| C18.1n9 | DER1 | 4000~400 | PLSR | Milk | 0.526 | 1.484 | 0.543 | 1.488 |

| | | | | | | | | |
|---|---|---|---|---|---|---|---|---|
| C18.1n9 | DER2 | 4000~400 | PLSR | Milk | 0.510 | 1.474 | 0.610 | 1.612 |
| C18.1n9 | SNV | 4000~400 | PLSR | Milk | 0.515 | 1.441 | 0.660 | 1.725 |
| C18.1n9 | SG | 4000~400 | PLSR | Milk | 0.502 | 1.423 | 0.632 | 1.659 |
| C18.1n9 | MSC | 4000~400 | RFR | Milk | 0.497 | 1.411 | 0.564 | 1.523 |
| C18.1n9 | DER1 | 4000~400 | RFR | Milk | 0.523 | 1.450 | 0.644 | 1.674 |
| C18.1n9 | DER2 | 4000~400 | RFR | Milk | 0.534 | 1.464 | 0.663 | 1.691 |
| C18.1n9 | SNV | 4000~400 | RFR | Milk | 0.486 | 1.396 | 0.577 | 1.546 |
| C18.1n9 | SG | 4000~400 | RFR | Milk | 0.500 | 1.416 | 0.639 | 1.662 |
| C18.1n9 | MSC | 4000~400 | RidgeR | Milk | 0.594 | 1.568 | 0.651 | 1.678 |
| C18.1n9 | DER1 | 4000~400 | RidgeR | Milk | 0.667 | 1.721 | 0.706 | 1.805 |
| C18.1n9 | DER2 | 4000~400 | RidgeR | Milk | 0.728 | 1.855 | 0.699 | 1.754 |
| C18.1n9 | SNV | 4000~400 | RidgeR | Milk | 0.596 | 1.570 | 0.656 | 1.691 |
| C18.1n9 | SG | 4000~400 | RidgeR | Milk | 0.576 | 1.536 | 0.687 | 1.754 |
| C18.2n6 | MSC | 3017~2823/1805~1734 | LassoR | Fat | 0.175 | 1.103 | 0.137 | 1.070 |
| C18.2n6 | DER1 | 3017~2823/1805~1734 | LassoR | Fat | 0.239 | 1.146 | 0.145 | 1.067 |
| C18.2n6 | DER2 | 3017~2823/1805~1734 | LassoR | Fat | 0.248 | 1.152 | 0.074 | 1.017 |
| C18.2n6 | SNV | 3017~2823/1805~1734 | LassoR | Fat | 0.189 | 1.111 | 0.138 | 1.072 |
| C18.2n6 | SG | 3017~2823/1805~1734 | LassoR | Fat | 0.116 | 1.066 | 0.115 | 1.061 |
| C18.2n6 | MSC | 3017~2823/1805~1734 | RidgeR | Fat | 0.141 | 1.081 | 0.087 | 1.040 |
| C18.2n6 | DER1 | 3017~2823/1805~1734 | RidgeR | Fat | 0.172 | 1.099 | 0.129 | 1.068 |
| C18.2n6 | DER2 | 3017~2823/1805~1734 | RidgeR | Fat | 0.199 | 1.115 | 0.082 | 1.034 |
| C18.2n6 | SNV | 3017~2823/1805~1734 | RidgeR | Fat | 0.137 | 1.078 | 0.063 | 1.026 |
| C18.2n6 | SG | 3017~2823/1805~1734 | RidgeR | Fat | 0.103 | 1.058 | 0.122 | 1.064 |
| C18.2n6 | MSC | 3017~2823/1805~1734 | RFR | Fat | 0.080 | 1.028 | 0.071 | 1.005 |
| C18.2n6 | DER1 | 3017~2823/1805~1734 | RFR | Fat | 0.091 | 1.037 | 0.020 | 0.976 |
| C18.2n6 | DER2 | 3017~2823/1805~1734 | RFR | Fat | 0.065 | 1.024 | 0.056 | 1.013 |
| C18.2n6 | SNV | 3017~2823/1805~1734 | RFR | Fat | 0.063 | 1.009 | 0.062 | 0.996 |
| C18.2n6 | SG | 3017~2823/1805~1734 | RFR | Fat | 0.032 | 0.971 | 0.098 | 1.043 |
| C18.2n6 | MSC | 3017~2823/1805~1734 | PLSR | Fat | 0.092 | 1.054 | 0.103 | 1.063 |
| C18.2n6 | DER1 | 3017~2823/1805~1734 | PLSR | Fat | 0.068 | 1.038 | 0.062 | 1.039 |
| C18.2n6 | DER2 | 3017~2823/1805~1734 | PLSR | Fat | 0.059 | 1.039 | -0.025 | 0.994 |
| C18.2n6 | SNV | 3017~2823/1805~1734 | PLSR | Fat | 0.085 | 1.050 | 0.113 | 1.068 |
| C18.2n6 | SG | 3017~2823/1805~1734 | PLSR | Fat | 0.072 | 1.041 | 0.116 | 1.070 |
| C18.2n6 | MSC | 3017~2823/1805~1734 | LassoR | Milk | 0.622 | 1.629 | 0.627 | 1.611 |
| C18.2n6 | DER1 | 3017~2823/1805~1734 | LassoR | Milk | 0.626 | 1.638 | 0.608 | 1.563 |
| C18.2n6 | DER2 | 3017~2823/1805~1734 | LassoR | Milk | 0.654 | 1.702 | 0.603 | 1.547 |
| C18.2n6 | SNV | 3017~2823/1805~1734 | LassoR | Milk | 0.614 | 1.614 | 0.625 | 1.610 |
| C18.2n6 | SG | 3017~2823/1805~1734 | LassoR | Milk | 0.603 | 1.590 | 0.604 | 1.560 |
| C18.2n6 | MSC | 3017~2823/1805~1734 | PLSR | Milk | 0.600 | 1.586 | 0.615 | 1.622 |
| C18.2n6 | DER1 | 3017~2823/1805~1734 | PLSR | Milk | 0.570 | 1.538 | 0.506 | 1.432 |
| C18.2n6 | DER2 | 3017~2823/1805~1734 | PLSR | Milk | 0.524 | 1.471 | 0.547 | 1.496 |
| C18.2n6 | SNV | 3017~2823/1805~1734 | PLSR | Milk | 0.597 | 1.583 | 0.608 | 1.607 |
| C18.2n6 | SG | 3017~2823/1805~1734 | PLSR | Milk | 0.582 | 1.568 | 0.536 | 1.477 |
| C18.2n6 | MSC | 3017~2823/1805~1734 | RFR | Milk | 0.566 | 1.512 | 0.609 | 1.555 |
| C18.2n6 | DER1 | 3017~2823/1805~1734 | RFR | Milk | 0.541 | 1.471 | 0.620 | 1.607 |
| C18.2n6 | DER2 | 3017~2823/1805~1734 | RFR | Milk | 0.557 | 1.503 | 0.613 | 1.568 |
| C18.2n6 | SNV | 3017~2823/1805~1734 | RFR | Milk | 0.573 | 1.524 | 0.607 | 1.565 |
| C18.2n6 | SG | 3017~2823/1805~1734 | RFR | Milk | 0.573 | 1.529 | 0.551 | 1.447 |
| C18.2n6 | MSC | 3017~2823/1805~1734 | RidgeR | Milk | 0.607 | 1.599 | 0.619 | 1.601 |
| C18.2n6 | DER1 | 3017~2823/1805~1734 | RidgeR | Milk | 0.596 | 1.575 | 0.616 | 1.588 |

| | | | | | | | | |
|---|---|---|---|---|---|---|---|---|
| C18.2n6 | DER2 | 3017~2823/1805~1734 | RidgeR | Milk | 0.596 | 1.575 | 0.619 | 1.594 |
| C18.2n6 | SNV | 3017~2823/1805~1734 | RidgeR | Milk | 0.594 | 1.573 | 0.608 | 1.581 |
| C18.2n6 | SG | 3017~2823/1805~1734 | RidgeR | Milk | 0.574 | 1.534 | 0.605 | 1.568 |
| C18.2n6 | MSC | 4000~400 | RidgeR | Fat | 0.161 | 1.085 | 0.085 | 1.043 |
| C18.2n6 | DER1 | 4000~400 | RidgeR | Fat | 0.137 | 1.069 | 0.130 | 1.063 |
| C18.2n6 | DER2 | 4000~400 | RidgeR | Fat | 0.186 | 1.088 | 0.111 | 1.054 |
| C18.2n6 | SNV | 4000~400 | RidgeR | Fat | 0.156 | 1.082 | 0.083 | 1.042 |
| C18.2n6 | SG | 4000~400 | RidgeR | Fat | 0.130 | 1.069 | 0.148 | 1.075 |
| C18.2n6 | MSC | 4000~400 | RFR | Fat | 0.061 | 1.022 | 0.010 | 0.975 |
| C18.2n6 | DER1 | 4000~400 | RFR | Fat | 0.057 | 1.020 | 0.074 | 1.038 |
| C18.2n6 | DER2 | 4000~400 | RFR | Fat | 0.073 | 1.039 | 0.130 | 1.074 |
| C18.2n6 | SNV | 4000~400 | RFR | Fat | 0.068 | 1.029 | 0.055 | 1.011 |
| C18.2n6 | SG | 4000~400 | RFR | Fat | 0.077 | 1.029 | 0.055 | 1.016 |
| C18.2n6 | MSC | 4000~400 | PLSR | Fat | 0.073 | 1.041 | 0.019 | 1.016 |
| C18.2n6 | DER1 | 4000~400 | PLSR | Fat | 0.075 | 1.043 | 0.105 | 1.063 |
| C18.2n6 | DER2 | 4000~400 | PLSR | Fat | 0.078 | 1.044 | 0.104 | 1.063 |
| C18.2n6 | SNV | 4000~400 | PLSR | Fat | 0.074 | 1.042 | 0.020 | 1.016 |
| C18.2n6 | SG | 4000~400 | PLSR | Fat | 0.070 | 1.040 | 0.134 | 1.081 |
| C18.2n6 | MSC | 4000~400 | LassoR | Fat | 0.650 | 1.613 | 0.027 | 0.885 |
| C18.2n6 | DER1 | 4000~400 | LassoR | Fat | 0.895 | 2.618 | 0.078 | 0.920 |
| C18.2n6 | DER2 | 4000~400 | LassoR | Fat | 0.983 | 5.845 | 0.028 | 0.831 |
| C18.2n6 | SNV | 4000~400 | LassoR | Fat | 0.654 | 1.624 | 0.033 | 0.884 |
| C18.2n6 | SG | 4000~400 | LassoR | Fat | 0.520 | 1.409 | 0.032 | 0.900 |
| C18.2n6 | MSC | 4000~400 | LassoR | Milk | 0.857 | 2.624 | 0.427 | 1.198 |
| C18.2n6 | DER1 | 4000~400 | LassoR | Milk | 0.977 | 6.266 | 0.361 | 1.022 |
| C18.2n6 | DER2 | 4000~400 | LassoR | Milk | 0.997 | 16.062 | 0.446 | 1.148 |
| C18.2n6 | SNV | 4000~400 | LassoR | Milk | 0.863 | 2.675 | 0.424 | 1.191 |
| C18.2n6 | SG | 4000~400 | LassoR | Milk | 0.821 | 2.354 | 0.418 | 1.207 |
| C18.2n6 | MSC | 4000~400 | PLSR | Milk | 0.596 | 1.578 | 0.594 | 1.579 |
| C18.2n6 | DER1 | 4000~400 | PLSR | Milk | 0.554 | 1.503 | 0.565 | 1.526 |
| C18.2n6 | DER2 | 4000~400 | PLSR | Milk | 0.528 | 1.464 | 0.527 | 1.464 |
| C18.2n6 | SNV | 4000~400 | PLSR | Milk | 0.596 | 1.578 | 0.593 | 1.578 |
| C18.2n6 | SG | 4000~400 | PLSR | Milk | 0.572 | 1.534 | 0.571 | 1.536 |
| C18.2n6 | MSC | 4000~400 | RFR | Milk | 0.586 | 1.557 | 0.630 | 1.612 |
| C18.2n6 | DER1 | 4000~400 | RFR | Milk | 0.577 | 1.539 | 0.584 | 1.525 |
| C18.2n6 | DER2 | 4000~400 | RFR | Milk | 0.570 | 1.527 | 0.605 | 1.557 |
| C18.2n6 | SNV | 4000~400 | RFR | Milk | 0.566 | 1.521 | 0.597 | 1.548 |
| C18.2n6 | SG | 4000~400 | RFR | Milk | 0.592 | 1.568 | 0.621 | 1.600 |
| C18.2n6 | MSC | 4000~400 | RidgeR | Milk | 0.643 | 1.670 | 0.606 | 1.566 |
| C18.2n6 | DER1 | 4000~400 | RidgeR | Milk | 0.648 | 1.680 | 0.607 | 1.570 |
| C18.2n6 | DER2 | 4000~400 | RidgeR | Milk | 0.722 | 1.849 | 0.615 | 1.581 |
| C18.2n6 | SNV | 4000~400 | RidgeR | Milk | 0.643 | 1.671 | 0.610 | 1.574 |
| C18.2n6 | SG | 4000~400 | RidgeR | Milk | 0.631 | 1.646 | 0.603 | 1.561 |
| C18.3n3 | MSC | 3017~2823/1805~1734 | LassoR | Fat | 0.230 | 1.142 | 0.251 | 1.138 |
| C18.3n3 | DER1 | 3017~2823/1805~1734 | LassoR | Fat | 0.273 | 1.174 | 0.246 | 1.140 |
| C18.3n3 | DER2 | 3017~2823/1805~1734 | LassoR | Fat | 0.264 | 1.168 | 0.222 | 1.125 |
| C18.3n3 | SNV | 3017~2823/1805~1734 | LassoR | Fat | 0.230 | 1.142 | 0.245 | 1.134 |
| C18.3n3 | SG | 3017~2823/1805~1734 | LassoR | Fat | 0.239 | 1.149 | 0.224 | 1.124 |
| C18.3n3 | MSC | 3017~2823/1805~1734 | RidgeR | Fat | 0.209 | 1.126 | 0.203 | 1.101 |
| C18.3n3 | DER1 | 3017~2823/1805~1734 | RidgeR | Fat | 0.233 | 1.143 | 0.196 | 1.100 |

| | | | | | | | |
|---|---|---|---|---|---|---|---|
| C18.3n3 DER2 | 3017~2823/1805~1734 | RidgeR | Fat | 0.228 | 1.138 | 0.188 | 1.093 |
| C18.3n3 SNV | 3017~2823/1805~1734 | RidgeR | Fat | 0.209 | 1.126 | 0.200 | 1.099 |
| C18.3n3 SG | 3017~2823/1805~1734 | RidgeR | Fat | 0.231 | 1.141 | 0.190 | 1.096 |
| C18.3n3 MSC | 3017~2823/1805~1734 | RFR | Fat | 0.205 | 1.111 | 0.228 | 1.101 |
| C18.3n3 DER1 | 3017~2823/1805~1734 | RFR | Fat | 0.172 | 1.089 | 0.267 | 1.130 |
| C18.3n3 DER2 | 3017~2823/1805~1734 | RFR | Fat | 0.176 | 1.097 | 0.234 | 1.111 |
| C18.3n3 SNV | 3017~2823/1805~1734 | RFR | Fat | 0.203 | 1.108 | 0.170 | 1.060 |
| C18.3n3 SG | 3017~2823/1805~1734 | RFR | Fat | 0.178 | 1.089 | 0.227 | 1.107 |
| C18.3n3 MSC | 3017~2823/1805~1734 | PLSR | Fat | 0.198 | 1.119 | 0.165 | 1.101 |
| C18.3n3 DER1 | 3017~2823/1805~1734 | PLSR | Fat | 0.216 | 1.132 | 0.145 | 1.088 |
| C18.3n3 DER2 | 3017~2823/1805~1734 | PLSR | Fat | 0.214 | 1.130 | 0.147 | 1.089 |
| C18.3n3 SNV | 3017~2823/1805~1734 | PLSR | Fat | 0.167 | 1.102 | 0.214 | 1.135 |
| C18.3n3 SG | 3017~2823/1805~1734 | PLSR | Fat | 0.215 | 1.131 | 0.146 | 1.089 |
| C18.3n3 MSC | 3017~2823/1805~1734 | LassoR | Milk | 0.634 | 1.656 | 0.637 | 1.669 |
| C18.3n3 DER1 | 3017~2823/1805~1734 | LassoR | Milk | 0.701 | 1.830 | 0.605 | 1.586 |
| C18.3n3 DER2 | 3017~2823/1805~1734 | LassoR | Milk | 0.679 | 1.768 | 0.574 | 1.514 |
| C18.3n3 SNV | 3017~2823/1805~1734 | LassoR | Milk | 0.637 | 1.663 | 0.641 | 1.679 |
| C18.3n3 SG | 3017~2823/1805~1734 | LassoR | Milk | 0.616 | 1.618 | 0.574 | 1.532 |
| C18.3n3 MSC | 3017~2823/1805~1734 | PLSR | Milk | 0.615 | 1.618 | 0.633 | 1.660 |
| C18.3n3 DER1 | 3017~2823/1805~1734 | PLSR | Milk | 0.597 | 1.582 | 0.544 | 1.490 |
| C18.3n3 DER2 | 3017~2823/1805~1734 | PLSR | Milk | 0.601 | 1.589 | 0.525 | 1.460 |
| C18.3n3 SNV | 3017~2823/1805~1734 | PLSR | Milk | 0.608 | 1.604 | 0.639 | 1.675 |
| C18.3n3 SG | 3017~2823/1805~1734 | PLSR | Milk | 0.590 | 1.569 | 0.545 | 1.492 |
| C18.3n3 MSC | 3017~2823/1805~1734 | RFR | Milk | 0.583 | 1.546 | 0.649 | 1.697 |
| C18.3n3 DER1 | 3017~2823/1805~1734 | RFR | Milk | 0.596 | 1.571 | 0.672 | 1.732 |
| C18.3n3 DER2 | 3017~2823/1805~1734 | RFR | Milk | 0.590 | 1.563 | 0.654 | 1.702 |
| C18.3n3 SNV | 3017~2823/1805~1734 | RFR | Milk | 0.596 | 1.574 | 0.649 | 1.693 |
| C18.3n3 SG | 3017~2823/1805~1734 | RFR | Milk | 0.569 | 1.521 | 0.647 | 1.689 |
| C18.3n3 MSC | 3017~2823/1805~1734 | RidgeR | Milk | 0.628 | 1.642 | 0.638 | 1.669 |
| C18.3n3 DER1 | 3017~2823/1805~1734 | RidgeR | Milk | 0.629 | 1.645 | 0.581 | 1.540 |
| C18.3n3 DER2 | 3017~2823/1805~1734 | RidgeR | Milk | 0.638 | 1.665 | 0.573 | 1.527 |
| C18.3n3 SNV | 3017~2823/1805~1734 | RidgeR | Milk | 0.621 | 1.626 | 0.637 | 1.666 |
| C18.3n3 SG | 3017~2823/1805~1734 | RidgeR | Milk | 0.603 | 1.589 | 0.576 | 1.541 |
| C18.3n3 MSC | 4000~400 | RidgeR | Fat | 0.239 | 1.145 | 0.210 | 1.110 |
| C18.3n3 DER1 | 4000~400 | RidgeR | Fat | 0.269 | 1.164 | 0.205 | 1.107 |
| C18.3n3 DER2 | 4000~400 | RidgeR | Fat | 0.333 | 1.206 | 0.214 | 1.109 |
| C18.3n3 SNV | 4000~400 | RidgeR | Fat | 0.240 | 1.145 | 0.208 | 1.109 |
| C18.3n3 SG | 4000~400 | RidgeR | Fat | 0.241 | 1.145 | 0.205 | 1.107 |
| C18.3n3 MSC | 4000~400 | RFR | Fat | 0.228 | 1.140 | 0.205 | 1.098 |
| C18.3n3 DER1 | 4000~400 | RFR | Fat | 0.200 | 1.115 | 0.237 | 1.124 |
| C18.3n3 DER2 | 4000~400 | RFR | Fat | 0.179 | 1.103 | 0.250 | 1.135 |
| C18.3n3 SNV | 4000~400 | RFR | Fat | 0.228 | 1.139 | 0.201 | 1.109 |
| C18.3n3 SG | 4000~400 | RFR | Fat | 0.184 | 1.103 | 0.246 | 1.134 |
| C18.3n3 MSC | 4000~400 | PLSR | Fat | 0.184 | 1.110 | 0.164 | 1.101 |
| C18.3n3 DER1 | 4000~400 | PLSR | Fat | 0.222 | 1.136 | 0.178 | 1.110 |
| C18.3n3 DER2 | 4000~400 | PLSR | Fat | 0.211 | 1.129 | 0.164 | 1.101 |
| C18.3n3 SNV | 4000~400 | PLSR | Fat | 0.182 | 1.108 | 0.164 | 1.101 |
| C18.3n3 SG | 4000~400 | PLSR | Fat | 0.213 | 1.130 | 0.183 | 1.113 |
| C18.3n3 MSC | 4000~400 | LassoR | Fat | 0.712 | 1.807 | 0.060 | 0.927 |
| C18.3n3 DER1 | 4000~400 | LassoR | Fat | 0.894 | 2.826 | 0.100 | 0.917 |

| | | | | | | | |
|---|---|---|---|---|---|---|---|
| C18.3n3 DER2 | 4000~400 | LassoR | Fat | 0.977 | 5.618 | 0.221 | 1.035 |
| C18.3n3 SNV | 4000~400 | LassoR | Fat | 0.715 | 1.815 | 0.065 | 0.934 |
| C18.3n3 SG | 4000~400 | LassoR | Fat | 0.601 | 1.557 | 0.091 | 0.968 |
| C18.3n3 MSC | 4000~400 | LassoR | Milk | 0.865 | 2.699 | 0.444 | 1.308 |
| C18.3n3 DER1 | 4000~400 | LassoR | Milk | 0.966 | 5.188 | 0.451 | 1.288 |
| C18.3n3 DER2 | 4000~400 | LassoR | Milk | 0.996 | 15.536 | 0.436 | 1.274 |
| C18.3n3 SNV | 4000~400 | LassoR | Milk | 0.868 | 2.731 | 0.450 | 1.311 |
| C18.3n3 SG | 4000~400 | LassoR | Milk | 0.809 | 2.277 | 0.495 | 1.392 |
| C18.3n3 MSC | 4000~400 | PLSR | Milk | 0.611 | 1.609 | 0.625 | 1.643 |
| C18.3n3 DER1 | 4000~400 | PLSR | Milk | 0.601 | 1.589 | 0.568 | 1.531 |
| C18.3n3 DER2 | 4000~400 | PLSR | Milk | 0.582 | 1.559 | 0.557 | 1.511 |
| C18.3n3 SNV | 4000~400 | PLSR | Milk | 0.615 | 1.616 | 0.635 | 1.665 |
| C18.3n3 SG | 4000~400 | PLSR | Milk | 0.594 | 1.575 | 0.583 | 1.559 |
| C18.3n3 MSC | 4000~400 | RFR | Milk | 0.586 | 1.558 | 0.689 | 1.782 |
| C18.3n3 DER1 | 4000~400 | RFR | Milk | 0.613 | 1.610 | 0.661 | 1.721 |
| C18.3n3 DER2 | 4000~400 | RFR | Milk | 0.594 | 1.573 | 0.668 | 1.731 |
| C18.3n3 SNV | 4000~400 | RFR | Milk | 0.604 | 1.591 | 0.695 | 1.809 |
| C18.3n3 SG | 4000~400 | RFR | Milk | 0.603 | 1.591 | 0.698 | 1.824 |
| C18.3n3 MSC | 4000~400 | RidgeR | Milk | 0.652 | 1.691 | 0.621 | 1.626 |
| C18.3n3 DER1 | 4000~400 | RidgeR | Milk | 0.671 | 1.736 | 0.596 | 1.581 |
| C18.3n3 DER2 | 4000~400 | RidgeR | Milk | 0.746 | 1.944 | 0.593 | 1.575 |
| C18.3n3 SNV | 4000~400 | RidgeR | Milk | 0.661 | 1.713 | 0.638 | 1.659 |
| C18.3n3 SG | 4000~400 | RidgeR | Milk | 0.645 | 1.677 | 0.601 | 1.593 |
| C18.3n6 MSC | 3017~2823/1805~1734 | LassoR | Fat | 0.801 | 2.248 | 0.663 | 1.714 |
| C18.3n6 DER1 | 3017~2823/1805~1734 | LassoR | Fat | 0.830 | 2.428 | 0.620 | 1.579 |
| C18.3n6 DER2 | 3017~2823/1805~1734 | LassoR | Fat | 0.831 | 2.438 | 0.634 | 1.614 |
| C18.3n6 SNV | 3017~2823/1805~1734 | LassoR | Fat | 0.850 | 2.590 | 0.713 | 1.834 |
| C18.3n6 SG | 3017~2823/1805~1734 | LassoR | Fat | 0.728 | 1.923 | 0.605 | 1.593 |
| C18.3n6 MSC | 3017~2823/1805~1734 | RidgeR | Fat | 0.794 | 2.205 | 0.661 | 1.717 |
| C18.3n6 DER1 | 3017~2823/1805~1734 | RidgeR | Fat | 0.724 | 1.906 | 0.594 | 1.571 |
| C18.3n6 DER2 | 3017~2823/1805~1734 | RidgeR | Fat | 0.751 | 2.009 | 0.605 | 1.585 |
| C18.3n6 SNV | 3017~2823/1805~1734 | RidgeR | Fat | 0.835 | 2.465 | 0.709 | 1.834 |
| C18.3n6 SG | 3017~2823/1805~1734 | RidgeR | Fat | 0.726 | 1.914 | 0.603 | 1.588 |
| C18.3n6 MSC | 3017~2823/1805~1734 | RFR | Fat | 0.818 | 2.350 | 0.733 | 1.905 |
| C18.3n6 DER1 | 3017~2823/1805~1734 | RFR | Fat | 0.832 | 2.439 | 0.737 | 1.893 |
| C18.3n6 DER2 | 3017~2823/1805~1734 | RFR | Fat | 0.836 | 2.468 | 0.764 | 1.996 |
| C18.3n6 SNV | 3017~2823/1805~1734 | RFR | Fat | 0.817 | 2.340 | 0.727 | 1.873 |
| C18.3n6 SG | 3017~2823/1805~1734 | RFR | Fat | 0.837 | 2.477 | 0.728 | 1.830 |
| C18.3n6 MSC | 3017~2823/1805~1734 | PLSR | Fat | 0.792 | 2.208 | 0.661 | 1.729 |
| C18.3n6 DER1 | 3017~2823/1805~1734 | PLSR | Fat | 0.759 | 2.059 | 0.590 | 1.571 |
| C18.3n6 DER2 | 3017~2823/1805~1734 | PLSR | Fat | 0.756 | 2.046 | 0.599 | 1.590 |
| C18.3n6 SNV | 3017~2823/1805~1734 | PLSR | Fat | 0.830 | 2.434 | 0.700 | 1.837 |
| C18.3n6 SG | 3017~2823/1805~1734 | PLSR | Fat | 0.759 | 2.053 | 0.578 | 1.550 |
| C18.3n6 MSC | 3017~2823/1805~1734 | LassoR | Milk | 0.247 | 1.152 | 0.094 | 1.033 |
| C18.3n6 DER1 | 3017~2823/1805~1734 | LassoR | Milk | 0.235 | 1.144 | 0.068 | 1.017 |
| C18.3n6 DER2 | 3017~2823/1805~1734 | LassoR | Milk | 0.231 | 1.141 | 0.057 | 1.010 |
| C18.3n6 SNV | 3017~2823/1805~1734 | LassoR | Milk | 0.224 | 1.138 | 0.092 | 1.027 |
| C18.3n6 SG | 3017~2823/1805~1734 | LassoR | Milk | 0.192 | 1.115 | 0.055 | 1.007 |
| C18.3n6 MSC | 3017~2823/1805~1734 | PLSR | Milk | 0.097 | 1.057 | 0.042 | 1.028 |
| C18.3n6 DER1 | 3017~2823/1805~1734 | PLSR | Milk | 0.134 | 1.077 | 0.056 | 1.036 |

| | | | | | | | |
|---|---|---|---|---|---|---|---|
| C18.3n6 DER2 | 3017~2823/1805~1734 | PLSR | Milk | 0.133 | 1.077 | 0.050 | 1.032 |
| C18.3n6 SNV | 3017~2823/1805~1734 | PLSR | Milk | 0.180 | 1.108 | 0.051 | 1.033 |
| C18.3n6 SG | 3017~2823/1805~1734 | PLSR | Milk | 0.126 | 1.073 | -0.033 | 0.990 |
| C18.3n6 MSC | 3017~2823/1805~1734 | RFR | Milk | 0.176 | 1.084 | 0.039 | 0.927 |
| C18.3n6 DER1 | 3017~2823/1805~1734 | RFR | Milk | 0.197 | 1.106 | 0.126 | 1.028 |
| C18.3n6 DER2 | 3017~2823/1805~1734 | RFR | Milk | 0.195 | 1.096 | 0.078 | 1.002 |
| C18.3n6 SNV | 3017~2823/1805~1734 | RFR | Milk | 0.176 | 1.080 | 0.053 | 0.950 |
| C18.3n6 SG | 3017~2823/1805~1734 | RFR | Milk | 0.177 | 1.083 | 0.141 | 1.040 |
| C18.3n6 MSC | 3017~2823/1805~1734 | RidgeR | Milk | 0.126 | 1.071 | 0.047 | 1.021 |
| C18.3n6 DER1 | 3017~2823/1805~1734 | RidgeR | Milk | 0.155 | 1.089 | 0.079 | 1.041 |
| C18.3n6 DER2 | 3017~2823/1805~1734 | RidgeR | Milk | 0.161 | 1.093 | 0.075 | 1.037 |
| C18.3n6 SNV | 3017~2823/1805~1734 | RidgeR | Milk | 0.215 | 1.130 | 0.091 | 1.032 |
| C18.3n6 SG | 3017~2823/1805~1734 | RidgeR | Milk | 0.179 | 1.106 | 0.057 | 1.013 |
| C18.3n6 MSC | 4000~400 | RidgeR | Fat | 0.761 | 2.038 | 0.627 | 1.646 |
| C18.3n6 DER1 | 4000~400 | RidgeR | Fat | 0.790 | 2.167 | 0.615 | 1.618 |
| C18.3n6 DER2 | 4000~400 | RidgeR | Fat | 0.822 | 2.333 | 0.605 | 1.594 |
| C18.3n6 SNV | 4000~400 | RidgeR | Fat | 0.789 | 2.159 | 0.647 | 1.693 |
| C18.3n6 SG | 4000~400 | RidgeR | Fat | 0.744 | 1.980 | 0.613 | 1.615 |
| C18.3n6 MSC | 4000~400 | RFR | Fat | 0.828 | 2.413 | 0.748 | 1.991 |
| C18.3n6 DER1 | 4000~400 | RFR | Fat | 0.841 | 2.509 | 0.745 | 1.921 |
| C18.3n6 DER2 | 4000~400 | RFR | Fat | 0.841 | 2.516 | 0.763 | 1.999 |
| C18.3n6 SNV | 4000~400 | RFR | Fat | 0.829 | 2.421 | 0.752 | 1.979 |
| C18.3n6 SG | 4000~400 | RFR | Fat | 0.837 | 2.481 | 0.760 | 2.007 |
| C18.3n6 MSC | 4000~400 | PLSR | Fat | 0.722 | 1.905 | 0.618 | 1.627 |
| C18.3n6 DER1 | 4000~400 | PLSR | Fat | 0.707 | 1.855 | 0.616 | 1.625 |
| C18.3n6 DER2 | 4000~400 | PLSR | Fat | 0.710 | 1.941 | 0.374 | 1.272 |
| C18.3n6 SNV | 4000~400 | PLSR | Fat | 0.771 | 2.103 | 0.650 | 1.701 |
| C18.3n6 SG | 4000~400 | PLSR | Fat | 0.702 | 1.840 | 0.613 | 1.618 |
| C18.3n6 MSC | 4000~400 | LassoR | Fat | 0.938 | 3.994 | 0.507 | 1.278 |
| C18.3n6 DER1 | 4000~400 | LassoR | Fat | 0.997 | 18.088 | 0.477 | 1.180 |
| C18.3n6 DER2 | 4000~400 | LassoR | Fat | 0.998 | 20.902 | 0.495 | 1.259 |
| C18.3n6 SNV | 4000~400 | LassoR | Fat | 0.957 | 4.783 | 0.573 | 1.368 |
| C18.3n6 SG | 4000~400 | LassoR | Fat | 0.890 | 3.011 | 0.534 | 1.371 |
| C18.3n6 MSC | 4000~400 | LassoR | Milk | 0.723 | 1.818 | 0.042 | 0.855 |
| C18.3n6 DER1 | 4000~400 | LassoR | Milk | 0.911 | 2.996 | 0.056 | 0.790 |
| C18.3n6 DER2 | 4000~400 | LassoR | Milk | 1.000 | 34.522 | 0.015 | 0.702 |
| C18.3n6 SNV | 4000~400 | LassoR | Milk | 0.719 | 1.819 | 0.050 | 0.867 |
| C18.3n6 SG | 4000~400 | LassoR | Milk | 0.611 | 1.571 | 0.052 | 0.904 |
| C18.3n6 MSC | 4000~400 | PLSR | Milk | 0.115 | 1.067 | -0.033 | 0.990 |
| C18.3n6 DER1 | 4000~400 | PLSR | Milk | 0.123 | 1.070 | 0.080 | 1.049 |
| C18.3n6 DER2 | 4000~400 | PLSR | Milk | 0.032 | 1.074 | -1.152 | 0.686 |
| C18.3n6 SNV | 4000~400 | PLSR | Milk | 0.124 | 1.072 | -0.045 | 0.984 |
| C18.3n6 SG | 4000~400 | PLSR | Milk | 0.138 | 1.082 | -0.099 | 0.960 |
| C18.3n6 MSC | 4000~400 | RFR | Milk | 0.233 | 1.141 | 0.068 | 0.978 |
| C18.3n6 DER1 | 4000~400 | RFR | Milk | 0.193 | 1.113 | 0.087 | 1.012 |
| C18.3n6 DER2 | 4000~400 | RFR | Milk | 0.191 | 1.113 | 0.085 | 1.021 |
| C18.3n6 SNV | 4000~400 | RFR | Milk | 0.216 | 1.127 | 0.077 | 0.983 |
| C18.3n6 SG | 4000~400 | RFR | Milk | 0.203 | 1.117 | 0.098 | 1.011 |
| C18.3n6 MSC | 4000~400 | RidgeR | Milk | 0.203 | 1.117 | 0.051 | 1.016 |
| C18.3n6 DER1 | 4000~400 | RidgeR | Milk | 0.307 | 1.182 | 0.061 | 1.021 |

| | | | | | | | | |
|---|---|---|---|---|---|---|---|---|
| C18.3n6 | DER2 | 4000~400 | RidgeR | Milk | 0.655 | 1.517 | 0.053 | 0.987 |
| C18.3n6 | SNV | 4000~400 | RidgeR | Milk | 0.305 | 1.186 | 0.062 | 1.010 |
| C18.3n6 | SG | 4000~400 | RidgeR | Milk | 0.235 | 1.141 | 0.044 | 1.003 |
| C20.0 | MSC | 3017~2823/1805~1734 | LassoR | Fat | 0.772 | 2.096 | 0.781 | 2.126 |
| C20.0 | DER1 | 3017~2823/1805~1734 | LassoR | Fat | 0.768 | 2.079 | 0.732 | 1.927 |
| C20.0 | DER2 | 3017~2823/1805~1734 | LassoR | Fat | 0.747 | 1.992 | 0.699 | 1.827 |
| C20.0 | SNV | 3017~2823/1805~1734 | LassoR | Fat | 0.790 | 2.184 | 0.801 | 2.240 |
| C20.0 | SG | 3017~2823/1805~1734 | LassoR | Fat | 0.728 | 1.920 | 0.704 | 1.840 |
| C20.0 | MSC | 3017~2823/1805~1734 | RidgeR | Fat | 0.736 | 1.951 | 0.750 | 1.985 |
| C20.0 | DER1 | 3017~2823/1805~1734 | RidgeR | Fat | 0.708 | 1.854 | 0.690 | 1.779 |
| C20.0 | DER2 | 3017~2823/1805~1734 | RidgeR | Fat | 0.726 | 1.912 | 0.685 | 1.773 |
| C20.0 | SNV | 3017~2823/1805~1734 | RidgeR | Fat | 0.746 | 1.987 | 0.757 | 2.019 |
| C20.0 | SG | 3017~2823/1805~1734 | RidgeR | Fat | 0.688 | 1.794 | 0.661 | 1.711 |
| C20.0 | MSC | 3017~2823/1805~1734 | RFR | Fat | 0.710 | 1.859 | 0.713 | 1.873 |
| C20.0 | DER1 | 3017~2823/1805~1734 | RFR | Fat | 0.690 | 1.799 | 0.683 | 1.768 |
| C20.0 | DER2 | 3017~2823/1805~1734 | RFR | Fat | 0.709 | 1.857 | 0.693 | 1.792 |
| C20.0 | SNV | 3017~2823/1805~1734 | RFR | Fat | 0.713 | 1.869 | 0.689 | 1.799 |
| C20.0 | SG | 3017~2823/1805~1734 | RFR | Fat | 0.668 | 1.734 | 0.683 | 1.752 |
| C20.0 | MSC | 3017~2823/1805~1734 | PLSR | Fat | 0.740 | 1.978 | 0.806 | 2.283 |
| C20.0 | DER1 | 3017~2823/1805~1734 | PLSR | Fat | 0.690 | 1.808 | 0.697 | 1.827 |
| C20.0 | DER2 | 3017~2823/1805~1734 | PLSR | Fat | 0.679 | 1.778 | 0.674 | 1.762 |
| C20.0 | SNV | 3017~2823/1805~1734 | PLSR | Fat | 0.756 | 2.037 | 0.817 | 2.352 |
| C20.0 | SG | 3017~2823/1805~1734 | PLSR | Fat | 0.703 | 1.856 | 0.744 | 1.990 |
| C20.0 | MSC | 3017~2823/1805~1734 | LassoR | Milk | 0.596 | 1.576 | 0.654 | 1.702 |
| C20.0 | DER1 | 3017~2823/1805~1734 | LassoR | Milk | 0.650 | 1.693 | 0.676 | 1.748 |
| C20.0 | DER2 | 3017~2823/1805~1734 | LassoR | Milk | 0.667 | 1.735 | 0.675 | 1.744 |
| C20.0 | SNV | 3017~2823/1805~1734 | LassoR | Milk | 0.606 | 1.596 | 0.657 | 1.710 |
| C20.0 | SG | 3017~2823/1805~1734 | LassoR | Milk | 0.599 | 1.583 | 0.669 | 1.739 |
| C20.0 | MSC | 3017~2823/1805~1734 | PLSR | Milk | 0.559 | 1.512 | 0.646 | 1.692 |
| C20.0 | DER1 | 3017~2823/1805~1734 | PLSR | Milk | 0.558 | 1.513 | 0.669 | 1.748 |
| C20.0 | DER2 | 3017~2823/1805~1734 | PLSR | Milk | 0.548 | 1.501 | 0.637 | 1.670 |
| C20.0 | SNV | 3017~2823/1805~1734 | PLSR | Milk | 0.561 | 1.517 | 0.658 | 1.720 |
| C20.0 | SG | 3017~2823/1805~1734 | PLSR | Milk | 0.519 | 1.456 | 0.712 | 1.876 |
| C20.0 | MSC | 3017~2823/1805~1734 | RFR | Milk | 0.525 | 1.450 | 0.520 | 1.423 |
| C20.0 | DER1 | 3017~2823/1805~1734 | RFR | Milk | 0.535 | 1.467 | 0.563 | 1.492 |
| C20.0 | DER2 | 3017~2823/1805~1734 | RFR | Milk | 0.555 | 1.499 | 0.562 | 1.510 |
| C20.0 | SNV | 3017~2823/1805~1734 | RFR | Milk | 0.508 | 1.421 | 0.544 | 1.465 |
| C20.0 | SG | 3017~2823/1805~1734 | RFR | Milk | 0.570 | 1.528 | 0.542 | 1.469 |
| C20.0 | MSC | 3017~2823/1805~1734 | RidgeR | Milk | 0.565 | 1.520 | 0.618 | 1.617 |
| C20.0 | DER1 | 3017~2823/1805~1734 | RidgeR | Milk | 0.579 | 1.542 | 0.659 | 1.696 |
| C20.0 | DER2 | 3017~2823/1805~1734 | RidgeR | Milk | 0.602 | 1.586 | 0.667 | 1.715 |
| C20.0 | SNV | 3017~2823/1805~1734 | RidgeR | Milk | 0.555 | 1.501 | 0.606 | 1.593 |
| C20.0 | SG | 3017~2823/1805~1734 | RidgeR | Milk | 0.551 | 1.495 | 0.610 | 1.605 |
| C20.0 | MSC | 4000~400 | RidgeR | Fat | 0.720 | 1.873 | 0.704 | 1.758 |
| C20.0 | DER1 | 4000~400 | RidgeR | Fat | 0.754 | 2.002 | 0.682 | 1.742 |
| C20.0 | DER2 | 4000~400 | RidgeR | Fat | 0.806 | 2.228 | 0.680 | 1.735 |
| C20.0 | SNV | 4000~400 | RidgeR | Fat | 0.733 | 1.918 | 0.708 | 1.776 |
| C20.0 | SG | 4000~400 | RidgeR | Fat | 0.707 | 1.847 | 0.652 | 1.676 |
| C20.0 | MSC | 4000~400 | RFR | Fat | 0.704 | 1.839 | 0.707 | 1.808 |
| C20.0 | DER1 | 4000~400 | RFR | Fat | 0.698 | 1.822 | 0.694 | 1.769 |

| | | | | | | | | |
|---|---|---|---|---|---|---|---|---|
| C20.0 | DER2 | 4000~400 | RFR | Fat | 0.707 | 1.852 | 0.684 | 1.745 |
| C20.0 | SNV | 4000~400 | RFR | Fat | 0.701 | 1.832 | 0.696 | 1.779 |
| C20.0 | SG | 4000~400 | RFR | Fat | 0.695 | 1.813 | 0.732 | 1.865 |
| C20.0 | MSC | 4000~400 | PLSR | Fat | 0.677 | 1.767 | 0.672 | 1.758 |
| C20.0 | DER1 | 4000~400 | PLSR | Fat | 0.667 | 1.740 | 0.687 | 1.799 |
| C20.0 | DER2 | 4000~400 | PLSR | Fat | 0.660 | 1.765 | 0.683 | 1.789 |
| C20.0 | SNV | 4000~400 | PLSR | Fat | 0.691 | 1.807 | 0.685 | 1.792 |
| C20.0 | SG | 4000~400 | PLSR | Fat | 0.664 | 1.733 | 0.654 | 1.711 |
| C20.0 | MSC | 4000~400 | LassoR | Fat | 0.898 | 3.113 | 0.643 | 1.683 |
| C20.0 | DER1 | 4000~400 | LassoR | Fat | 0.973 | 5.941 | 0.670 | 1.751 |
| C20.0 | DER2 | 4000~400 | LassoR | Fat | 0.999 | 28.972 | 0.648 | 1.691 |
| C20.0 | SNV | 4000~400 | LassoR | Fat | 0.909 | 3.299 | 0.670 | 1.746 |
| C20.0 | SG | 4000~400 | LassoR | Fat | 0.860 | 2.673 | 0.669 | 1.748 |
| C20.0 | MSC | 4000~400 | LassoR | Milk | 0.805 | 2.241 | 0.494 | 1.386 |
| C20.0 | DER1 | 4000~400 | LassoR | Milk | 0.969 | 5.412 | 0.355 | 1.094 |
| C20.0 | DER2 | 4000~400 | LassoR | Milk | 0.993 | 10.989 | 0.405 | 1.214 |
| C20.0 | SNV | 4000~400 | LassoR | Milk | 0.830 | 2.394 | 0.519 | 1.408 |
| C20.0 | SG | 4000~400 | LassoR | Milk | 0.768 | 2.062 | 0.590 | 1.561 |
| C20.0 | MSC | 4000~400 | PLSR | Milk | 0.519 | 1.446 | 0.574 | 1.542 |
| C20.0 | DER1 | 4000~400 | PLSR | Milk | 0.523 | 1.455 | 0.619 | 1.631 |
| C20.0 | DER2 | 4000~400 | PLSR | Milk | 0.524 | 1.459 | 0.639 | 1.675 |
| C20.0 | SNV | 4000~400 | PLSR | Milk | 0.522 | 1.450 | 0.574 | 1.542 |
| C20.0 | SG | 4000~400 | PLSR | Milk | 0.511 | 1.434 | 0.621 | 1.635 |
| C20.0 | MSC | 4000~400 | RFR | Milk | 0.496 | 1.409 | 0.594 | 1.580 |
| C20.0 | DER1 | 4000~400 | RFR | Milk | 0.562 | 1.515 | 0.645 | 1.673 |
| C20.0 | DER2 | 4000~400 | RFR | Milk | 0.557 | 1.506 | 0.619 | 1.597 |
| C20.0 | SNV | 4000~400 | RFR | Milk | 0.523 | 1.450 | 0.593 | 1.574 |
| C20.0 | SG | 4000~400 | RFR | Milk | 0.539 | 1.476 | 0.621 | 1.631 |
| C20.0 | MSC | 4000~400 | RidgeR | Milk | 0.554 | 1.492 | 0.574 | 1.533 |
| C20.0 | DER1 | 4000~400 | RidgeR | Milk | 0.653 | 1.688 | 0.646 | 1.679 |
| C20.0 | DER2 | 4000~400 | RidgeR | Milk | 0.718 | 1.832 | 0.663 | 1.703 |
| C20.0 | SNV | 4000~400 | RidgeR | Milk | 0.571 | 1.523 | 0.588 | 1.559 |
| C20.0 | SG | 4000~400 | RidgeR | Milk | 0.556 | 1.500 | 0.620 | 1.620 |
| C20.1 | MSC | 3017~2823/1805~1734 | LassoR | Fat | 0.802 | 2.250 | 0.718 | 1.860 |
| C20.1 | DER1 | 3017~2823/1805~1734 | LassoR | Fat | 0.809 | 2.293 | 0.667 | 1.676 |
| C20.1 | DER2 | 3017~2823/1805~1734 | LassoR | Fat | 0.804 | 2.264 | 0.666 | 1.693 |
| C20.1 | SNV | 3017~2823/1805~1734 | LassoR | Fat | 0.804 | 2.261 | 0.740 | 1.951 |
| C20.1 | SG | 3017~2823/1805~1734 | LassoR | Fat | 0.733 | 1.940 | 0.650 | 1.662 |
| C20.1 | MSC | 3017~2823/1805~1734 | RidgeR | Fat | 0.739 | 1.961 | 0.682 | 1.762 |
| C20.1 | DER1 | 3017~2823/1805~1734 | RidgeR | Fat | 0.725 | 1.912 | 0.644 | 1.648 |
| C20.1 | DER2 | 3017~2823/1805~1734 | RidgeR | Fat | 0.752 | 2.009 | 0.647 | 1.666 |
| C20.1 | SNV | 3017~2823/1805~1734 | RidgeR | Fat | 0.759 | 2.040 | 0.717 | 1.875 |
| C20.1 | SG | 3017~2823/1805~1734 | RidgeR | Fat | 0.701 | 1.831 | 0.634 | 1.623 |
| C20.1 | MSC | 3017~2823/1805~1734 | RFR | Fat | 0.724 | 1.907 | 0.677 | 1.733 |
| C20.1 | DER1 | 3017~2823/1805~1734 | RFR | Fat | 0.724 | 1.905 | 0.685 | 1.753 |
| C20.1 | DER2 | 3017~2823/1805~1734 | RFR | Fat | 0.724 | 1.906 | 0.722 | 1.881 |
| C20.1 | SNV | 3017~2823/1805~1734 | RFR | Fat | 0.721 | 1.894 | 0.690 | 1.780 |
| C20.1 | SG | 3017~2823/1805~1734 | RFR | Fat | 0.696 | 1.813 | 0.695 | 1.778 |
| C20.1 | MSC | 3017~2823/1805~1734 | PLSR | Fat | 0.754 | 2.025 | 0.689 | 1.806 |
| C20.1 | DER1 | 3017~2823/1805~1734 | PLSR | Fat | 0.700 | 1.848 | 0.619 | 1.630 |

| | | | | | | | | |
|---|---|---|---|---|---|---|---|---|
| C20.1 | DER2 | 3017~2823/1805~1734 | PLSR | Fat | 0.706 | 1.863 | 0.634 | 1.664 |
| C20.1 | SNV | 3017~2823/1805~1734 | PLSR | Fat | 0.773 | 2.111 | 0.729 | 1.934 |
| C20.1 | SG | 3017~2823/1805~1734 | PLSR | Fat | 0.715 | 1.889 | 0.639 | 1.675 |
| C20.1 | MSC | 3017~2823/1805~1734 | LassoR | Milk | 0.599 | 1.583 | 0.421 | 1.321 |
| C20.1 | DER1 | 3017~2823/1805~1734 | LassoR | Milk | 0.623 | 1.632 | 0.373 | 1.267 |
| C20.1 | DER2 | 3017~2823/1805~1734 | LassoR | Milk | 0.619 | 1.623 | 0.419 | 1.319 |
| C20.1 | SNV | 3017~2823/1805~1734 | LassoR | Milk | 0.602 | 1.589 | 0.429 | 1.330 |
| C20.1 | SG | 3017~2823/1805~1734 | LassoR | Milk | 0.589 | 1.562 | 0.447 | 1.351 |
| C20.1 | MSC | 3017~2823/1805~1734 | PLSR | Milk | 0.499 | 1.428 | 0.420 | 1.321 |
| C20.1 | DER1 | 3017~2823/1805~1734 | PLSR | Milk | 0.517 | 1.450 | 0.383 | 1.281 |
| C20.1 | DER2 | 3017~2823/1805~1734 | PLSR | Milk | 0.502 | 1.426 | 0.340 | 1.239 |
| C20.1 | SNV | 3017~2823/1805~1734 | PLSR | Milk | 0.499 | 1.427 | 0.405 | 1.304 |
| C20.1 | SG | 3017~2823/1805~1734 | PLSR | Milk | 0.539 | 1.482 | 0.487 | 1.405 |
| C20.1 | MSC | 3017~2823/1805~1734 | RFR | Milk | 0.441 | 1.339 | 0.212 | 1.124 |
| C20.1 | DER1 | 3017~2823/1805~1734 | RFR | Milk | 0.437 | 1.335 | 0.289 | 1.193 |
| C20.1 | DER2 | 3017~2823/1805~1734 | RFR | Milk | 0.445 | 1.340 | 0.300 | 1.199 |
| C20.1 | SNV | 3017~2823/1805~1734 | RFR | Milk | 0.445 | 1.345 | 0.212 | 1.123 |
| C20.1 | SG | 3017~2823/1805~1734 | RFR | Milk | 0.399 | 1.290 | 0.273 | 1.174 |
| C20.1 | MSC | 3017~2823/1805~1734 | RidgeR | Milk | 0.489 | 1.402 | 0.323 | 1.223 |
| C20.1 | DER1 | 3017~2823/1805~1734 | RidgeR | Milk | 0.566 | 1.518 | 0.337 | 1.235 |
| C20.1 | DER2 | 3017~2823/1805~1734 | RidgeR | Milk | 0.575 | 1.533 | 0.361 | 1.258 |
| C20.1 | SNV | 3017~2823/1805~1734 | RidgeR | Milk | 0.504 | 1.422 | 0.292 | 1.194 |
| C20.1 | SG | 3017~2823/1805~1734 | RidgeR | Milk | 0.510 | 1.431 | 0.307 | 1.209 |
| C20.1 | MSC | 4000~400 | RidgeR | Fat | 0.751 | 1.984 | 0.670 | 1.748 |
| C20.1 | DER1 | 4000~400 | RidgeR | Fat | 0.788 | 2.156 | 0.701 | 1.816 |
| C20.1 | DER2 | 4000~400 | RidgeR | Fat | 0.830 | 2.376 | 0.702 | 1.813 |
| C20.1 | SNV | 4000~400 | RidgeR | Fat | 0.779 | 2.105 | 0.693 | 1.811 |
| C20.1 | SG | 4000~400 | RidgeR | Fat | 0.738 | 1.947 | 0.663 | 1.712 |
| C20.1 | MSC | 4000~400 | RFR | Fat | 0.726 | 1.913 | 0.698 | 1.823 |
| C20.1 | DER1 | 4000~400 | RFR | Fat | 0.763 | 2.058 | 0.748 | 1.992 |
| C20.1 | DER2 | 4000~400 | RFR | Fat | 0.765 | 2.060 | 0.760 | 2.040 |
| C20.1 | SNV | 4000~400 | RFR | Fat | 0.738 | 1.959 | 0.703 | 1.839 |
| C20.1 | SG | 4000~400 | RFR | Fat | 0.735 | 1.946 | 0.732 | 1.933 |
| C20.1 | MSC | 4000~400 | PLSR | Fat | 0.694 | 1.836 | 0.634 | 1.664 |
| C20.1 | DER1 | 4000~400 | PLSR | Fat | 0.715 | 1.879 | 0.666 | 1.741 |
| C20.1 | DER2 | 4000~400 | PLSR | Fat | 0.717 | 1.926 | 0.656 | 1.716 |
| C20.1 | SNV | 4000~400 | PLSR | Fat | 0.737 | 1.967 | 0.658 | 1.722 |
| C20.1 | SG | 4000~400 | PLSR | Fat | 0.697 | 1.825 | 0.661 | 1.728 |
| C20.1 | MSC | 4000~400 | LassoR | Fat | 0.949 | 4.396 | 0.693 | 1.664 |
| C20.1 | DER1 | 4000~400 | LassoR | Fat | 0.985 | 8.035 | 0.649 | 1.549 |
| C20.1 | DER2 | 4000~400 | LassoR | Fat | 0.999 | 28.171 | 0.653 | 1.461 |
| C20.1 | SNV | 4000~400 | LassoR | Fat | 0.947 | 4.312 | 0.700 | 1.704 |
| C20.1 | SG | 4000~400 | LassoR | Fat | 0.918 | 3.487 | 0.694 | 1.669 |
| C20.1 | MSC | 4000~400 | LassoR | Milk | 0.876 | 2.804 | 0.298 | 1.163 |
| C20.1 | DER1 | 4000~400 | LassoR | Milk | 0.990 | 9.478 | 0.141 | 0.983 |
| C20.1 | DER2 | 4000~400 | LassoR | Milk | 0.996 | 15.290 | 0.262 | 1.117 |
| C20.1 | SNV | 4000~400 | LassoR | Milk | 0.897 | 3.075 | 0.265 | 1.121 |
| C20.1 | SG | 4000~400 | LassoR | Milk | 0.833 | 2.429 | 0.314 | 1.185 |
| C20.1 | MSC | 4000~400 | PLSR | Milk | 0.517 | 1.444 | 0.333 | 1.232 |
| C20.1 | DER1 | 4000~400 | PLSR | Milk | 0.549 | 1.495 | 0.346 | 1.244 |

| | | | | | | | | |
|---|---|---|---|---|---|---|---|---|
| C20.1 | DER2 | 4000~400 | PLSR | Milk | 0.518 | 1.508 | 0.160 | 1.098 |
| C20.1 | SNV | 4000~400 | PLSR | Milk | 0.520 | 1.448 | 0.325 | 1.225 |
| C20.1 | SG | 4000~400 | PLSR | Milk | 0.525 | 1.456 | 0.341 | 1.240 |
| C20.1 | MSC | 4000~400 | RFR | Milk | 0.487 | 1.396 | 0.339 | 1.236 |
| C20.1 | DER1 | 4000~400 | RFR | Milk | 0.546 | 1.484 | 0.332 | 1.226 |
| C20.1 | DER2 | 4000~400 | RFR | Milk | 0.523 | 1.434 | 0.306 | 1.204 |
| C20.1 | SNV | 4000~400 | RFR | Milk | 0.501 | 1.417 | 0.307 | 1.202 |
| C20.1 | SG | 4000~400 | RFR | Milk | 0.478 | 1.387 | 0.321 | 1.217 |
| C20.1 | MSC | 4000~400 | RidgeR | Milk | 0.597 | 1.567 | 0.361 | 1.256 |
| C20.1 | DER1 | 4000~400 | RidgeR | Milk | 0.684 | 1.754 | 0.356 | 1.251 |
| C20.1 | DER2 | 4000~400 | RidgeR | Milk | 0.746 | 1.907 | 0.344 | 1.238 |
| C20.1 | SNV | 4000~400 | RidgeR | Milk | 0.600 | 1.573 | 0.349 | 1.245 |
| C20.1 | SG | 4000~400 | RidgeR | Milk | 0.598 | 1.572 | 0.357 | 1.252 |
| C20.3n6 | MSC | 3017~2823/1805~1734 | LassoR | Fat | 0.362 | 1.253 | 0.380 | 1.268 |
| C20.3n6 | DER1 | 3017~2823/1805~1734 | LassoR | Fat | 0.468 | 1.371 | 0.368 | 1.255 |
| C20.3n6 | DER2 | 3017~2823/1805~1734 | LassoR | Fat | 0.443 | 1.340 | 0.337 | 1.221 |
| C20.3n6 | SNV | 3017~2823/1805~1734 | LassoR | Fat | 0.385 | 1.276 | 0.375 | 1.265 |
| C20.3n6 | SG | 3017~2823/1805~1734 | LassoR | Fat | 0.320 | 1.213 | 0.371 | 1.263 |
| C20.3n6 | MSC | 3017~2823/1805~1734 | RidgeR | Fat | 0.243 | 1.151 | 0.330 | 1.222 |
| C20.3n6 | DER1 | 3017~2823/1805~1734 | RidgeR | Fat | 0.289 | 1.187 | 0.363 | 1.254 |
| C20.3n6 | DER2 | 3017~2823/1805~1734 | RidgeR | Fat | 0.319 | 1.211 | 0.316 | 1.215 |
| C20.3n6 | SNV | 3017~2823/1805~1734 | RidgeR | Fat | 0.241 | 1.150 | 0.336 | 1.225 |
| C20.3n6 | SG | 3017~2823/1805~1734 | RidgeR | Fat | 0.216 | 1.131 | 0.313 | 1.203 |
| C20.3n6 | MSC | 3017~2823/1805~1734 | RFR | Fat | 0.141 | 1.067 | 0.259 | 1.161 |
| C20.3n6 | DER1 | 3017~2823/1805~1734 | RFR | Fat | 0.132 | 1.064 | 0.344 | 1.239 |
| C20.3n6 | DER2 | 3017~2823/1805~1734 | RFR | Fat | 0.134 | 1.070 | 0.266 | 1.168 |
| C20.3n6 | SNV | 3017~2823/1805~1734 | RFR | Fat | 0.147 | 1.071 | 0.284 | 1.184 |
| C20.3n6 | SG | 3017~2823/1805~1734 | RFR | Fat | 0.093 | 1.027 | 0.308 | 1.206 |
| C20.3n6 | MSC | 3017~2823/1805~1734 | PLSR | Fat | 0.233 | 1.154 | 0.389 | 1.287 |
| C20.3n6 | DER1 | 3017~2823/1805~1734 | PLSR | Fat | 0.301 | 1.208 | 0.341 | 1.240 |
| C20.3n6 | DER2 | 3017~2823/1805~1734 | PLSR | Fat | 0.239 | 1.153 | 0.275 | 1.182 |
| C20.3n6 | SNV | 3017~2823/1805~1734 | PLSR | Fat | 0.225 | 1.148 | 0.375 | 1.273 |
| C20.3n6 | SG | 3017~2823/1805~1734 | PLSR | Fat | 0.297 | 1.206 | 0.349 | 1.247 |
| C20.3n6 | MSC | 3017~2823/1805~1734 | LassoR | Milk | 0.568 | 1.524 | 0.564 | 1.514 |
| C20.3n6 | DER1 | 3017~2823/1805~1734 | LassoR | Milk | 0.637 | 1.661 | 0.500 | 1.383 |
| C20.3n6 | DER2 | 3017~2823/1805~1734 | LassoR | Milk | 0.651 | 1.695 | 0.517 | 1.423 |
| C20.3n6 | SNV | 3017~2823/1805~1734 | LassoR | Milk | 0.582 | 1.550 | 0.565 | 1.516 |
| C20.3n6 | SG | 3017~2823/1805~1734 | LassoR | Milk | 0.537 | 1.472 | 0.435 | 1.285 |
| C20.3n6 | MSC | 3017~2823/1805~1734 | PLSR | Milk | 0.510 | 1.439 | 0.482 | 1.399 |
| C20.3n6 | DER1 | 3017~2823/1805~1734 | PLSR | Milk | 0.511 | 1.437 | 0.363 | 1.261 |
| C20.3n6 | DER2 | 3017~2823/1805~1734 | PLSR | Milk | 0.490 | 1.412 | 0.421 | 1.323 |
| C20.3n6 | SNV | 3017~2823/1805~1734 | PLSR | Milk | 0.513 | 1.444 | 0.484 | 1.401 |
| C20.3n6 | SG | 3017~2823/1805~1734 | PLSR | Milk | 0.474 | 1.394 | 0.567 | 1.530 |
| C20.3n6 | MSC | 3017~2823/1805~1734 | RFR | Milk | 0.458 | 1.354 | 0.572 | 1.535 |
| C20.3n6 | DER1 | 3017~2823/1805~1734 | RFR | Milk | 0.442 | 1.334 | 0.563 | 1.520 |
| C20.3n6 | DER2 | 3017~2823/1805~1734 | RFR | Milk | 0.469 | 1.373 | 0.509 | 1.425 |
| C20.3n6 | SNV | 3017~2823/1805~1734 | RFR | Milk | 0.468 | 1.370 | 0.567 | 1.528 |
| C20.3n6 | SG | 3017~2823/1805~1734 | RFR | Milk | 0.439 | 1.332 | 0.492 | 1.395 |
| C20.3n6 | MSC | 3017~2823/1805~1734 | RidgeR | Milk | 0.496 | 1.411 | 0.564 | 1.522 |
| C20.3n6 | DER1 | 3017~2823/1805~1734 | RidgeR | Milk | 0.543 | 1.479 | 0.461 | 1.338 |

| | | | | | | | |
|---|---|---|---|---|---|---|---|
| C20.3n6 DER2 | 3017~2823/1805~1734 | RidgeR | Milk | 0.572 | 1.530 | 0.435 | 1.300 |
| C20.3n6 SNV | 3017~2823/1805~1734 | RidgeR | Milk | 0.507 | 1.426 | 0.567 | 1.523 |
| C20.3n6 SG | 3017~2823/1805~1734 | RidgeR | Milk | 0.451 | 1.352 | 0.469 | 1.375 |
| C20.3n6 MSC | 4000~400 | RidgeR | Fat | 0.350 | 1.234 | 0.211 | 1.124 |
| C20.3n6 DER1 | 4000~400 | RidgeR | Fat | 0.373 | 1.238 | 0.285 | 1.189 |
| C20.3n6 DER2 | 4000~400 | RidgeR | Fat | 0.596 | 1.453 | 0.306 | 1.207 |
| C20.3n6 SNV | 4000~400 | RidgeR | Fat | 0.345 | 1.225 | 0.208 | 1.126 |
| C20.3n6 SG | 4000~400 | RidgeR | Fat | 0.318 | 1.204 | 0.211 | 1.127 |
| C20.3n6 MSC | 4000~400 | RFR | Fat | 0.183 | 1.107 | 0.292 | 1.195 |
| C20.3n6 DER1 | 4000~400 | RFR | Fat | 0.172 | 1.099 | 0.319 | 1.216 |
| C20.3n6 DER2 | 4000~400 | RFR | Fat | 0.192 | 1.115 | 0.336 | 1.224 |
| C20.3n6 SNV | 4000~400 | RFR | Fat | 0.199 | 1.118 | 0.303 | 1.204 |
| C20.3n6 SG | 4000~400 | RFR | Fat | 0.179 | 1.104 | 0.315 | 1.215 |
| C20.3n6 MSC | 4000~400 | PLSR | Fat | 0.224 | 1.153 | 0.098 | 1.060 |
| C20.3n6 DER1 | 4000~400 | PLSR | Fat | 0.177 | 1.105 | 0.289 | 1.194 |
| C20.3n6 DER2 | 4000~400 | PLSR | Fat | 0.172 | 1.102 | 0.274 | 1.181 |
| C20.3n6 SNV | 4000~400 | PLSR | Fat | 0.212 | 1.146 | 0.065 | 1.041 |
| C20.3n6 SG | 4000~400 | PLSR | Fat | 0.181 | 1.111 | 0.066 | 1.041 |
| C20.3n6 MSC | 4000~400 | LassoR | Fat | 0.714 | 1.820 | 0.094 | 0.943 |
| C20.3n6 DER1 | 4000~400 | LassoR | Fat | 0.933 | 3.503 | 0.135 | 0.887 |
| C20.3n6 DER2 | 4000~400 | LassoR | Fat | 0.997 | 13.839 | 0.160 | 0.902 |
| C20.3n6 SNV | 4000~400 | LassoR | Fat | 0.719 | 1.836 | 0.097 | 0.946 |
| C20.3n6 SG | 4000~400 | LassoR | Fat | 0.620 | 1.593 | 0.099 | 0.971 |
| C20.3n6 MSC | 4000~400 | LassoR | Milk | 0.862 | 2.653 | 0.412 | 1.234 |
| C20.3n6 DER1 | 4000~400 | LassoR | Milk | 0.944 | 4.014 | 0.517 | 1.349 |
| C20.3n6 DER2 | 4000~400 | LassoR | Milk | 0.990 | 9.107 | 0.463 | 1.262 |
| C20.3n6 SNV | 4000~400 | LassoR | Milk | 0.865 | 2.689 | 0.426 | 1.263 |
| C20.3n6 SG | 4000~400 | LassoR | Milk | 0.805 | 2.244 | 0.435 | 1.277 |
| C20.3n6 MSC | 4000~400 | PLSR | Milk | 0.443 | 1.350 | 0.569 | 1.532 |
| C20.3n6 DER1 | 4000~400 | PLSR | Milk | 0.455 | 1.362 | 0.528 | 1.464 |
| C20.3n6 DER2 | 4000~400 | PLSR | Milk | 0.460 | 1.387 | 0.496 | 1.418 |
| C20.3n6 SNV | 4000~400 | PLSR | Milk | 0.516 | 1.444 | 0.598 | 1.587 |
| C20.3n6 SG | 4000~400 | PLSR | Milk | 0.438 | 1.345 | 0.572 | 1.539 |
| C20.3n6 MSC | 4000~400 | RFR | Milk | 0.476 | 1.384 | 0.592 | 1.565 |
| C20.3n6 DER1 | 4000~400 | RFR | Milk | 0.504 | 1.423 | 0.615 | 1.607 |
| C20.3n6 DER2 | 4000~400 | RFR | Milk | 0.473 | 1.380 | 0.602 | 1.573 |
| C20.3n6 SNV | 4000~400 | RFR | Milk | 0.488 | 1.400 | 0.612 | 1.594 |
| C20.3n6 SG | 4000~400 | RFR | Milk | 0.508 | 1.428 | 0.595 | 1.571 |
| C20.3n6 MSC | 4000~400 | RidgeR | Milk | 0.532 | 1.455 | 0.552 | 1.496 |
| C20.3n6 DER1 | 4000~400 | RidgeR | Milk | 0.617 | 1.593 | 0.563 | 1.517 |
| C20.3n6 DER2 | 4000~400 | RidgeR | Milk | 0.699 | 1.748 | 0.559 | 1.505 |
| C20.3n6 SNV | 4000~400 | RidgeR | Milk | 0.573 | 1.512 | 0.595 | 1.555 |
| C20.3n6 SG | 4000~400 | RidgeR | Milk | 0.519 | 1.438 | 0.516 | 1.446 |
| C20.4n6 MSC | 3017~2823/1805~1734 | LassoR | Fat | 0.308 | 1.204 | 0.449 | 1.353 |
| C20.4n6 DER1 | 3017~2823/1805~1734 | LassoR | Fat | 0.329 | 1.223 | 0.404 | 1.299 |
| C20.4n6 DER2 | 3017~2823/1805~1734 | LassoR | Fat | 0.334 | 1.228 | 0.382 | 1.273 |
| C20.4n6 SNV | 3017~2823/1805~1734 | LassoR | Fat | 0.391 | 1.283 | 0.397 | 1.284 |
| C20.4n6 SG | 3017~2823/1805~1734 | LassoR | Fat | 0.273 | 1.175 | 0.395 | 1.289 |
| C20.4n6 MSC | 3017~2823/1805~1734 | RidgeR | Fat | 0.298 | 1.195 | 0.441 | 1.341 |
| C20.4n6 DER1 | 3017~2823/1805~1734 | RidgeR | Fat | 0.307 | 1.203 | 0.405 | 1.300 |

| | | | | | | | |
|---|---|---|---|---|---|---|---|
| C20.4n6 DER2 | 3017~2823/1805~1734 | RidgeR | Fat | 0.303 | 1.199 | 0.399 | 1.294 |
| C20.4n6 SNV | 3017~2823/1805~1734 | RidgeR | Fat | 0.331 | 1.225 | 0.420 | 1.321 |
| C20.4n6 SG | 3017~2823/1805~1734 | RidgeR | Fat | 0.259 | 1.163 | 0.362 | 1.254 |
| C20.4n6 MSC | 3017~2823/1805~1734 | RFR | Fat | 0.243 | 1.138 | 0.382 | 1.241 |
| C20.4n6 DER1 | 3017~2823/1805~1734 | RFR | Fat | 0.271 | 1.160 | 0.305 | 1.163 |
| C20.4n6 DER2 | 3017~2823/1805~1734 | RFR | Fat | 0.283 | 1.173 | 0.296 | 1.169 |
| C20.4n6 SNV | 3017~2823/1805~1734 | RFR | Fat | 0.244 | 1.138 | 0.387 | 1.258 |
| C20.4n6 SG | 3017~2823/1805~1734 | RFR | Fat | 0.282 | 1.170 | 0.316 | 1.187 |
| C20.4n6 MSC | 3017~2823/1805~1734 | PLSR | Fat | 0.275 | 1.178 | 0.421 | 1.323 |
| C20.4n6 DER1 | 3017~2823/1805~1734 | PLSR | Fat | 0.237 | 1.151 | 0.391 | 1.290 |
| C20.4n6 DER2 | 3017~2823/1805~1734 | PLSR | Fat | 0.246 | 1.156 | 0.401 | 1.300 |
| C20.4n6 SNV | 3017~2823/1805~1734 | PLSR | Fat | 0.289 | 1.196 | 0.386 | 1.285 |
| C20.4n6 SG | 3017~2823/1805~1734 | PLSR | Fat | 0.232 | 1.145 | 0.325 | 1.225 |
| C20.4n6 MSC | 3017~2823/1805~1734 | LassoR | Milk | 0.474 | 1.382 | 0.463 | 1.371 |
| C20.4n6 DER1 | 3017~2823/1805~1734 | LassoR | Milk | 0.565 | 1.518 | 0.452 | 1.359 |
| C20.4n6 DER2 | 3017~2823/1805~1734 | LassoR | Milk | 0.505 | 1.424 | 0.451 | 1.356 |
| C20.4n6 SNV | 3017~2823/1805~1734 | LassoR | Milk | 0.472 | 1.379 | 0.462 | 1.371 |
| C20.4n6 SG | 3017~2823/1805~1734 | LassoR | Milk | 0.442 | 1.342 | 0.453 | 1.359 |
| C20.4n6 MSC | 3017~2823/1805~1734 | PLSR | Milk | 0.449 | 1.351 | 0.458 | 1.366 |
| C20.4n6 DER1 | 3017~2823/1805~1734 | PLSR | Milk | 0.427 | 1.326 | 0.469 | 1.381 |
| C20.4n6 DER2 | 3017~2823/1805~1734 | PLSR | Milk | 0.436 | 1.337 | 0.425 | 1.328 |
| C20.4n6 SNV | 3017~2823/1805~1734 | PLSR | Milk | 0.444 | 1.351 | 0.467 | 1.378 |
| C20.4n6 SG | 3017~2823/1805~1734 | PLSR | Milk | 0.423 | 1.321 | 0.450 | 1.357 |
| C20.4n6 MSC | 3017~2823/1805~1734 | RFR | Milk | 0.422 | 1.311 | 0.432 | 1.328 |
| C20.4n6 DER1 | 3017~2823/1805~1734 | RFR | Milk | 0.412 | 1.301 | 0.429 | 1.330 |
| C20.4n6 DER2 | 3017~2823/1805~1734 | RFR | Milk | 0.419 | 1.312 | 0.472 | 1.383 |
| C20.4n6 SNV | 3017~2823/1805~1734 | RFR | Milk | 0.404 | 1.290 | 0.456 | 1.360 |
| C20.4n6 SG | 3017~2823/1805~1734 | RFR | Milk | 0.441 | 1.333 | 0.489 | 1.403 |
| C20.4n6 MSC | 3017~2823/1805~1734 | RidgeR | Milk | 0.468 | 1.373 | 0.466 | 1.375 |
| C20.4n6 DER1 | 3017~2823/1805~1734 | RidgeR | Milk | 0.475 | 1.382 | 0.474 | 1.380 |
| C20.4n6 DER2 | 3017~2823/1805~1734 | RidgeR | Milk | 0.477 | 1.384 | 0.467 | 1.372 |
| C20.4n6 SNV | 3017~2823/1805~1734 | RidgeR | Milk | 0.468 | 1.374 | 0.465 | 1.374 |
| C20.4n6 SG | 3017~2823/1805~1734 | RidgeR | Milk | 0.442 | 1.341 | 0.456 | 1.362 |
| C20.4n6 MSC | 4000~400 | RidgeR | Fat | 0.392 | 1.275 | 0.450 | 1.351 |
| C20.4n6 DER1 | 4000~400 | RidgeR | Fat | 0.405 | 1.284 | 0.407 | 1.302 |
| C20.4n6 DER2 | 4000~400 | RidgeR | Fat | 0.456 | 1.330 | 0.391 | 1.286 |
| C20.4n6 SNV | 4000~400 | RidgeR | Fat | 0.405 | 1.287 | 0.443 | 1.344 |
| C20.4n6 SG | 4000~400 | RidgeR | Fat | 0.377 | 1.263 | 0.428 | 1.325 |
| C20.4n6 MSC | 4000~400 | RFR | Fat | 0.291 | 1.189 | 0.325 | 1.219 |
| C20.4n6 DER1 | 4000~400 | RFR | Fat | 0.321 | 1.215 | 0.383 | 1.275 |
| C20.4n6 DER2 | 4000~400 | RFR | Fat | 0.339 | 1.233 | 0.336 | 1.228 |
| C20.4n6 SNV | 4000~400 | RFR | Fat | 0.290 | 1.188 | 0.350 | 1.244 |
| C20.4n6 SG | 4000~400 | RFR | Fat | 0.302 | 1.198 | 0.322 | 1.200 |
| C20.4n6 MSC | 4000~400 | PLSR | Fat | 0.287 | 1.189 | 0.406 | 1.306 |
| C20.4n6 DER1 | 4000~400 | PLSR | Fat | 0.269 | 1.175 | 0.429 | 1.331 |
| C20.4n6 DER2 | 4000~400 | PLSR | Fat | 0.281 | 1.182 | 0.346 | 1.245 |
| C20.4n6 SNV | 4000~400 | PLSR | Fat | 0.288 | 1.191 | 0.457 | 1.365 |
| C20.4n6 SG | 4000~400 | PLSR | Fat | 0.282 | 1.185 | 0.419 | 1.320 |
| C20.4n6 MSC | 4000~400 | LassoR | Fat | 0.723 | 1.865 | 0.457 | 1.280 |
| C20.4n6 DER1 | 4000~400 | LassoR | Fat | 0.948 | 3.996 | 0.314 | 1.004 |

| | | | | | | | |
|---|---|---|---|---|---|---|---|
| C20.4n6 DER2 | 4000~400 | LassoR | Fat | 0.989 | 8.161 | 0.292 | 1.047 |
| C20.4n6 SNV | 4000~400 | LassoR | Fat | 0.730 | 1.890 | 0.449 | 1.273 |
| C20.4n6 SG | 4000~400 | LassoR | Fat | 0.687 | 1.766 | 0.453 | 1.278 |
| C20.4n6 MSC | 4000~400 | LassoR | Milk | 0.901 | 3.145 | 0.338 | 1.061 |
| C20.4n6 DER1 | 4000~400 | LassoR | Milk | 0.995 | 13.287 | 0.300 | 1.001 |
| C20.4n6 DER2 | 4000~400 | LassoR | Milk | 0.999 | 29.367 | 0.398 | 1.194 |
| C20.4n6 SNV | 4000~400 | LassoR | Milk | 0.905 | 3.195 | 0.327 | 1.042 |
| C20.4n6 SG | 4000~400 | LassoR | Milk | 0.861 | 2.662 | 0.319 | 1.077 |
| C20.4n6 MSC | 4000~400 | PLSR | Milk | 0.442 | 1.345 | 0.422 | 1.323 |
| C20.4n6 DER1 | 4000~400 | PLSR | Milk | 0.416 | 1.362 | 0.237 | 1.152 |
| C20.4n6 DER2 | 4000~400 | PLSR | Milk | 0.483 | 1.438 | 0.396 | 1.294 |
| C20.4n6 SNV | 4000~400 | PLSR | Milk | 0.454 | 1.360 | 0.437 | 1.342 |
| C20.4n6 SG | 4000~400 | PLSR | Milk | 0.433 | 1.332 | 0.396 | 1.294 |
| C20.4n6 MSC | 4000~400 | RFR | Milk | 0.456 | 1.357 | 0.468 | 1.380 |
| C20.4n6 DER1 | 4000~400 | RFR | Milk | 0.449 | 1.349 | 0.468 | 1.379 |
| C20.4n6 DER2 | 4000~400 | RFR | Milk | 0.436 | 1.334 | 0.483 | 1.395 |
| C20.4n6 SNV | 4000~400 | RFR | Milk | 0.443 | 1.343 | 0.497 | 1.419 |
| C20.4n6 SG | 4000~400 | RFR | Milk | 0.439 | 1.338 | 0.447 | 1.353 |
| C20.4n6 MSC | 4000~400 | RidgeR | Milk | 0.541 | 1.472 | 0.456 | 1.363 |
| C20.4n6 DER1 | 4000~400 | RidgeR | Milk | 0.612 | 1.590 | 0.489 | 1.404 |
| C20.4n6 DER2 | 4000~400 | RidgeR | Milk | 0.699 | 1.753 | 0.489 | 1.400 |
| C20.4n6 SNV | 4000~400 | RidgeR | Milk | 0.552 | 1.488 | 0.469 | 1.379 |
| C20.4n6 SG | 4000~400 | RidgeR | Milk | 0.521 | 1.441 | 0.452 | 1.357 |
| C20.5n3 MSC | 3017~2823/1805~1734 | LassoR | Fat | 0.829 | 2.426 | 0.878 | 2.720 |
| C20.5n3 DER1 | 3017~2823/1805~1734 | LassoR | Fat | 0.804 | 2.265 | 0.828 | 2.318 |
| C20.5n3 DER2 | 3017~2823/1805~1734 | LassoR | Fat | 0.854 | 2.622 | 0.776 | 2.008 |
| C20.5n3 SNV | 3017~2823/1805~1734 | LassoR | Fat | 0.837 | 2.482 | 0.894 | 2.876 |
| C20.5n3 SG | 3017~2823/1805~1734 | LassoR | Fat | 0.792 | 2.199 | 0.830 | 2.356 |
| C20.5n3 MSC | 3017~2823/1805~1734 | RidgeR | Fat | 0.821 | 2.368 | 0.874 | 2.673 |
| C20.5n3 DER1 | 3017~2823/1805~1734 | RidgeR | Fat | 0.788 | 2.175 | 0.824 | 2.312 |
| C20.5n3 DER2 | 3017~2823/1805~1734 | RidgeR | Fat | 0.790 | 2.183 | 0.824 | 2.312 |
| C20.5n3 SNV | 3017~2823/1805~1734 | RidgeR | Fat | 0.829 | 2.422 | 0.888 | 2.805 |
| C20.5n3 SG | 3017~2823/1805~1734 | RidgeR | Fat | 0.790 | 2.187 | 0.827 | 2.342 |
| C20.5n3 MSC | 3017~2823/1805~1734 | RFR | Fat | 0.794 | 2.204 | 0.885 | 2.685 |
| C20.5n3 DER1 | 3017~2823/1805~1734 | RFR | Fat | 0.821 | 2.367 | 0.892 | 2.748 |
| C20.5n3 DER2 | 3017~2823/1805~1734 | RFR | Fat | 0.815 | 2.323 | 0.879 | 2.545 |
| C20.5n3 SNV | 3017~2823/1805~1734 | RFR | Fat | 0.799 | 2.230 | 0.898 | 2.793 |
| C20.5n3 SG | 3017~2823/1805~1734 | RFR | Fat | 0.805 | 2.267 | 0.888 | 2.769 |
| C20.5n3 MSC | 3017~2823/1805~1734 | PLSR | Fat | 0.817 | 2.348 | 0.863 | 2.715 |
| C20.5n3 DER1 | 3017~2823/1805~1734 | PLSR | Fat | 0.797 | 2.248 | 0.797 | 2.231 |
| C20.5n3 DER2 | 3017~2823/1805~1734 | PLSR | Fat | 0.788 | 2.194 | 0.743 | 1.986 |
| C20.5n3 SNV | 3017~2823/1805~1734 | PLSR | Fat | 0.822 | 2.378 | 0.875 | 2.844 |
| C20.5n3 SG | 3017~2823/1805~1734 | PLSR | Fat | 0.792 | 2.224 | 0.794 | 2.215 |
| C20.5n3 MSC | 3017~2823/1805~1734 | LassoR | Milk | 0.399 | 1.293 | 0.389 | 1.248 |
| C20.5n3 DER1 | 3017~2823/1805~1734 | LassoR | Milk | 0.412 | 1.306 | 0.343 | 1.185 |
| C20.5n3 DER2 | 3017~2823/1805~1734 | LassoR | Milk | 0.462 | 1.366 | 0.306 | 1.134 |
| C20.5n3 SNV | 3017~2823/1805~1734 | LassoR | Milk | 0.395 | 1.288 | 0.360 | 1.215 |
| C20.5n3 SG | 3017~2823/1805~1734 | LassoR | Milk | 0.391 | 1.284 | 0.344 | 1.184 |
| C20.5n3 MSC | 3017~2823/1805~1734 | PLSR | Milk | 0.353 | 1.247 | 0.333 | 1.232 |
| C20.5n3 DER1 | 3017~2823/1805~1734 | PLSR | Milk | 0.351 | 1.245 | 0.270 | 1.178 |

| | | | | | | | |
|---|---|---|---|---|---|---|---|
| C20.5n3 DER2 | 3017~2823/1805~1734 | PLSR | Milk | 0.333 | 1.227 | 0.285 | 1.190 |
| C20.5n3 SNV | 3017~2823/1805~1734 | PLSR | Milk | 0.346 | 1.241 | 0.302 | 1.204 |
| C20.5n3 SG | 3017~2823/1805~1734 | PLSR | Milk | 0.356 | 1.250 | 0.267 | 1.175 |
| C20.5n3 MSC | 3017~2823/1805~1734 | RFR | Milk | 0.306 | 1.192 | 0.310 | 1.150 |
| C20.5n3 DER1 | 3017~2823/1805~1734 | RFR | Milk | 0.302 | 1.190 | 0.264 | 1.118 |
| C20.5n3 DER2 | 3017~2823/1805~1734 | RFR | Milk | 0.280 | 1.167 | 0.324 | 1.181 |
| C20.5n3 SNV | 3017~2823/1805~1734 | RFR | Milk | 0.293 | 1.179 | 0.338 | 1.185 |
| C20.5n3 SG | 3017~2823/1805~1734 | RFR | Milk | 0.301 | 1.186 | 0.262 | 1.098 |
| C20.5n3 MSC | 3017~2823/1805~1734 | RidgeR | Milk | 0.380 | 1.273 | 0.391 | 1.254 |
| C20.5n3 DER1 | 3017~2823/1805~1734 | RidgeR | Milk | 0.396 | 1.289 | 0.352 | 1.198 |
| C20.5n3 DER2 | 3017~2823/1805~1734 | RidgeR | Milk | 0.407 | 1.299 | 0.356 | 1.204 |
| C20.5n3 SNV | 3017~2823/1805~1734 | RidgeR | Milk | 0.384 | 1.276 | 0.368 | 1.226 |
| C20.5n3 SG | 3017~2823/1805~1734 | RidgeR | Milk | 0.387 | 1.279 | 0.348 | 1.190 |
| C20.5n3 MSC | 4000~400 | RidgeR | Fat | 0.809 | 2.288 | 0.837 | 2.409 |
| C20.5n3 DER1 | 4000~400 | RidgeR | Fat | 0.828 | 2.397 | 0.828 | 2.373 |
| C20.5n3 DER2 | 4000~400 | RidgeR | Fat | 0.855 | 2.591 | 0.831 | 2.386 |
| C20.5n3 SNV | 4000~400 | RidgeR | Fat | 0.819 | 2.338 | 0.855 | 2.549 |
| C20.5n3 SG | 4000~400 | RidgeR | Fat | 0.793 | 2.195 | 0.817 | 2.284 |
| C20.5n3 MSC | 4000~400 | RFR | Fat | 0.812 | 2.312 | 0.898 | 2.892 |
| C20.5n3 DER1 | 4000~400 | RFR | Fat | 0.828 | 2.414 | 0.909 | 3.061 |
| C20.5n3 DER2 | 4000~400 | RFR | Fat | 0.825 | 2.391 | 0.891 | 2.768 |
| C20.5n3 SNV | 4000~400 | RFR | Fat | 0.815 | 2.327 | 0.900 | 2.811 |
| C20.5n3 SG | 4000~400 | RFR | Fat | 0.828 | 2.410 | 0.899 | 2.880 |
| C20.5n3 MSC | 4000~400 | PLSR | Fat | 0.779 | 2.133 | 0.825 | 2.405 |
| C20.5n3 DER1 | 4000~400 | PLSR | Fat | 0.778 | 2.128 | 0.818 | 2.359 |
| C20.5n3 DER2 | 4000~400 | PLSR | Fat | 0.759 | 2.098 | 0.823 | 2.389 |
| C20.5n3 SNV | 4000~400 | PLSR | Fat | 0.783 | 2.167 | 0.859 | 2.675 |
| C20.5n3 SG | 4000~400 | PLSR | Fat | 0.766 | 2.078 | 0.821 | 2.376 |
| C20.5n3 MSC | 4000~400 | LassoR | Fat | 0.932 | 3.832 | 0.760 | 1.917 |
| C20.5n3 DER1 | 4000~400 | LassoR | Fat | 0.988 | 9.008 | 0.691 | 1.692 |
| C20.5n3 DER2 | 4000~400 | LassoR | Fat | 0.999 | 34.321 | 0.745 | 1.841 |
| C20.5n3 SNV | 4000~400 | LassoR | Fat | 0.950 | 4.479 | 0.805 | 2.176 |
| C20.5n3 SG | 4000~400 | LassoR | Fat | 0.899 | 3.147 | 0.766 | 1.916 |
| C20.5n3 MSC | 4000~400 | LassoR | Milk | 0.750 | 1.963 | 0.397 | 1.229 |
| C20.5n3 DER1 | 4000~400 | LassoR | Milk | 0.962 | 4.806 | 0.306 | 1.013 |
| C20.5n3 DER2 | 4000~400 | LassoR | Milk | 0.994 | 11.721 | 0.281 | 0.950 |
| C20.5n3 SNV | 4000~400 | LassoR | Milk | 0.753 | 1.971 | 0.402 | 1.239 |
| C20.5n3 SG | 4000~400 | LassoR | Milk | 0.685 | 1.762 | 0.410 | 1.237 |
| C20.5n3 MSC | 4000~400 | PLSR | Milk | 0.323 | 1.219 | 0.366 | 1.263 |
| C20.5n3 DER1 | 4000~400 | PLSR | Milk | 0.333 | 1.229 | 0.291 | 1.194 |
| C20.5n3 DER2 | 4000~400 | PLSR | Milk | 0.328 | 1.223 | 0.317 | 1.217 |
| C20.5n3 SNV | 4000~400 | PLSR | Milk | 0.328 | 1.223 | 0.372 | 1.270 |
| C20.5n3 SG | 4000~400 | PLSR | Milk | 0.328 | 1.224 | 0.326 | 1.226 |
| C20.5n3 MSC | 4000~400 | RFR | Milk | 0.344 | 1.234 | 0.329 | 1.186 |
| C20.5n3 DER1 | 4000~400 | RFR | Milk | 0.303 | 1.197 | 0.388 | 1.253 |
| C20.5n3 DER2 | 4000~400 | RFR | Milk | 0.333 | 1.226 | 0.427 | 1.292 |
| C20.5n3 SNV | 4000~400 | RFR | Milk | 0.336 | 1.224 | 0.319 | 1.155 |
| C20.5n3 SG | 4000~400 | RFR | Milk | 0.302 | 1.194 | 0.357 | 1.210 |
| C20.5n3 MSC | 4000~400 | RidgeR | Milk | 0.430 | 1.320 | 0.408 | 1.270 |
| C20.5n3 DER1 | 4000~400 | RidgeR | Milk | 0.542 | 1.455 | 0.407 | 1.275 |

| | | | | | | | | |
|---|---|---|---|---|---|---|---|---|
| C20.5n3 | DER2 | 4000~400 | RidgeR | Milk | 0.609 | 1.541 | 0.393 | 1.251 |
| C20.5n3 | SNV | 4000~400 | RidgeR | Milk | 0.418 | 1.307 | 0.404 | 1.263 |
| C20.5n3 | SG | 4000~400 | RidgeR | Milk | 0.402 | 1.291 | 0.380 | 1.241 |
| C22.0 | MSC | 3017~2823/1805~1734 | LassoR | Fat | 0.836 | 2.474 | 0.830 | 2.433 |
| C22.0 | DER1 | 3017~2823/1805~1734 | LassoR | Fat | 0.847 | 2.560 | 0.844 | 2.523 |
| C22.0 | DER2 | 3017~2823/1805~1734 | LassoR | Fat | 0.843 | 2.528 | 0.828 | 2.390 |
| C22.0 | SNV | 3017~2823/1805~1734 | LassoR | Fat | 0.836 | 2.478 | 0.835 | 2.472 |
| C22.0 | SG | 3017~2823/1805~1734 | LassoR | Fat | 0.778 | 2.128 | 0.778 | 2.084 |
| C22.0 | MSC | 3017~2823/1805~1734 | RidgeR | Fat | 0.809 | 2.296 | 0.813 | 2.316 |
| C22.0 | DER1 | 3017~2823/1805~1734 | RidgeR | Fat | 0.769 | 2.085 | 0.797 | 2.149 |
| C22.0 | DER2 | 3017~2823/1805~1734 | RidgeR | Fat | 0.775 | 2.111 | 0.806 | 2.189 |
| C22.0 | SNV | 3017~2823/1805~1734 | RidgeR | Fat | 0.821 | 2.370 | 0.828 | 2.424 |
| C22.0 | SG | 3017~2823/1805~1734 | RidgeR | Fat | 0.754 | 2.022 | 0.785 | 2.092 |
| C22.0 | MSC | 3017~2823/1805~1734 | RFR | Fat | 0.801 | 2.247 | 0.820 | 2.359 |
| C22.0 | DER1 | 3017~2823/1805~1734 | RFR | Fat | 0.805 | 2.265 | 0.832 | 2.433 |
| C22.0 | DER2 | 3017~2823/1805~1734 | RFR | Fat | 0.811 | 2.306 | 0.838 | 2.492 |
| C22.0 | SNV | 3017~2823/1805~1734 | RFR | Fat | 0.797 | 2.226 | 0.820 | 2.353 |
| C22.0 | SG | 3017~2823/1805~1734 | RFR | Fat | 0.812 | 2.312 | 0.844 | 2.501 |
| C22.0 | MSC | 3017~2823/1805~1734 | PLSR | Fat | 0.812 | 2.317 | 0.825 | 2.405 |
| C22.0 | DER1 | 3017~2823/1805~1734 | PLSR | Fat | 0.771 | 2.116 | 0.845 | 2.559 |
| C22.0 | DER2 | 3017~2823/1805~1734 | PLSR | Fat | 0.775 | 2.134 | 0.825 | 2.408 |
| C22.0 | SNV | 3017~2823/1805~1734 | PLSR | Fat | 0.832 | 2.455 | 0.846 | 2.564 |
| C22.0 | SG | 3017~2823/1805~1734 | PLSR | Fat | 0.772 | 2.109 | 0.807 | 2.292 |
| C22.0 | MSC | 3017~2823/1805~1734 | LassoR | Milk | 0.560 | 1.510 | 0.453 | 1.334 |
| C22.0 | DER1 | 3017~2823/1805~1734 | LassoR | Milk | 0.598 | 1.578 | 0.470 | 1.357 |
| C22.0 | DER2 | 3017~2823/1805~1734 | LassoR | Milk | 0.621 | 1.627 | 0.484 | 1.379 |
| C22.0 | SNV | 3017~2823/1805~1734 | LassoR | Milk | 0.566 | 1.518 | 0.461 | 1.346 |
| C22.0 | SG | 3017~2823/1805~1734 | LassoR | Milk | 0.562 | 1.513 | 0.385 | 1.247 |
| C22.0 | MSC | 3017~2823/1805~1734 | PLSR | Milk | 0.483 | 1.400 | 0.462 | 1.372 |
| C22.0 | DER1 | 3017~2823/1805~1734 | PLSR | Milk | 0.478 | 1.395 | 0.435 | 1.339 |
| C22.0 | DER2 | 3017~2823/1805~1734 | PLSR | Milk | 0.452 | 1.359 | 0.444 | 1.349 |
| C22.0 | SNV | 3017~2823/1805~1734 | PLSR | Milk | 0.477 | 1.393 | 0.473 | 1.386 |
| C22.0 | SG | 3017~2823/1805~1734 | PLSR | Milk | 0.524 | 1.457 | 0.370 | 1.267 |
| C22.0 | MSC | 3017~2823/1805~1734 | RFR | Milk | 0.353 | 1.237 | 0.399 | 1.283 |
| C22.0 | DER1 | 3017~2823/1805~1734 | RFR | Milk | 0.333 | 1.226 | 0.362 | 1.244 |
| C22.0 | DER2 | 3017~2823/1805~1734 | RFR | Milk | 0.419 | 1.314 | 0.455 | 1.334 |
| C22.0 | SNV | 3017~2823/1805~1734 | RFR | Milk | 0.342 | 1.230 | 0.398 | 1.281 |
| C22.0 | SG | 3017~2823/1805~1734 | RFR | Milk | 0.378 | 1.268 | 0.380 | 1.257 |
| C22.0 | MSC | 3017~2823/1805~1734 | RidgeR | Milk | 0.432 | 1.329 | 0.367 | 1.246 |
| C22.0 | DER1 | 3017~2823/1805~1734 | RidgeR | Milk | 0.507 | 1.419 | 0.413 | 1.300 |
| C22.0 | DER2 | 3017~2823/1805~1734 | RidgeR | Milk | 0.552 | 1.493 | 0.455 | 1.344 |
| C22.0 | SNV | 3017~2823/1805~1734 | RidgeR | Milk | 0.422 | 1.318 | 0.359 | 1.237 |
| C22.0 | SG | 3017~2823/1805~1734 | RidgeR | Milk | 0.405 | 1.299 | 0.329 | 1.214 |
| C22.0 | MSC | 4000~400 | RidgeR | Fat | 0.806 | 2.248 | 0.807 | 2.196 |
| C22.0 | DER1 | 4000~400 | RidgeR | Fat | 0.818 | 2.331 | 0.808 | 2.178 |
| C22.0 | DER2 | 4000~400 | RidgeR | Fat | 0.850 | 2.549 | 0.806 | 2.159 |
| C22.0 | SNV | 4000~400 | RidgeR | Fat | 0.819 | 2.332 | 0.822 | 2.304 |
| C22.0 | SG | 4000~400 | RidgeR | Fat | 0.787 | 2.168 | 0.801 | 2.165 |
| C22.0 | MSC | 4000~400 | RFR | Fat | 0.817 | 2.334 | 0.830 | 2.441 |
| C22.0 | DER1 | 4000~400 | RFR | Fat | 0.823 | 2.378 | 0.842 | 2.490 |

| | | | | | | | | |
|---|---|---|---|---|---|---|---|---|
| C22.0 | DER2 | 4000~400 | RFR | Fat | 0.830 | 2.422 | 0.861 | 2.660 |
| C22.0 | SNV | 4000~400 | RFR | Fat | 0.813 | 2.313 | 0.831 | 2.444 |
| C22.0 | SG | 4000~400 | RFR | Fat | 0.818 | 2.347 | 0.837 | 2.434 |
| C22.0 | MSC | 4000~400 | PLSR | Fat | 0.781 | 2.144 | 0.797 | 2.234 |
| C22.0 | DER1 | 4000~400 | PLSR | Fat | 0.761 | 2.051 | 0.794 | 2.219 |
| C22.0 | DER2 | 4000~400 | PLSR | Fat | 0.754 | 2.092 | 0.734 | 1.952 |
| C22.0 | SNV | 4000~400 | PLSR | Fat | 0.796 | 2.227 | 0.847 | 2.570 |
| C22.0 | SG | 4000~400 | PLSR | Fat | 0.756 | 2.035 | 0.777 | 2.131 |
| C22.0 | MSC | 4000~400 | LassoR | Fat | 0.943 | 4.185 | 0.719 | 1.896 |
| C22.0 | DER1 | 4000~400 | LassoR | Fat | 0.995 | 13.262 | 0.662 | 1.711 |
| C22.0 | DER2 | 4000~400 | LassoR | Fat | 0.997 | 18.463 | 0.708 | 1.845 |
| C22.0 | SNV | 4000~400 | LassoR | Fat | 0.948 | 4.358 | 0.783 | 2.159 |
| C22.0 | SG | 4000~400 | LassoR | Fat | 0.914 | 3.401 | 0.720 | 1.902 |
| C22.0 | MSC | 4000~400 | LassoR | Milk | 0.844 | 2.484 | 0.359 | 1.184 |
| C22.0 | DER1 | 4000~400 | LassoR | Milk | 0.959 | 4.634 | 0.378 | 1.203 |
| C22.0 | DER2 | 4000~400 | LassoR | Milk | 0.995 | 12.277 | 0.448 | 1.257 |
| C22.0 | SNV | 4000~400 | LassoR | Milk | 0.855 | 2.584 | 0.349 | 1.164 |
| C22.0 | SG | 4000~400 | LassoR | Milk | 0.800 | 2.212 | 0.367 | 1.202 |
| C22.0 | MSC | 4000~400 | PLSR | Milk | 0.402 | 1.298 | 0.386 | 1.284 |
| C22.0 | DER1 | 4000~400 | PLSR | Milk | 0.419 | 1.319 | 0.418 | 1.319 |
| C22.0 | DER2 | 4000~400 | PLSR | Milk | 0.442 | 1.350 | 0.494 | 1.414 |
| C22.0 | SNV | 4000~400 | PLSR | Milk | 0.410 | 1.310 | 0.411 | 1.310 |
| C22.0 | SG | 4000~400 | PLSR | Milk | 0.403 | 1.300 | 0.334 | 1.232 |
| C22.0 | MSC | 4000~400 | RFR | Milk | 0.401 | 1.294 | 0.368 | 1.256 |
| C22.0 | DER1 | 4000~400 | RFR | Milk | 0.419 | 1.314 | 0.512 | 1.420 |
| C22.0 | DER2 | 4000~400 | RFR | Milk | 0.444 | 1.337 | 0.451 | 1.340 |
| C22.0 | SNV | 4000~400 | RFR | Milk | 0.394 | 1.283 | 0.381 | 1.270 |
| C22.0 | SG | 4000~400 | RFR | Milk | 0.400 | 1.293 | 0.442 | 1.335 |
| C22.0 | MSC | 4000~400 | RidgeR | Milk | 0.503 | 1.414 | 0.397 | 1.288 |
| C22.0 | DER1 | 4000~400 | RidgeR | Milk | 0.602 | 1.558 | 0.459 | 1.358 |
| C22.0 | DER2 | 4000~400 | RidgeR | Milk | 0.702 | 1.758 | 0.522 | 1.443 |
| C22.0 | SNV | 4000~400 | RidgeR | Milk | 0.524 | 1.445 | 0.396 | 1.284 |
| C22.0 | SG | 4000~400 | RidgeR | Milk | 0.498 | 1.407 | 0.383 | 1.274 |
| C22.1n9 | MSC | 3017~2823/1805~1734 | LassoR | Fat | 0.504 | 1.418 | 0.550 | 1.478 |
| C22.1n9 | DER1 | 3017~2823/1805~1734 | LassoR | Fat | 0.582 | 1.548 | 0.489 | 1.365 |
| C22.1n9 | DER2 | 3017~2823/1805~1734 | LassoR | Fat | 0.662 | 1.721 | 0.519 | 1.416 |
| C22.1n9 | SNV | 3017~2823/1805~1734 | LassoR | Fat | 0.570 | 1.527 | 0.569 | 1.479 |
| C22.1n9 | SG | 3017~2823/1805~1734 | LassoR | Fat | 0.468 | 1.371 | 0.465 | 1.343 |
| C22.1n9 | MSC | 3017~2823/1805~1734 | RidgeR | Fat | 0.341 | 1.234 | 0.480 | 1.389 |
| C22.1n9 | DER1 | 3017~2823/1805~1734 | RidgeR | Fat | 0.491 | 1.397 | 0.512 | 1.428 |
| C22.1n9 | DER2 | 3017~2823/1805~1734 | RidgeR | Fat | 0.553 | 1.494 | 0.532 | 1.452 |
| C22.1n9 | SNV | 3017~2823/1805~1734 | RidgeR | Fat | 0.341 | 1.234 | 0.476 | 1.383 |
| C22.1n9 | SG | 3017~2823/1805~1734 | RidgeR | Fat | 0.329 | 1.223 | 0.437 | 1.333 |
| C22.1n9 | MSC | 3017~2823/1805~1734 | RFR | Fat | 0.293 | 1.181 | 0.415 | 1.277 |
| C22.1n9 | DER1 | 3017~2823/1805~1734 | RFR | Fat | 0.404 | 1.297 | 0.483 | 1.388 |
| C22.1n9 | DER2 | 3017~2823/1805~1734 | RFR | Fat | 0.514 | 1.437 | 0.616 | 1.595 |
| C22.1n9 | SNV | 3017~2823/1805~1734 | RFR | Fat | 0.309 | 1.199 | 0.412 | 1.255 |
| C22.1n9 | SG | 3017~2823/1805~1734 | RFR | Fat | 0.288 | 1.175 | 0.437 | 1.311 |
| C22.1n9 | MSC | 3017~2823/1805~1734 | PLSR | Fat | 0.440 | 1.343 | 0.486 | 1.404 |
| C22.1n9 | DER1 | 3017~2823/1805~1734 | PLSR | Fat | 0.476 | 1.393 | 0.421 | 1.322 |

| | | | | | | | |
|---|---|---|---|---|---|---|---|
| C22.1n9 DER2 | 3017~2823/1805~1734 | PLSR | Fat | 0.488 | 1.415 | 0.477 | 1.392 |
| C22.1n9 SNV | 3017~2823/1805~1734 | PLSR | Fat | 0.461 | 1.369 | 0.507 | 1.433 |
| C22.1n9 SG | 3017~2823/1805~1734 | PLSR | Fat | 0.410 | 1.310 | 0.402 | 1.301 |
| C22.1n9 MSC | 3017~2823/1805~1734 | LassoR | Milk | 0.402 | 1.289 | 0.409 | 1.300 |
| C22.1n9 DER1 | 3017~2823/1805~1734 | LassoR | Milk | 0.450 | 1.347 | 0.362 | 1.256 |
| C22.1n9 DER2 | 3017~2823/1805~1734 | LassoR | Milk | 0.565 | 1.517 | 0.298 | 1.154 |
| C22.1n9 SNV | 3017~2823/1805~1734 | LassoR | Milk | 0.381 | 1.265 | 0.419 | 1.307 |
| C22.1n9 SG | 3017~2823/1805~1734 | LassoR | Milk | 0.325 | 1.210 | 0.313 | 1.212 |
| C22.1n9 MSC | 3017~2823/1805~1734 | PLSR | Milk | 0.309 | 1.208 | 0.370 | 1.268 |
| C22.1n9 DER1 | 3017~2823/1805~1734 | PLSR | Milk | 0.346 | 1.246 | 0.279 | 1.185 |
| C22.1n9 DER2 | 3017~2823/1805~1734 | PLSR | Milk | 0.351 | 1.252 | 0.224 | 1.142 |
| C22.1n9 SNV | 3017~2823/1805~1734 | PLSR | Milk | 0.300 | 1.203 | 0.423 | 1.325 |
| C22.1n9 SG | 3017~2823/1805~1734 | PLSR | Milk | 0.293 | 1.198 | 0.362 | 1.260 |
| C22.1n9 MSC | 3017~2823/1805~1734 | RFR | Milk | 0.137 | 1.064 | 0.177 | 1.098 |
| C22.1n9 DER1 | 3017~2823/1805~1734 | RFR | Milk | 0.300 | 1.197 | 0.176 | 1.101 |
| C22.1n9 DER2 | 3017~2823/1805~1734 | RFR | Milk | 0.325 | 1.218 | 0.371 | 1.256 |
| C22.1n9 SNV | 3017~2823/1805~1734 | RFR | Milk | 0.140 | 1.070 | 0.244 | 1.153 |
| C22.1n9 SG | 3017~2823/1805~1734 | RFR | Milk | 0.118 | 1.048 | 0.320 | 1.214 |
| C22.1n9 MSC | 3017~2823/1805~1734 | RidgeR | Milk | 0.206 | 1.124 | 0.180 | 1.108 |
| C22.1n9 DER1 | 3017~2823/1805~1734 | RidgeR | Milk | 0.387 | 1.266 | 0.364 | 1.256 |
| C22.1n9 DER2 | 3017~2823/1805~1734 | RidgeR | Milk | 0.465 | 1.364 | 0.331 | 1.225 |
| C22.1n9 SNV | 3017~2823/1805~1734 | RidgeR | Milk | 0.208 | 1.125 | 0.190 | 1.116 |
| C22.1n9 SG | 3017~2823/1805~1734 | RidgeR | Milk | 0.170 | 1.099 | 0.169 | 1.101 |
| C22.1n9 MSC | 4000~400 | RidgeR | Fat | 0.601 | 1.561 | 0.593 | 1.568 |
| C22.1n9 DER1 | 4000~400 | RidgeR | Fat | 0.652 | 1.671 | 0.595 | 1.567 |
| C22.1n9 DER2 | 4000~400 | RidgeR | Fat | 0.689 | 1.755 | 0.570 | 1.513 |
| C22.1n9 SNV | 4000~400 | RidgeR | Fat | 0.604 | 1.566 | 0.606 | 1.593 |
| C22.1n9 SG | 4000~400 | RidgeR | Fat | 0.581 | 1.525 | 0.571 | 1.529 |
| C22.1n9 MSC | 4000~400 | RFR | Fat | 0.494 | 1.408 | 0.619 | 1.574 |
| C22.1n9 DER1 | 4000~400 | RFR | Fat | 0.529 | 1.449 | 0.648 | 1.673 |
| C22.1n9 DER2 | 4000~400 | RFR | Fat | 0.557 | 1.495 | 0.607 | 1.586 |
| C22.1n9 SNV | 4000~400 | RFR | Fat | 0.449 | 1.349 | 0.600 | 1.550 |
| C22.1n9 SG | 4000~400 | RFR | Fat | 0.472 | 1.376 | 0.564 | 1.493 |
| C22.1n9 MSC | 4000~400 | PLSR | Fat | 0.516 | 1.444 | 0.560 | 1.517 |
| C22.1n9 DER1 | 4000~400 | PLSR | Fat | 0.516 | 1.443 | 0.554 | 1.507 |
| C22.1n9 DER2 | 4000~400 | PLSR | Fat | 0.524 | 1.455 | 0.538 | 1.480 |
| C22.1n9 SNV | 4000~400 | PLSR | Fat | 0.518 | 1.448 | 0.566 | 1.528 |
| C22.1n9 SG | 4000~400 | PLSR | Fat | 0.512 | 1.439 | 0.533 | 1.473 |
| C22.1n9 MSC | 4000~400 | LassoR | Fat | 0.839 | 2.464 | 0.496 | 1.309 |
| C22.1n9 DER1 | 4000~400 | LassoR | Fat | 0.965 | 5.088 | 0.418 | 1.109 |
| C22.1n9 DER2 | 4000~400 | LassoR | Fat | 0.997 | 17.301 | 0.330 | 1.010 |
| C22.1n9 SNV | 4000~400 | LassoR | Fat | 0.840 | 2.475 | 0.502 | 1.328 |
| C22.1n9 SG | 4000~400 | LassoR | Fat | 0.788 | 2.159 | 0.491 | 1.318 |
| C22.1n9 MSC | 4000~400 | LassoR | Milk | 0.810 | 2.264 | 0.200 | 1.020 |
| C22.1n9 DER1 | 4000~400 | LassoR | Milk | 0.944 | 3.973 | 0.188 | 0.966 |
| C22.1n9 DER2 | 4000~400 | LassoR | Milk | 0.994 | 11.008 | 0.194 | 0.955 |
| C22.1n9 SNV | 4000~400 | LassoR | Milk | 0.814 | 2.283 | 0.185 | 1.006 |
| C22.1n9 SG | 4000~400 | LassoR | Milk | 0.732 | 1.918 | 0.221 | 1.032 |
| C22.1n9 MSC | 4000~400 | PLSR | Milk | 0.431 | 1.332 | 0.335 | 1.234 |
| C22.1n9 DER1 | 4000~400 | PLSR | Milk | 0.397 | 1.294 | 0.352 | 1.250 |

| | | | | | | | | |
|---|---|---|---|---|---|---|---|---|
| C22.1n9 | DER2 | 4000~400 | PLSR | Milk | 0.426 | 1.324 | 0.410 | 1.310 |
| C22.1n9 | SNV | 4000~400 | PLSR | Milk | 0.430 | 1.330 | 0.337 | 1.236 |
| C22.1n9 | SG | 4000~400 | PLSR | Milk | 0.422 | 1.322 | 0.340 | 1.239 |
| C22.1n9 | MSC | 4000~400 | RFR | Milk | 0.424 | 1.319 | 0.407 | 1.307 |
| C22.1n9 | DER1 | 4000~400 | RFR | Milk | 0.502 | 1.413 | 0.436 | 1.336 |
| C22.1n9 | DER2 | 4000~400 | RFR | Milk | 0.511 | 1.428 | 0.453 | 1.360 |
| C22.1n9 | SNV | 4000~400 | RFR | Milk | 0.415 | 1.305 | 0.374 | 1.258 |
| C22.1n9 | SG | 4000~400 | RFR | Milk | 0.415 | 1.309 | 0.397 | 1.295 |
| C22.1n9 | MSC | 4000~400 | RidgeR | Milk | 0.520 | 1.410 | 0.365 | 1.263 |
| C22.1n9 | DER1 | 4000~400 | RidgeR | Milk | 0.577 | 1.503 | 0.404 | 1.303 |
| C22.1n9 | DER2 | 4000~400 | RidgeR | Milk | 0.595 | 1.530 | 0.417 | 1.317 |
| C22.1n9 | SNV | 4000~400 | RidgeR | Milk | 0.520 | 1.412 | 0.364 | 1.262 |
| C22.1n9 | SG | 4000~400 | RidgeR | Milk | 0.498 | 1.378 | 0.361 | 1.258 |
| C24.0 | MSC | 3017~2823/1805~1734 | LassoR | Fat | 0.896 | 3.105 | 0.761 | 1.990 |
| C24.0 | DER1 | 3017~2823/1805~1734 | LassoR | Fat | 0.893 | 3.069 | 0.715 | 1.813 |
| C24.0 | DER2 | 3017~2823/1805~1734 | LassoR | Fat | 0.912 | 3.378 | 0.736 | 1.858 |
| C24.0 | SNV | 3017~2823/1805~1734 | LassoR | Fat | 0.909 | 3.321 | 0.792 | 2.152 |
| C24.0 | SG | 3017~2823/1805~1734 | LassoR | Fat | 0.851 | 2.599 | 0.705 | 1.786 |
| C24.0 | MSC | 3017~2823/1805~1734 | RidgeR | Fat | 0.893 | 3.058 | 0.755 | 1.963 |
| C24.0 | DER1 | 3017~2823/1805~1734 | RidgeR | Fat | 0.856 | 2.637 | 0.705 | 1.790 |
| C24.0 | DER2 | 3017~2823/1805~1734 | RidgeR | Fat | 0.855 | 2.626 | 0.710 | 1.809 |
| C24.0 | SNV | 3017~2823/1805~1734 | RidgeR | Fat | 0.901 | 3.182 | 0.776 | 2.071 |
| C24.0 | SG | 3017~2823/1805~1734 | RidgeR | Fat | 0.850 | 2.587 | 0.704 | 1.790 |
| C24.0 | MSC | 3017~2823/1805~1734 | RFR | Fat | 0.889 | 3.004 | 0.776 | 2.065 |
| C24.0 | DER1 | 3017~2823/1805~1734 | RFR | Fat | 0.898 | 3.125 | 0.780 | 2.066 |
| C24.0 | DER2 | 3017~2823/1805~1734 | RFR | Fat | 0.906 | 3.261 | 0.788 | 2.121 |
| C24.0 | SNV | 3017~2823/1805~1734 | RFR | Fat | 0.888 | 2.984 | 0.769 | 2.015 |
| C24.0 | SG | 3017~2823/1805~1734 | RFR | Fat | 0.897 | 3.123 | 0.782 | 2.106 |
| C24.0 | MSC | 3017~2823/1805~1734 | PLSR | Fat | 0.888 | 2.996 | 0.735 | 1.955 |
| C24.0 | DER1 | 3017~2823/1805~1734 | PLSR | Fat | 0.869 | 2.799 | 0.736 | 1.958 |
| C24.0 | DER2 | 3017~2823/1805~1734 | PLSR | Fat | 0.865 | 2.763 | 0.711 | 1.870 |
| C24.0 | SNV | 3017~2823/1805~1734 | PLSR | Fat | 0.902 | 3.216 | 0.789 | 2.189 |
| C24.0 | SG | 3017~2823/1805~1734 | PLSR | Fat | 0.865 | 2.746 | 0.738 | 1.966 |
| C24.0 | MSC | 3017~2823/1805~1734 | LassoR | Milk | 0.480 | 1.387 | 0.591 | 1.547 |
| C24.0 | DER1 | 3017~2823/1805~1734 | LassoR | Milk | 0.543 | 1.480 | 0.540 | 1.477 |
| C24.0 | DER2 | 3017~2823/1805~1734 | LassoR | Milk | 0.547 | 1.486 | 0.588 | 1.557 |
| C24.0 | SNV | 3017~2823/1805~1734 | LassoR | Milk | 0.483 | 1.392 | 0.587 | 1.543 |
| C24.0 | SG | 3017~2823/1805~1734 | LassoR | Milk | 0.419 | 1.312 | 0.576 | 1.465 |
| C24.0 | MSC | 3017~2823/1805~1734 | PLSR | Milk | 0.388 | 1.286 | 0.549 | 1.498 |
| C24.0 | DER1 | 3017~2823/1805~1734 | PLSR | Milk | 0.363 | 1.265 | 0.519 | 1.451 |
| C24.0 | DER2 | 3017~2823/1805~1734 | PLSR | Milk | 0.386 | 1.288 | 0.572 | 1.538 |
| C24.0 | SNV | 3017~2823/1805~1734 | PLSR | Milk | 0.385 | 1.283 | 0.550 | 1.500 |
| C24.0 | SG | 3017~2823/1805~1734 | PLSR | Milk | 0.373 | 1.273 | 0.564 | 1.524 |
| C24.0 | MSC | 3017~2823/1805~1734 | RFR | Milk | 0.265 | 1.158 | 0.336 | 1.230 |
| C24.0 | DER1 | 3017~2823/1805~1734 | RFR | Milk | 0.256 | 1.155 | 0.322 | 1.217 |
| C24.0 | DER2 | 3017~2823/1805~1734 | RFR | Milk | 0.281 | 1.180 | 0.475 | 1.361 |
| C24.0 | SNV | 3017~2823/1805~1734 | RFR | Milk | 0.256 | 1.152 | 0.345 | 1.238 |
| C24.0 | SG | 3017~2823/1805~1734 | RFR | Milk | 0.260 | 1.153 | 0.356 | 1.236 |
| C24.0 | MSC | 3017~2823/1805~1734 | RidgeR | Milk | 0.342 | 1.234 | 0.511 | 1.386 |
| C24.0 | DER1 | 3017~2823/1805~1734 | RidgeR | Milk | 0.394 | 1.279 | 0.546 | 1.407 |

| | | | | | | | | |
|---|---|---|---|---|---|---|---|---|
| C24.0 | DER2 | 3017~2823/1805~1734 | RidgeR | Milk | 0.444 | 1.340 | 0.553 | 1.455 |
| C24.0 | SNV | 3017~2823/1805~1734 | RidgeR | Milk | 0.342 | 1.235 | 0.458 | 1.352 |
| C24.0 | SG | 3017~2823/1805~1734 | RidgeR | Milk | 0.290 | 1.189 | 0.462 | 1.315 |
| C24.0 | MSC | 4000~400 | RidgeR | Fat | 0.874 | 2.791 | 0.721 | 1.884 |
| C24.0 | DER1 | 4000~400 | RidgeR | Fat | 0.880 | 2.859 | 0.713 | 1.853 |
| C24.0 | DER2 | 4000~400 | RidgeR | Fat | 0.901 | 3.121 | 0.715 | 1.857 |
| C24.0 | SNV | 4000~400 | RidgeR | Fat | 0.885 | 2.910 | 0.731 | 1.928 |
| C24.0 | SG | 4000~400 | RidgeR | Fat | 0.857 | 2.644 | 0.716 | 1.849 |
| C24.0 | MSC | 4000~400 | RFR | Fat | 0.891 | 3.037 | 0.788 | 2.141 |
| C24.0 | DER1 | 4000~400 | RFR | Fat | 0.904 | 3.230 | 0.795 | 2.193 |
| C24.0 | DER2 | 4000~400 | RFR | Fat | 0.910 | 3.340 | 0.781 | 2.084 |
| C24.0 | SNV | 4000~400 | RFR | Fat | 0.892 | 3.052 | 0.794 | 2.177 |
| C24.0 | SG | 4000~400 | RFR | Fat | 0.902 | 3.198 | 0.796 | 2.199 |
| C24.0 | MSC | 4000~400 | PLSR | Fat | 0.853 | 2.615 | 0.701 | 1.840 |
| C24.0 | DER1 | 4000~400 | PLSR | Fat | 0.842 | 2.529 | 0.691 | 1.809 |
| C24.0 | DER2 | 4000~400 | PLSR | Fat | 0.828 | 2.722 | 0.693 | 1.816 |
| C24.0 | SNV | 4000~400 | PLSR | Fat | 0.877 | 2.873 | 0.702 | 1.844 |
| C24.0 | SG | 4000~400 | PLSR | Fat | 0.838 | 2.497 | 0.692 | 1.814 |
| C24.0 | MSC | 4000~400 | LassoR | Fat | 0.958 | 4.869 | 0.669 | 1.677 |
| C24.0 | DER1 | 4000~400 | LassoR | Fat | 0.997 | 16.875 | 0.645 | 1.564 |
| C24.0 | DER2 | 4000~400 | LassoR | Fat | 0.999 | 40.771 | 0.692 | 1.717 |
| C24.0 | SNV | 4000~400 | LassoR | Fat | 0.969 | 5.639 | 0.685 | 1.730 |
| C24.0 | SG | 4000~400 | LassoR | Fat | 0.928 | 3.727 | 0.673 | 1.660 |
| C24.0 | MSC | 4000~400 | LassoR | Milk | 0.724 | 1.857 | 0.331 | 1.218 |
| C24.0 | DER1 | 4000~400 | LassoR | Milk | 0.956 | 4.372 | 0.294 | 1.129 |
| C24.0 | DER2 | 4000~400 | LassoR | Milk | 0.989 | 7.973 | 0.484 | 1.376 |
| C24.0 | SNV | 4000~400 | LassoR | Milk | 0.736 | 1.905 | 0.361 | 1.250 |
| C24.0 | SG | 4000~400 | LassoR | Milk | 0.668 | 1.704 | 0.359 | 1.254 |
| C24.0 | MSC | 4000~400 | PLSR | Milk | 0.251 | 1.158 | 0.418 | 1.319 |
| C24.0 | DER1 | 4000~400 | PLSR | Milk | 0.241 | 1.154 | 0.427 | 1.329 |
| C24.0 | DER2 | 4000~400 | PLSR | Milk | 0.251 | 1.164 | 0.545 | 1.492 |
| C24.0 | SNV | 4000~400 | PLSR | Milk | 0.306 | 1.205 | 0.403 | 1.303 |
| C24.0 | SG | 4000~400 | PLSR | Milk | 0.258 | 1.165 | 0.405 | 1.305 |
| C24.0 | MSC | 4000~400 | RFR | Milk | 0.286 | 1.184 | 0.497 | 1.382 |
| C24.0 | DER1 | 4000~400 | RFR | Milk | 0.298 | 1.195 | 0.527 | 1.401 |
| C24.0 | DER2 | 4000~400 | RFR | Milk | 0.311 | 1.207 | 0.499 | 1.354 |
| C24.0 | SNV | 4000~400 | RFR | Milk | 0.274 | 1.172 | 0.458 | 1.340 |
| C24.0 | SG | 4000~400 | RFR | Milk | 0.293 | 1.191 | 0.483 | 1.358 |
| C24.0 | MSC | 4000~400 | RidgeR | Milk | 0.340 | 1.229 | 0.475 | 1.321 |
| C24.0 | DER1 | 4000~400 | RidgeR | Milk | 0.499 | 1.380 | 0.521 | 1.367 |
| C24.0 | DER2 | 4000~400 | RidgeR | Milk | 0.636 | 1.552 | 0.607 | 1.456 |
| C24.0 | SNV | 4000~400 | RidgeR | Milk | 0.409 | 1.297 | 0.451 | 1.329 |
| C24.0 | SG | 4000~400 | RidgeR | Milk | 0.333 | 1.222 | 0.448 | 1.295 |
| C8.0 | MSC | 3017~2823/1805~1734 | LassoR | Fat | 0.431 | 1.327 | 0.430 | 1.317 |
| C8.0 | DER1 | 3017~2823/1805~1734 | LassoR | Fat | 0.450 | 1.348 | 0.414 | 1.306 |
| C8.0 | DER2 | 3017~2823/1805~1734 | LassoR | Fat | 0.409 | 1.296 | 0.342 | 1.229 |
| C8.0 | SNV | 3017~2823/1805~1734 | LassoR | Fat | 0.430 | 1.326 | 0.427 | 1.313 |
| C8.0 | SG | 3017~2823/1805~1734 | LassoR | Fat | 0.392 | 1.280 | 0.401 | 1.282 |
| C8.0 | MSC | 3017~2823/1805~1734 | RidgeR | Fat | 0.341 | 1.225 | 0.383 | 1.241 |
| C8.0 | DER1 | 3017~2823/1805~1734 | RidgeR | Fat | 0.344 | 1.230 | 0.348 | 1.233 |

| | | | | | | | | |
|---|---|---|---|---|---|---|---|---|
| C8.0 | DER2 | 3017~2823/1805~1734 | RidgeR | Fat | 0.362 | 1.248 | 0.345 | 1.227 |
| C8.0 | SNV | 3017~2823/1805~1734 | RidgeR | Fat | 0.332 | 1.216 | 0.364 | 1.221 |
| C8.0 | SG | 3017~2823/1805~1734 | RidgeR | Fat | 0.255 | 1.147 | 0.248 | 1.139 |
| C8.0 | MSC | 3017~2823/1805~1734 | RFR | Fat | 0.116 | 1.048 | 0.100 | 1.038 |
| C8.0 | DER1 | 3017~2823/1805~1734 | RFR | Fat | 0.087 | 1.034 | 0.155 | 1.077 |
| C8.0 | DER2 | 3017~2823/1805~1734 | RFR | Fat | 0.120 | 1.065 | 0.173 | 1.093 |
| C8.0 | SNV | 3017~2823/1805~1734 | RFR | Fat | 0.112 | 1.050 | 0.126 | 1.047 |
| C8.0 | SG | 3017~2823/1805~1734 | RFR | Fat | 0.056 | 1.007 | 0.050 | 0.995 |
| C8.0 | MSC | 3017~2823/1805~1734 | PLSR | Fat | 0.354 | 1.249 | 0.402 | 1.301 |
| C8.0 | DER1 | 3017~2823/1805~1734 | PLSR | Fat | 0.274 | 1.185 | 0.389 | 1.287 |
| C8.0 | DER2 | 3017~2823/1805~1734 | PLSR | Fat | 0.226 | 1.148 | 0.276 | 1.183 |
| C8.0 | SNV | 3017~2823/1805~1734 | PLSR | Fat | 0.340 | 1.237 | 0.409 | 1.309 |
| C8.0 | SG | 3017~2823/1805~1734 | PLSR | Fat | 0.338 | 1.236 | 0.385 | 1.283 |
| C8.0 | MSC | 3017~2823/1805~1734 | LassoR | Milk | 0.778 | 2.128 | 0.766 | 2.060 |
| C8.0 | DER1 | 3017~2823/1805~1734 | LassoR | Milk | 0.794 | 2.206 | 0.702 | 1.764 |
| C8.0 | DER2 | 3017~2823/1805~1734 | LassoR | Milk | 0.799 | 2.234 | 0.721 | 1.853 |
| C8.0 | SNV | 3017~2823/1805~1734 | LassoR | Milk | 0.783 | 2.153 | 0.767 | 2.065 |
| C8.0 | SG | 3017~2823/1805~1734 | LassoR | Milk | 0.772 | 2.099 | 0.711 | 1.820 |
| C8.0 | MSC | 3017~2823/1805~1734 | PLSR | Milk | 0.749 | 2.004 | 0.753 | 2.023 |
| C8.0 | DER1 | 3017~2823/1805~1734 | PLSR | Milk | 0.739 | 1.972 | 0.656 | 1.716 |
| C8.0 | DER2 | 3017~2823/1805~1734 | PLSR | Milk | 0.720 | 1.909 | 0.687 | 1.799 |
| C8.0 | SNV | 3017~2823/1805~1734 | PLSR | Milk | 0.751 | 2.010 | 0.773 | 2.113 |
| C8.0 | SG | 3017~2823/1805~1734 | PLSR | Milk | 0.744 | 1.991 | 0.670 | 1.751 |
| C8.0 | MSC | 3017~2823/1805~1734 | RFR | Milk | 0.644 | 1.675 | 0.739 | 1.940 |
| C8.0 | DER1 | 3017~2823/1805~1734 | RFR | Milk | 0.666 | 1.730 | 0.744 | 1.917 |
| C8.0 | DER2 | 3017~2823/1805~1734 | RFR | Milk | 0.667 | 1.735 | 0.749 | 1.924 |
| C8.0 | SNV | 3017~2823/1805~1734 | RFR | Milk | 0.643 | 1.676 | 0.732 | 1.921 |
| C8.0 | SG | 3017~2823/1805~1734 | RFR | Milk | 0.635 | 1.655 | 0.750 | 1.941 |
| C8.0 | MSC | 3017~2823/1805~1734 | RidgeR | Milk | 0.736 | 1.949 | 0.764 | 2.043 |
| C8.0 | DER1 | 3017~2823/1805~1734 | RidgeR | Milk | 0.752 | 2.009 | 0.683 | 1.741 |
| C8.0 | DER2 | 3017~2823/1805~1734 | RidgeR | Milk | 0.765 | 2.064 | 0.693 | 1.774 |
| C8.0 | SNV | 3017~2823/1805~1734 | RidgeR | Milk | 0.733 | 1.938 | 0.760 | 2.020 |
| C8.0 | SG | 3017~2823/1805~1734 | RidgeR | Milk | 0.700 | 1.828 | 0.655 | 1.667 |
| C8.0 | MSC | 4000~400 | RidgeR | Fat | 0.449 | 1.240 | 0.219 | 1.113 |
| C8.0 | DER1 | 4000~400 | RidgeR | Fat | 0.483 | 1.286 | 0.197 | 1.101 |
| C8.0 | DER2 | 4000~400 | RidgeR | Fat | 0.616 | 1.376 | 0.235 | 1.123 |
| C8.0 | SNV | 4000~400 | RidgeR | Fat | 0.438 | 1.233 | 0.214 | 1.110 |
| C8.0 | SG | 4000~400 | RidgeR | Fat | 0.330 | 1.173 | 0.148 | 1.076 |
| C8.0 | MSC | 4000~400 | RFR | Fat | 0.089 | 1.046 | 0.243 | 1.123 |
| C8.0 | DER1 | 4000~400 | RFR | Fat | 0.111 | 1.063 | 0.250 | 1.123 |
| C8.0 | DER2 | 4000~400 | RFR | Fat | 0.078 | 1.043 | 0.141 | 1.076 |
| C8.0 | SNV | 4000~400 | RFR | Fat | 0.069 | 1.034 | 0.158 | 1.089 |
| C8.0 | SG | 4000~400 | RFR | Fat | 0.059 | 1.020 | 0.135 | 1.079 |
| C8.0 | MSC | 4000~400 | PLSR | Fat | 0.096 | 1.073 | 0.063 | 1.039 |
| C8.0 | DER1 | 4000~400 | PLSR | Fat | 0.100 | 1.075 | 0.064 | 1.040 |
| C8.0 | DER2 | 4000~400 | PLSR | Fat | 0.035 | 1.041 | 0.227 | 1.145 |
| C8.0 | SNV | 4000~400 | PLSR | Fat | 0.126 | 1.086 | 0.146 | 1.089 |
| C8.0 | SG | 4000~400 | PLSR | Fat | -0.015 | 1.010 | 0.006 | 1.009 |
| C8.0 | MSC | 4000~400 | LassoR | Fat | 0.819 | 2.301 | 0.188 | 1.021 |
| C8.0 | DER1 | 4000~400 | LassoR | Fat | 0.926 | 3.335 | 0.205 | 1.020 |

| | | | | | | | | |
|---|---|---|---|---|---|---|---|---|
| C8.0 | DER2 | 4000~400 | LassoR | Fat | 0.987 | 7.265 | 0.246 | 1.098 |
| C8.0 | SNV | 4000~400 | LassoR | Fat | 0.825 | 2.338 | 0.192 | 1.016 |
| C8.0 | SG | 4000~400 | LassoR | Fat | 0.770 | 2.053 | 0.196 | 1.034 |
| C8.0 | MSC | 4000~400 | LassoR | Milk | 0.944 | 4.200 | 0.655 | 1.659 |
| C8.0 | DER1 | 4000~400 | LassoR | Milk | 0.990 | 10.010 | 0.598 | 1.483 |
| C8.0 | DER2 | 4000~400 | LassoR | Milk | 0.999 | 37.059 | 0.668 | 1.691 |
| C8.0 | SNV | 4000~400 | LassoR | Milk | 0.934 | 3.869 | 0.682 | 1.751 |
| C8.0 | SG | 4000~400 | LassoR | Milk | 0.910 | 3.325 | 0.682 | 1.743 |
| C8.0 | MSC | 4000~400 | PLSR | Milk | 0.704 | 1.887 | 0.742 | 1.981 |
| C8.0 | DER1 | 4000~400 | PLSR | Milk | 0.670 | 1.749 | 0.719 | 1.898 |
| C8.0 | DER2 | 4000~400 | PLSR | Milk | 0.663 | 1.766 | 0.713 | 1.878 |
| C8.0 | SNV | 4000~400 | PLSR | Milk | 0.709 | 1.901 | 0.735 | 1.954 |
| C8.0 | SG | 4000~400 | PLSR | Milk | 0.660 | 1.722 | 0.617 | 1.627 |
| C8.0 | MSC | 4000~400 | RFR | Milk | 0.647 | 1.687 | 0.727 | 1.864 |
| C8.0 | DER1 | 4000~400 | RFR | Milk | 0.687 | 1.791 | 0.739 | 1.903 |
| C8.0 | DER2 | 4000~400 | RFR | Milk | 0.676 | 1.760 | 0.767 | 1.992 |
| C8.0 | SNV | 4000~400 | RFR | Milk | 0.651 | 1.696 | 0.720 | 1.847 |
| C8.0 | SG | 4000~400 | RFR | Milk | 0.655 | 1.705 | 0.764 | 1.949 |
| C8.0 | MSC | 4000~400 | RidgeR | Milk | 0.718 | 1.872 | 0.702 | 1.785 |
| C8.0 | DER1 | 4000~400 | RidgeR | Milk | 0.754 | 2.004 | 0.696 | 1.775 |
| C8.0 | DER2 | 4000~400 | RidgeR | Milk | 0.797 | 2.177 | 0.693 | 1.763 |
| C8.0 | SNV | 4000~400 | RidgeR | Milk | 0.728 | 1.902 | 0.719 | 1.834 |
| C8.0 | SG | 4000~400 | RidgeR | Milk | 0.707 | 1.846 | 0.667 | 1.697 |
| LCFA | MSC | 3017~2823/1805~1734 | LassoR | Fat | 0.539 | 1.473 | 0.331 | 1.213 |
| LCFA | DER1 | 3017~2823/1805~1734 | LassoR | Fat | 0.538 | 1.473 | 0.317 | 1.196 |
| LCFA | DER2 | 3017~2823/1805~1734 | LassoR | Fat | 0.600 | 1.583 | 0.313 | 1.180 |
| LCFA | SNV | 3017~2823/1805~1734 | LassoR | Fat | 0.547 | 1.488 | 0.328 | 1.208 |
| LCFA | SG | 3017~2823/1805~1734 | LassoR | Fat | 0.514 | 1.437 | 0.298 | 1.159 |
| LCFA | MSC | 3017~2823/1805~1734 | RidgeR | Fat | 0.435 | 1.332 | 0.287 | 1.173 |
| LCFA | DER1 | 3017~2823/1805~1734 | RidgeR | Fat | 0.476 | 1.376 | 0.317 | 1.205 |
| LCFA | DER2 | 3017~2823/1805~1734 | RidgeR | Fat | 0.520 | 1.440 | 0.335 | 1.220 |
| LCFA | SNV | 3017~2823/1805~1734 | RidgeR | Fat | 0.438 | 1.335 | 0.291 | 1.177 |
| LCFA | SG | 3017~2823/1805~1734 | RidgeR | Fat | 0.432 | 1.327 | 0.272 | 1.150 |
| LCFA | MSC | 3017~2823/1805~1734 | RFR | Fat | 0.285 | 1.178 | 0.299 | 1.171 |
| LCFA | DER1 | 3017~2823/1805~1734 | RFR | Fat | 0.252 | 1.158 | 0.259 | 1.155 |
| LCFA | DER2 | 3017~2823/1805~1734 | RFR | Fat | 0.276 | 1.177 | 0.325 | 1.208 |
| LCFA | SNV | 3017~2823/1805~1734 | RFR | Fat | 0.274 | 1.170 | 0.281 | 1.159 |
| LCFA | SG | 3017~2823/1805~1734 | RFR | Fat | 0.281 | 1.178 | 0.315 | 1.178 |
| LCFA | MSC | 3017~2823/1805~1734 | PLSR | Fat | 0.422 | 1.329 | 0.336 | 1.235 |
| LCFA | DER1 | 3017~2823/1805~1734 | PLSR | Fat | 0.378 | 1.283 | 0.359 | 1.257 |
| LCFA | DER2 | 3017~2823/1805~1734 | PLSR | Fat | 0.428 | 1.338 | 0.210 | 1.132 |
| LCFA | SNV | 3017~2823/1805~1734 | PLSR | Fat | 0.419 | 1.326 | 0.315 | 1.216 |
| LCFA | SG | 3017~2823/1805~1734 | PLSR | Fat | 0.439 | 1.343 | 0.296 | 1.199 |
| LCFA | MSC | 3017~2823/1805~1734 | LassoR | Milk | 0.664 | 1.728 | 0.790 | 2.114 |
| LCFA | DER1 | 3017~2823/1805~1734 | LassoR | Milk | 0.686 | 1.786 | 0.828 | 2.314 |
| LCFA | DER2 | 3017~2823/1805~1734 | LassoR | Milk | 0.731 | 1.932 | 0.820 | 2.278 |
| LCFA | SNV | 3017~2823/1805~1734 | LassoR | Milk | 0.671 | 1.746 | 0.800 | 2.163 |
| LCFA | SG | 3017~2823/1805~1734 | LassoR | Milk | 0.660 | 1.718 | 0.826 | 2.312 |
| LCFA | MSC | 3017~2823/1805~1734 | PLSR | Milk | 0.592 | 1.580 | 0.763 | 2.066 |
| LCFA | DER1 | 3017~2823/1805~1734 | PLSR | Milk | 0.623 | 1.638 | 0.808 | 2.299 |

| | | | | | | | | | |
|---|---|---|---|---|---|---|---|---|---|
| LCFA | DER2 | 3017~2823/1805~1734 | PLSR | Milk | 0.632 | 1.666 | 0.798 | 2.241 |
| LCFA | SNV | 3017~2823/1805~1734 | PLSR | Milk | 0.615 | 1.625 | 0.773 | 2.111 |
| LCFA | SG | 3017~2823/1805~1734 | PLSR | Milk | 0.641 | 1.680 | 0.827 | 2.422 |
| LCFA | MSC | 3017~2823/1805~1734 | RFR | Milk | 0.596 | 1.575 | 0.619 | 1.605 |
| LCFA | DER1 | 3017~2823/1805~1734 | RFR | Milk | 0.608 | 1.599 | 0.733 | 1.914 |
| LCFA | DER2 | 3017~2823/1805~1734 | RFR | Milk | 0.636 | 1.660 | 0.759 | 1.987 |
| LCFA | SNV | 3017~2823/1805~1734 | RFR | Milk | 0.599 | 1.579 | 0.690 | 1.787 |
| LCFA | SG | 3017~2823/1805~1734 | RFR | Milk | 0.626 | 1.637 | 0.702 | 1.806 |
| LCFA | MSC | 3017~2823/1805~1734 | RidgeR | Milk | 0.633 | 1.653 | 0.750 | 1.954 |
| LCFA | DER1 | 3017~2823/1805~1734 | RidgeR | Milk | 0.659 | 1.713 | 0.825 | 2.249 |
| LCFA | DER2 | 3017~2823/1805~1734 | RidgeR | Milk | 0.683 | 1.778 | 0.833 | 2.292 |
| LCFA | SNV | 3017~2823/1805~1734 | RidgeR | Milk | 0.638 | 1.666 | 0.758 | 1.984 |
| LCFA | SG | 3017~2823/1805~1734 | RidgeR | Milk | 0.649 | 1.692 | 0.816 | 2.240 |
| LCFA | MSC | 4000~400 | RidgeR | Fat | 0.499 | 1.396 | 0.286 | 1.180 |
| LCFA | DER1 | 4000~400 | RidgeR | Fat | 0.623 | 1.592 | 0.356 | 1.246 |
| LCFA | DER2 | 4000~400 | RidgeR | Fat | 0.654 | 1.635 | 0.368 | 1.258 |
| LCFA | SNV | 4000~400 | RidgeR | Fat | 0.499 | 1.398 | 0.288 | 1.183 |
| LCFA | SG | 4000~400 | RidgeR | Fat | 0.494 | 1.390 | 0.277 | 1.169 |
| LCFA | MSC | 4000~400 | RFR | Fat | 0.380 | 1.271 | 0.321 | 1.216 |
| LCFA | DER1 | 4000~400 | RFR | Fat | 0.414 | 1.306 | 0.422 | 1.315 |
| LCFA | DER2 | 4000~400 | RFR | Fat | 0.392 | 1.282 | 0.402 | 1.275 |
| LCFA | SNV | 4000~400 | RFR | Fat | 0.361 | 1.253 | 0.290 | 1.182 |
| LCFA | SG | 4000~400 | RFR | Fat | 0.340 | 1.232 | 0.287 | 1.185 |
| LCFA | MSC | 4000~400 | PLSR | Fat | 0.376 | 1.280 | 0.235 | 1.150 |
| LCFA | DER1 | 4000~400 | PLSR | Fat | 0.434 | 1.337 | 0.275 | 1.182 |
| LCFA | DER2 | 4000~400 | PLSR | Fat | 0.403 | 1.303 | 0.303 | 1.205 |
| LCFA | SNV | 4000~400 | PLSR | Fat | 0.371 | 1.266 | 0.246 | 1.159 |
| LCFA | SG | 4000~400 | PLSR | Fat | 0.383 | 1.283 | 0.227 | 1.144 |
| LCFA | MSC | 4000~400 | LassoR | Fat | 0.823 | 2.345 | 0.238 | 1.084 |
| LCFA | DER1 | 4000~400 | LassoR | Fat | 0.946 | 4.095 | 0.234 | 1.080 |
| LCFA | DER2 | 4000~400 | LassoR | Fat | 0.994 | 11.300 | 0.252 | 1.066 |
| LCFA | SNV | 4000~400 | LassoR | Fat | 0.821 | 2.331 | 0.243 | 1.089 |
| LCFA | SG | 4000~400 | LassoR | Fat | 0.775 | 2.090 | 0.301 | 1.139 |
| LCFA | MSC | 4000~400 | LassoR | Milk | 0.900 | 3.128 | 0.553 | 1.468 |
| LCFA | DER1 | 4000~400 | LassoR | Milk | 0.988 | 8.997 | 0.480 | 1.286 |
| LCFA | DER2 | 4000~400 | LassoR | Milk | 0.999 | 30.446 | 0.523 | 1.405 |
| LCFA | SNV | 4000~400 | LassoR | Milk | 0.897 | 3.089 | 0.563 | 1.487 |
| LCFA | SG | 4000~400 | LassoR | Milk | 0.854 | 2.602 | 0.660 | 1.702 |
| LCFA | MSC | 4000~400 | PLSR | Milk | 0.617 | 1.622 | 0.736 | 1.959 |
| LCFA | DER1 | 4000~400 | PLSR | Milk | 0.643 | 1.717 | 0.653 | 1.709 |
| LCFA | DER2 | 4000~400 | PLSR | Milk | 0.616 | 1.664 | 0.675 | 1.765 |
| LCFA | SNV | 4000~400 | PLSR | Milk | 0.618 | 1.623 | 0.736 | 1.958 |
| LCFA | SG | 4000~400 | PLSR | Milk | 0.615 | 1.619 | 0.770 | 2.098 |
| LCFA | MSC | 4000~400 | RFR | Milk | 0.616 | 1.616 | 0.721 | 1.859 |
| LCFA | DER1 | 4000~400 | RFR | Milk | 0.651 | 1.695 | 0.771 | 2.039 |
| LCFA | DER2 | 4000~400 | RFR | Milk | 0.644 | 1.673 | 0.765 | 2.012 |
| LCFA | SNV | 4000~400 | RFR | Milk | 0.614 | 1.612 | 0.710 | 1.850 |
| LCFA | SG | 4000~400 | RFR | Milk | 0.605 | 1.593 | 0.744 | 1.971 |
| LCFA | MSC | 4000~400 | RidgeR | Milk | 0.675 | 1.753 | 0.767 | 1.987 |
| LCFA | DER1 | 4000~400 | RidgeR | Milk | 0.739 | 1.946 | 0.802 | 2.184 |

| | | | | | | | | |
|---|---|---|---|---|---|---|---|---|
| LCFA | DER2 | 4000~400 | RidgeR | Milk | 0.778 | 2.062 | 0.766 | 1.981 |
| LCFA | SNV | 4000~400 | RidgeR | Milk | 0.677 | 1.757 | 0.768 | 1.994 |
| LCFA | SG | 4000~400 | RidgeR | Milk | 0.671 | 1.742 | 0.800 | 2.128 |
| MCFA | MSC | 3017~2823/1805~1734 | LassoR | Fat | 0.220 | 1.133 | 0.260 | 1.162 |
| MCFA | DER1 | 3017~2823/1805~1734 | LassoR | Fat | 0.249 | 1.153 | 0.235 | 1.144 |
| MCFA | DER2 | 3017~2823/1805~1734 | LassoR | Fat | 0.224 | 1.134 | 0.188 | 1.111 |
| MCFA | SNV | 3017~2823/1805~1734 | LassoR | Fat | 0.226 | 1.137 | 0.283 | 1.179 |
| MCFA | SG | 3017~2823/1805~1734 | LassoR | Fat | 0.259 | 1.158 | 0.262 | 1.164 |
| MCFA | MSC | 3017~2823/1805~1734 | RidgeR | Fat | 0.143 | 1.076 | 0.168 | 1.096 |
| MCFA | DER1 | 3017~2823/1805~1734 | RidgeR | Fat | 0.196 | 1.111 | 0.219 | 1.131 |
| MCFA | DER2 | 3017~2823/1805~1734 | RidgeR | Fat | 0.173 | 1.096 | 0.176 | 1.103 |
| MCFA | SNV | 3017~2823/1805~1734 | RidgeR | Fat | 0.129 | 1.068 | 0.179 | 1.100 |
| MCFA | SG | 3017~2823/1805~1734 | RidgeR | Fat | 0.054 | 1.029 | 0.105 | 1.053 |
| MCFA | MSC | 3017~2823/1805~1734 | RFR | Fat | 0.058 | 1.005 | 0.069 | 0.998 |
| MCFA | DER1 | 3017~2823/1805~1734 | RFR | Fat | 0.041 | 1.004 | 0.100 | 1.028 |
| MCFA | DER2 | 3017~2823/1805~1734 | RFR | Fat | 0.044 | 1.010 | 0.094 | 1.014 |
| MCFA | SNV | 3017~2823/1805~1734 | RFR | Fat | 0.049 | 0.997 | 0.032 | 0.959 |
| MCFA | SG | 3017~2823/1805~1734 | RFR | Fat | 0.012 | 0.965 | 0.049 | 0.973 |
| MCFA | MSC | 3017~2823/1805~1734 | PLSR | Fat | 0.150 | 1.089 | 0.243 | 1.157 |
| MCFA | DER1 | 3017~2823/1805~1734 | PLSR | Fat | 0.097 | 1.065 | 0.206 | 1.129 |
| MCFA | DER2 | 3017~2823/1805~1734 | PLSR | Fat | 0.070 | 1.042 | 0.154 | 1.094 |
| MCFA | SNV | 3017~2823/1805~1734 | PLSR | Fat | 0.146 | 1.086 | 0.269 | 1.177 |
| MCFA | SG | 3017~2823/1805~1734 | PLSR | Fat | 0.166 | 1.101 | 0.214 | 1.135 |
| MCFA | MSC | 3017~2823/1805~1734 | LassoR | Milk | 0.734 | 1.942 | 0.628 | 1.583 |
| MCFA | DER1 | 3017~2823/1805~1734 | LassoR | Milk | 0.751 | 2.009 | 0.573 | 1.395 |
| MCFA | DER2 | 3017~2823/1805~1734 | LassoR | Milk | 0.733 | 1.939 | 0.606 | 1.485 |
| MCFA | SNV | 3017~2823/1805~1734 | LassoR | Milk | 0.734 | 1.944 | 0.630 | 1.588 |
| MCFA | SG | 3017~2823/1805~1734 | LassoR | Milk | 0.693 | 1.809 | 0.616 | 1.517 |
| MCFA | MSC | 3017~2823/1805~1734 | PLSR | Milk | 0.691 | 1.807 | 0.591 | 1.574 |
| MCFA | DER1 | 3017~2823/1805~1734 | PLSR | Milk | 0.690 | 1.803 | 0.530 | 1.468 |
| MCFA | DER2 | 3017~2823/1805~1734 | PLSR | Milk | 0.684 | 1.786 | 0.547 | 1.495 |
| MCFA | SNV | 3017~2823/1805~1734 | PLSR | Milk | 0.690 | 1.806 | 0.598 | 1.588 |
| MCFA | SG | 3017~2823/1805~1734 | PLSR | Milk | 0.688 | 1.817 | 0.501 | 1.424 |
| MCFA | MSC | 3017~2823/1805~1734 | RFR | Milk | 0.652 | 1.697 | 0.704 | 1.828 |
| MCFA | DER1 | 3017~2823/1805~1734 | RFR | Milk | 0.651 | 1.695 | 0.736 | 1.928 |
| MCFA | DER2 | 3017~2823/1805~1734 | RFR | Milk | 0.641 | 1.668 | 0.750 | 2.000 |
| MCFA | SNV | 3017~2823/1805~1734 | RFR | Milk | 0.638 | 1.664 | 0.701 | 1.826 |
| MCFA | SG | 3017~2823/1805~1734 | RFR | Milk | 0.632 | 1.649 | 0.746 | 1.959 |
| MCFA | MSC | 3017~2823/1805~1734 | RidgeR | Milk | 0.678 | 1.766 | 0.695 | 1.772 |
| MCFA | DER1 | 3017~2823/1805~1734 | RidgeR | Milk | 0.706 | 1.848 | 0.621 | 1.529 |
| MCFA | DER2 | 3017~2823/1805~1734 | RidgeR | Milk | 0.708 | 1.853 | 0.624 | 1.540 |
| MCFA | SNV | 3017~2823/1805~1734 | RidgeR | Milk | 0.680 | 1.772 | 0.684 | 1.735 |
| MCFA | SG | 3017~2823/1805~1734 | RidgeR | Milk | 0.671 | 1.746 | 0.625 | 1.551 |
| MCFA | MSC | 4000~400 | RidgeR | Fat | 0.095 | 1.049 | 0.178 | 1.093 |
| MCFA | DER1 | 4000~400 | RidgeR | Fat | 0.525 | 1.307 | 0.219 | 1.135 |
| MCFA | DER2 | 4000~400 | RidgeR | Fat | 0.555 | 1.314 | 0.225 | 1.136 |
| MCFA | SNV | 4000~400 | RidgeR | Fat | 0.096 | 1.049 | 0.175 | 1.089 |
| MCFA | SG | 4000~400 | RidgeR | Fat | 0.073 | 1.035 | 0.146 | 1.067 |
| MCFA | MSC | 4000~400 | RFR | Fat | 0.105 | 1.058 | 0.127 | 1.070 |
| MCFA | DER1 | 4000~400 | RFR | Fat | 0.105 | 1.058 | 0.119 | 1.057 |

| | | | | | | | | |
|---|---|---|---|---|---|---|---|---|
| MCFA | DER2 | 4000~400 | RFR | Fat | 0.114 | 1.063 | 0.158 | 1.088 |
| MCFA | SNV | 4000~400 | RFR | Fat | 0.118 | 1.065 | 0.075 | 1.035 |
| MCFA | SG | 4000~400 | RFR | Fat | 0.110 | 1.059 | 0.120 | 1.067 |
| MCFA | MSC | 4000~400 | PLSR | Fat | 0.030 | 1.018 | 0.154 | 1.094 |
| MCFA | DER1 | 4000~400 | PLSR | Fat | 0.061 | 1.085 | 0.069 | 1.043 |
| MCFA | DER2 | 4000~400 | PLSR | Fat | 0.042 | 1.024 | 0.134 | 1.081 |
| MCFA | SNV | 4000~400 | PLSR | Fat | 0.027 | 1.017 | 0.147 | 1.090 |
| MCFA | SG | 4000~400 | PLSR | Fat | 0.026 | 1.024 | 0.271 | 1.179 |
| MCFA | MSC | 4000~400 | LassoR | Fat | 0.714 | 1.742 | 0.197 | 1.053 |
| MCFA | DER1 | 4000~400 | LassoR | Fat | 0.917 | 3.072 | 0.124 | 0.935 |
| MCFA | DER2 | 4000~400 | LassoR | Fat | 0.993 | 9.652 | 0.205 | 0.958 |
| MCFA | SNV | 4000~400 | LassoR | Fat | 0.713 | 1.744 | 0.184 | 1.039 |
| MCFA | SG | 4000~400 | LassoR | Fat | 0.606 | 1.523 | 0.249 | 1.134 |
| MCFA | MSC | 4000~400 | LassoR | Milk | 0.890 | 2.982 | 0.604 | 1.448 |
| MCFA | DER1 | 4000~400 | LassoR | Milk | 0.984 | 7.722 | 0.608 | 1.310 |
| MCFA | DER2 | 4000~400 | LassoR | Milk | 0.998 | 18.937 | 0.605 | 1.434 |
| MCFA | SNV | 4000~400 | LassoR | Milk | 0.937 | 3.943 | 0.563 | 1.276 |
| MCFA | SG | 4000~400 | LassoR | Milk | 0.898 | 3.117 | 0.571 | 1.328 |
| MCFA | MSC | 4000~400 | PLSR | Milk | 0.654 | 1.704 | 0.632 | 1.659 |
| MCFA | DER1 | 4000~400 | PLSR | Milk | 0.665 | 1.736 | 0.610 | 1.612 |
| MCFA | DER2 | 4000~400 | PLSR | Milk | 0.673 | 1.765 | 0.612 | 1.615 |
| MCFA | SNV | 4000~400 | PLSR | Milk | 0.657 | 1.713 | 0.647 | 1.695 |
| MCFA | SG | 4000~400 | PLSR | Milk | 0.657 | 1.713 | 0.566 | 1.528 |
| MCFA | MSC | 4000~400 | RFR | Milk | 0.655 | 1.705 | 0.737 | 1.947 |
| MCFA | DER1 | 4000~400 | RFR | Milk | 0.693 | 1.808 | 0.732 | 1.929 |
| MCFA | DER2 | 4000~400 | RFR | Milk | 0.671 | 1.746 | 0.746 | 1.980 |
| MCFA | SNV | 4000~400 | RFR | Milk | 0.657 | 1.711 | 0.707 | 1.842 |
| MCFA | SG | 4000~400 | RFR | Milk | 0.667 | 1.736 | 0.748 | 1.979 |
| MCFA | MSC | 4000~400 | RidgeR | Milk | 0.697 | 1.814 | 0.663 | 1.700 |
| MCFA | DER1 | 4000~400 | RidgeR | Milk | 0.748 | 1.981 | 0.670 | 1.685 |
| MCFA | DER2 | 4000~400 | RidgeR | Milk | 0.792 | 2.153 | 0.673 | 1.700 |
| MCFA | SNV | 4000~400 | RidgeR | Milk | 0.701 | 1.824 | 0.679 | 1.751 |
| MCFA | SG | 4000~400 | RidgeR | Milk | 0.704 | 1.837 | 0.643 | 1.615 |
| MUFA | MSC | 3017~2823/1805~1734 | LassoR | Fat | 0.530 | 1.460 | 0.412 | 1.280 |
| MUFA | DER1 | 3017~2823/1805~1734 | LassoR | Fat | 0.561 | 1.508 | 0.425 | 1.300 |
| MUFA | DER2 | 3017~2823/1805~1734 | LassoR | Fat | 0.546 | 1.484 | 0.389 | 1.253 |
| MUFA | SNV | 3017~2823/1805~1734 | LassoR | Fat | 0.521 | 1.446 | 0.411 | 1.281 |
| MUFA | SG | 3017~2823/1805~1734 | LassoR | Fat | 0.450 | 1.350 | 0.397 | 1.281 |
| MUFA | MSC | 3017~2823/1805~1734 | RidgeR | Fat | 0.356 | 1.242 | 0.368 | 1.260 |
| MUFA | DER1 | 3017~2823/1805~1734 | RidgeR | Fat | 0.420 | 1.310 | 0.379 | 1.261 |
| MUFA | DER2 | 3017~2823/1805~1734 | RidgeR | Fat | 0.471 | 1.373 | 0.383 | 1.260 |
| MUFA | SNV | 3017~2823/1805~1734 | RidgeR | Fat | 0.346 | 1.234 | 0.374 | 1.266 |
| MUFA | SG | 3017~2823/1805~1734 | RidgeR | Fat | 0.288 | 1.185 | 0.298 | 1.186 |
| MUFA | MSC | 3017~2823/1805~1734 | RFR | Fat | 0.156 | 1.078 | 0.300 | 1.177 |
| MUFA | DER1 | 3017~2823/1805~1734 | RFR | Fat | 0.144 | 1.074 | 0.350 | 1.244 |
| MUFA | DER2 | 3017~2823/1805~1734 | RFR | Fat | 0.209 | 1.126 | 0.309 | 1.204 |
| MUFA | SNV | 3017~2823/1805~1734 | RFR | Fat | 0.126 | 1.050 | 0.272 | 1.136 |
| MUFA | SG | 3017~2823/1805~1734 | RFR | Fat | 0.140 | 1.063 | 0.194 | 1.094 |
| MUFA | MSC | 3017~2823/1805~1734 | PLSR | Fat | 0.409 | 1.314 | 0.285 | 1.190 |
| MUFA | DER1 | 3017~2823/1805~1734 | PLSR | Fat | 0.374 | 1.279 | 0.322 | 1.222 |

| | | | | | | | | |
|---|---|---|---|---|---|---|---|---|
| MUFA | DER2 | 3017~2823/1805~1734 | PLSR | Fat | 0.351 | 1.254 | 0.299 | 1.202 |
| MUFA | SNV | 3017~2823/1805~1734 | PLSR | Fat | 0.421 | 1.326 | 0.274 | 1.181 |
| MUFA | SG | 3017~2823/1805~1734 | PLSR | Fat | 0.386 | 1.284 | 0.377 | 1.275 |
| MUFA | MSC | 3017~2823/1805~1734 | LassoR | Milk | 0.602 | 1.588 | 0.748 | 1.899 |
| MUFA | DER1 | 3017~2823/1805~1734 | LassoR | Milk | 0.593 | 1.571 | 0.786 | 2.033 |
| MUFA | DER2 | 3017~2823/1805~1734 | LassoR | Milk | 0.605 | 1.593 | 0.793 | 2.064 |
| MUFA | SNV | 3017~2823/1805~1734 | LassoR | Milk | 0.637 | 1.663 | 0.753 | 1.930 |
| MUFA | SG | 3017~2823/1805~1734 | LassoR | Milk | 0.570 | 1.527 | 0.778 | 1.973 |
| MUFA | MSC | 3017~2823/1805~1734 | PLSR | Milk | 0.547 | 1.491 | 0.695 | 1.822 |
| MUFA | DER1 | 3017~2823/1805~1734 | PLSR | Milk | 0.513 | 1.439 | 0.757 | 2.039 |
| MUFA | DER2 | 3017~2823/1805~1734 | PLSR | Milk | 0.517 | 1.445 | 0.743 | 1.984 |
| MUFA | SNV | 3017~2823/1805~1734 | PLSR | Milk | 0.535 | 1.471 | 0.708 | 1.863 |
| MUFA | SG | 3017~2823/1805~1734 | PLSR | Milk | 0.509 | 1.435 | 0.750 | 2.014 |
| MUFA | MSC | 3017~2823/1805~1734 | RFR | Milk | 0.437 | 1.328 | 0.623 | 1.615 |
| MUFA | DER1 | 3017~2823/1805~1734 | RFR | Milk | 0.460 | 1.359 | 0.726 | 1.815 |
| MUFA | DER2 | 3017~2823/1805~1734 | RFR | Milk | 0.498 | 1.414 | 0.683 | 1.704 |
| MUFA | SNV | 3017~2823/1805~1734 | RFR | Milk | 0.439 | 1.327 | 0.648 | 1.654 |
| MUFA | SG | 3017~2823/1805~1734 | RFR | Milk | 0.435 | 1.328 | 0.638 | 1.618 |
| MUFA | MSC | 3017~2823/1805~1734 | RidgeR | Milk | 0.539 | 1.475 | 0.678 | 1.689 |
| MUFA | DER1 | 3017~2823/1805~1734 | RidgeR | Milk | 0.555 | 1.501 | 0.774 | 1.925 |
| MUFA | DER2 | 3017~2823/1805~1734 | RidgeR | Milk | 0.569 | 1.525 | 0.781 | 1.948 |
| MUFA | SNV | 3017~2823/1805~1734 | RidgeR | Milk | 0.532 | 1.465 | 0.680 | 1.695 |
| MUFA | SG | 3017~2823/1805~1734 | RidgeR | Milk | 0.513 | 1.435 | 0.708 | 1.738 |
| MUFA | MSC | 4000~400 | RidgeR | Fat | 0.409 | 1.272 | 0.345 | 1.243 |
| MUFA | DER1 | 4000~400 | RidgeR | Fat | 0.541 | 1.420 | 0.423 | 1.324 |
| MUFA | DER2 | 4000~400 | RidgeR | Fat | 0.672 | 1.612 | 0.409 | 1.306 |
| MUFA | SNV | 4000~400 | RidgeR | Fat | 0.410 | 1.280 | 0.373 | 1.270 |
| MUFA | SG | 4000~400 | RidgeR | Fat | 0.367 | 1.242 | 0.344 | 1.241 |
| MUFA | MSC | 4000~400 | RFR | Fat | 0.205 | 1.123 | 0.245 | 1.153 |
| MUFA | DER1 | 4000~400 | RFR | Fat | 0.231 | 1.142 | 0.350 | 1.246 |
| MUFA | DER2 | 4000~400 | RFR | Fat | 0.227 | 1.139 | 0.404 | 1.302 |
| MUFA | SNV | 4000~400 | RFR | Fat | 0.220 | 1.134 | 0.233 | 1.132 |
| MUFA | SG | 4000~400 | RFR | Fat | 0.175 | 1.098 | 0.220 | 1.129 |
| MUFA | MSC | 4000~400 | PLSR | Fat | 0.237 | 1.156 | 0.293 | 1.197 |
| MUFA | DER1 | 4000~400 | PLSR | Fat | 0.224 | 1.145 | 0.363 | 1.261 |
| MUFA | DER2 | 4000~400 | PLSR | Fat | 0.206 | 1.133 | 0.281 | 1.187 |
| MUFA | SNV | 4000~400 | PLSR | Fat | 0.202 | 1.133 | 0.293 | 1.197 |
| MUFA | SG | 4000~400 | PLSR | Fat | 0.208 | 1.135 | 0.282 | 1.187 |
| MUFA | MSC | 4000~400 | LassoR | Fat | 0.799 | 2.180 | 0.245 | 1.062 |
| MUFA | DER1 | 4000~400 | LassoR | Fat | 0.941 | 3.828 | 0.274 | 1.055 |
| MUFA | DER2 | 4000~400 | LassoR | Fat | 0.994 | 11.082 | 0.251 | 0.925 |
| MUFA | SNV | 4000~400 | LassoR | Fat | 0.817 | 2.282 | 0.234 | 1.037 |
| MUFA | SG | 4000~400 | LassoR | Fat | 0.747 | 1.952 | 0.300 | 1.103 |
| MUFA | MSC | 4000~400 | LassoR | Milk | 0.872 | 2.752 | 0.488 | 1.397 |
| MUFA | DER1 | 4000~400 | LassoR | Milk | 0.975 | 6.028 | 0.505 | 1.373 |
| MUFA | DER2 | 4000~400 | LassoR | Milk | 0.995 | 12.861 | 0.505 | 1.405 |
| MUFA | SNV | 4000~400 | LassoR | Milk | 0.875 | 2.778 | 0.501 | 1.416 |
| MUFA | SG | 4000~400 | LassoR | Milk | 0.810 | 2.272 | 0.550 | 1.500 |
| MUFA | MSC | 4000~400 | PLSR | Milk | 0.488 | 1.403 | 0.637 | 1.670 |
| MUFA | DER1 | 4000~400 | PLSR | Milk | 0.463 | 1.398 | 0.614 | 1.621 |

| | | | | | | | | |
|---|---|---|---|---|---|---|---|---|
| MUFA | DER2 | 4000~400 | PLSR | Milk | 0.442 | 1.383 | 0.632 | 1.658 |
| MUFA | SNV | 4000~400 | PLSR | Milk | 0.490 | 1.405 | 0.637 | 1.671 |
| MUFA | SG | 4000~400 | PLSR | Milk | 0.474 | 1.383 | 0.620 | 1.633 |
| MUFA | MSC | 4000~400 | RFR | Milk | 0.469 | 1.373 | 0.628 | 1.594 |
| MUFA | DER1 | 4000~400 | RFR | Milk | 0.474 | 1.381 | 0.720 | 1.747 |
| MUFA | DER2 | 4000~400 | RFR | Milk | 0.483 | 1.393 | 0.722 | 1.762 |
| MUFA | SNV | 4000~400 | RFR | Milk | 0.475 | 1.378 | 0.651 | 1.634 |
| MUFA | SG | 4000~400 | RFR | Milk | 0.458 | 1.360 | 0.636 | 1.597 |
| MUFA | MSC | 4000~400 | RidgeR | Milk | 0.576 | 1.534 | 0.653 | 1.627 |
| MUFA | DER1 | 4000~400 | RidgeR | Milk | 0.639 | 1.652 | 0.730 | 1.794 |
| MUFA | DER2 | 4000~400 | RidgeR | Milk | 0.709 | 1.794 | 0.726 | 1.744 |
| MUFA | SNV | 4000~400 | RidgeR | Milk | 0.577 | 1.536 | 0.654 | 1.629 |
| MUFA | SG | 4000~400 | RidgeR | Milk | 0.557 | 1.503 | 0.684 | 1.672 |
| PUFA | MSC | 3017~2823/1805~1734 | LassoR | Fat | 0.234 | 1.145 | 0.111 | 1.056 |
| PUFA | DER1 | 3017~2823/1805~1734 | LassoR | Fat | 0.263 | 1.167 | 0.096 | 1.040 |
| PUFA | DER2 | 3017~2823/1805~1734 | LassoR | Fat | 0.297 | 1.194 | 0.070 | 1.018 |
| PUFA | SNV | 3017~2823/1805~1734 | LassoR | Fat | 0.227 | 1.139 | 0.104 | 1.051 |
| PUFA | SG | 3017~2823/1805~1734 | LassoR | Fat | 0.224 | 1.138 | 0.092 | 1.037 |
| PUFA | MSC | 3017~2823/1805~1734 | RidgeR | Fat | 0.210 | 1.127 | 0.081 | 1.036 |
| PUFA | DER1 | 3017~2823/1805~1734 | RidgeR | Fat | 0.238 | 1.148 | 0.092 | 1.040 |
| PUFA | DER2 | 3017~2823/1805~1734 | RidgeR | Fat | 0.241 | 1.149 | 0.083 | 1.036 |
| PUFA | SNV | 3017~2823/1805~1734 | RidgeR | Fat | 0.210 | 1.126 | 0.084 | 1.039 |
| PUFA | SG | 3017~2823/1805~1734 | RidgeR | Fat | 0.215 | 1.130 | 0.101 | 1.050 |
| PUFA | MSC | 3017~2823/1805~1734 | RFR | Fat | 0.162 | 1.075 | 0.144 | 1.058 |
| PUFA | DER1 | 3017~2823/1805~1734 | RFR | Fat | 0.162 | 1.076 | 0.143 | 1.066 |
| PUFA | DER2 | 3017~2823/1805~1734 | RFR | Fat | 0.211 | 1.120 | 0.116 | 1.038 |
| PUFA | SNV | 3017~2823/1805~1734 | RFR | Fat | 0.145 | 1.065 | 0.154 | 1.074 |
| PUFA | SG | 3017~2823/1805~1734 | RFR | Fat | 0.161 | 1.076 | 0.162 | 1.074 |
| PUFA | MSC | 3017~2823/1805~1734 | PLSR | Fat | 0.195 | 1.118 | 0.043 | 1.029 |
| PUFA | DER1 | 3017~2823/1805~1734 | PLSR | Fat | 0.193 | 1.116 | 0.028 | 1.021 |
| PUFA | DER2 | 3017~2823/1805~1734 | PLSR | Fat | 0.186 | 1.111 | 0.059 | 1.037 |
| PUFA | SNV | 3017~2823/1805~1734 | PLSR | Fat | 0.192 | 1.116 | 0.043 | 1.029 |
| PUFA | SG | 3017~2823/1805~1734 | PLSR | Fat | 0.197 | 1.119 | 0.075 | 1.046 |
| PUFA | MSC | 3017~2823/1805~1734 | LassoR | Milk | 0.653 | 1.702 | 0.672 | 1.756 |
| PUFA | DER1 | 3017~2823/1805~1734 | LassoR | Milk | 0.656 | 1.708 | 0.659 | 1.718 |
| PUFA | DER2 | 3017~2823/1805~1734 | LassoR | Milk | 0.659 | 1.715 | 0.652 | 1.705 |
| PUFA | SNV | 3017~2823/1805~1734 | LassoR | Milk | 0.657 | 1.711 | 0.670 | 1.749 |
| PUFA | SG | 3017~2823/1805~1734 | LassoR | Milk | 0.589 | 1.563 | 0.681 | 1.748 |
| PUFA | MSC | 3017~2823/1805~1734 | PLSR | Milk | 0.587 | 1.564 | 0.672 | 1.757 |
| PUFA | DER1 | 3017~2823/1805~1734 | PLSR | Milk | 0.570 | 1.536 | 0.630 | 1.653 |
| PUFA | DER2 | 3017~2823/1805~1734 | PLSR | Milk | 0.549 | 1.505 | 0.640 | 1.678 |
| PUFA | SNV | 3017~2823/1805~1734 | PLSR | Milk | 0.586 | 1.564 | 0.668 | 1.747 |
| PUFA | SG | 3017~2823/1805~1734 | PLSR | Milk | 0.567 | 1.532 | 0.637 | 1.671 |
| PUFA | MSC | 3017~2823/1805~1734 | RFR | Milk | 0.589 | 1.555 | 0.645 | 1.685 |
| PUFA | DER1 | 3017~2823/1805~1734 | RFR | Milk | 0.564 | 1.516 | 0.672 | 1.749 |
| PUFA | DER2 | 3017~2823/1805~1734 | RFR | Milk | 0.552 | 1.497 | 0.658 | 1.713 |
| PUFA | SNV | 3017~2823/1805~1734 | RFR | Milk | 0.564 | 1.513 | 0.627 | 1.647 |
| PUFA | SG | 3017~2823/1805~1734 | RFR | Milk | 0.588 | 1.559 | 0.631 | 1.654 |
| PUFA | MSC | 3017~2823/1805~1734 | RidgeR | Milk | 0.613 | 1.611 | 0.689 | 1.788 |
| PUFA | DER1 | 3017~2823/1805~1734 | RidgeR | Milk | 0.597 | 1.577 | 0.686 | 1.752 |

| | | | | | | | | |
|---|---|---|---|---|---|---|---|---|
| PUFA | DER2 | 3017~2823/1805~1734 | RidgeR | Milk | 0.600 | 1.583 | 0.676 | 1.728 |
| PUFA | SNV | 3017~2823/1805~1734 | RidgeR | Milk | 0.613 | 1.610 | 0.692 | 1.792 |
| PUFA | SG | 3017~2823/1805~1734 | RidgeR | Milk | 0.578 | 1.542 | 0.687 | 1.743 |
| PUFA | MSC | 4000~400 | RidgeR | Fat | 0.247 | 1.149 | 0.084 | 1.042 |
| PUFA | DER1 | 4000~400 | RidgeR | Fat | 0.257 | 1.154 | 0.100 | 1.054 |
| PUFA | DER2 | 4000~400 | RidgeR | Fat | 0.318 | 1.194 | 0.103 | 1.056 |
| PUFA | SNV | 4000~400 | RidgeR | Fat | 0.244 | 1.146 | 0.081 | 1.041 |
| PUFA | SG | 4000~400 | RidgeR | Fat | 0.247 | 1.151 | 0.103 | 1.052 |
| PUFA | MSC | 4000~400 | RFR | Fat | 0.168 | 1.095 | 0.077 | 1.025 |
| PUFA | DER1 | 4000~400 | RFR | Fat | 0.166 | 1.088 | 0.128 | 1.064 |
| PUFA | DER2 | 4000~400 | RFR | Fat | 0.180 | 1.104 | 0.116 | 1.052 |
| PUFA | SNV | 4000~400 | RFR | Fat | 0.152 | 1.083 | 0.117 | 1.053 |
| PUFA | SG | 4000~400 | RFR | Fat | 0.145 | 1.068 | 0.132 | 1.061 |
| PUFA | MSC | 4000~400 | PLSR | Fat | 0.171 | 1.101 | 0.026 | 1.019 |
| PUFA | DER1 | 4000~400 | PLSR | Fat | 0.196 | 1.118 | 0.053 | 1.034 |
| PUFA | DER2 | 4000~400 | PLSR | Fat | 0.198 | 1.119 | 0.074 | 1.045 |
| PUFA | SNV | 4000~400 | PLSR | Fat | 0.171 | 1.101 | 0.026 | 1.020 |
| PUFA | SG | 4000~400 | PLSR | Fat | 0.195 | 1.118 | 0.074 | 1.046 |
| PUFA | MSC | 4000~400 | LassoR | Fat | 0.669 | 1.687 | 0.048 | 0.924 |
| PUFA | DER1 | 4000~400 | LassoR | Fat | 0.898 | 2.784 | 0.119 | 0.965 |
| PUFA | DER2 | 4000~400 | LassoR | Fat | 0.982 | 6.087 | 0.081 | 0.903 |
| PUFA | SNV | 4000~400 | LassoR | Fat | 0.670 | 1.689 | 0.054 | 0.928 |
| PUFA | SG | 4000~400 | LassoR | Fat | 0.579 | 1.517 | 0.033 | 0.913 |
| PUFA | MSC | 4000~400 | LassoR | Milk | 0.852 | 2.585 | 0.521 | 1.419 |
| PUFA | DER1 | 4000~400 | LassoR | Milk | 0.961 | 4.847 | 0.517 | 1.386 |
| PUFA | DER2 | 4000~400 | LassoR | Milk | 0.989 | 8.826 | 0.572 | 1.520 |
| PUFA | SNV | 4000~400 | LassoR | Milk | 0.853 | 2.588 | 0.530 | 1.435 |
| PUFA | SG | 4000~400 | LassoR | Milk | 0.836 | 2.460 | 0.514 | 1.410 |
| PUFA | MSC | 4000~400 | PLSR | Milk | 0.594 | 1.575 | 0.673 | 1.759 |
| PUFA | DER1 | 4000~400 | PLSR | Milk | 0.551 | 1.499 | 0.649 | 1.697 |
| PUFA | DER2 | 4000~400 | PLSR | Milk | 0.513 | 1.439 | 0.672 | 1.757 |
| PUFA | SNV | 4000~400 | PLSR | Milk | 0.595 | 1.576 | 0.670 | 1.751 |
| PUFA | SG | 4000~400 | PLSR | Milk | 0.563 | 1.520 | 0.659 | 1.724 |
| PUFA | MSC | 4000~400 | RFR | Milk | 0.584 | 1.553 | 0.663 | 1.719 |
| PUFA | DER1 | 4000~400 | RFR | Milk | 0.581 | 1.547 | 0.657 | 1.703 |
| PUFA | DER2 | 4000~400 | RFR | Milk | 0.569 | 1.526 | 0.684 | 1.755 |
| PUFA | SNV | 4000~400 | RFR | Milk | 0.587 | 1.559 | 0.651 | 1.692 |
| PUFA | SG | 4000~400 | RFR | Milk | 0.589 | 1.564 | 0.702 | 1.818 |
| PUFA | MSC | 4000~400 | RidgeR | Milk | 0.635 | 1.651 | 0.676 | 1.733 |
| PUFA | DER1 | 4000~400 | RidgeR | Milk | 0.643 | 1.668 | 0.685 | 1.752 |
| PUFA | DER2 | 4000~400 | RidgeR | Milk | 0.705 | 1.800 | 0.705 | 1.754 |
| PUFA | SNV | 4000~400 | RidgeR | Milk | 0.635 | 1.649 | 0.679 | 1.735 |
| PUFA | SG | 4000~400 | RidgeR | Milk | 0.629 | 1.641 | 0.681 | 1.748 |
| SCFA | MSC | 3017~2823/1805~1734 | LassoR | Fat | 0.507 | 1.426 | 0.475 | 1.366 |
| SCFA | DER1 | 3017~2823/1805~1734 | LassoR | Fat | 0.552 | 1.493 | 0.465 | 1.366 |
| SCFA | DER2 | 3017~2823/1805~1734 | LassoR | Fat | 0.516 | 1.436 | 0.416 | 1.304 |
| SCFA | SNV | 3017~2823/1805~1734 | LassoR | Fat | 0.506 | 1.424 | 0.473 | 1.365 |
| SCFA | SG | 3017~2823/1805~1734 | LassoR | Fat | 0.487 | 1.394 | 0.463 | 1.344 |
| SCFA | MSC | 3017~2823/1805~1734 | RidgeR | Fat | 0.340 | 1.227 | 0.372 | 1.235 |
| SCFA | DER1 | 3017~2823/1805~1734 | RidgeR | Fat | 0.384 | 1.269 | 0.376 | 1.259 |

| | | | | | | | | |
|---|---|---|---|---|---|---|---|---|
| SCFA | DER2 | 3017~2823/1805~1734 | RidgeR | Fat | 0.427 | 1.316 | 0.422 | 1.296 |
| SCFA | SNV | 3017~2823/1805~1734 | RidgeR | Fat | 0.344 | 1.228 | 0.346 | 1.213 |
| SCFA | SG | 3017~2823/1805~1734 | RidgeR | Fat | 0.262 | 1.158 | 0.236 | 1.137 |
| SCFA | MSC | 3017~2823/1805~1734 | RFR | Fat | 0.159 | 1.084 | 0.151 | 1.057 |
| SCFA | DER1 | 3017~2823/1805~1734 | RFR | Fat | 0.096 | 1.043 | 0.228 | 1.136 |
| SCFA | DER2 | 3017~2823/1805~1734 | RFR | Fat | 0.135 | 1.077 | 0.282 | 1.170 |
| SCFA | SNV | 3017~2823/1805~1734 | RFR | Fat | 0.151 | 1.077 | 0.151 | 1.063 |
| SCFA | SG | 3017~2823/1805~1734 | RFR | Fat | 0.076 | 1.018 | 0.079 | 1.011 |
| SCFA | MSC | 3017~2823/1805~1734 | PLSR | Fat | 0.421 | 1.324 | 0.459 | 1.368 |
| SCFA | DER1 | 3017~2823/1805~1734 | PLSR | Fat | 0.374 | 1.279 | 0.383 | 1.281 |
| SCFA | DER2 | 3017~2823/1805~1734 | PLSR | Fat | 0.336 | 1.241 | 0.338 | 1.237 |
| SCFA | SNV | 3017~2823/1805~1734 | PLSR | Fat | 0.422 | 1.325 | 0.454 | 1.362 |
| SCFA | SG | 3017~2823/1805~1734 | PLSR | Fat | 0.425 | 1.326 | 0.445 | 1.350 |
| SCFA | MSC | 3017~2823/1805~1734 | LassoR | Milk | 0.758 | 2.036 | 0.744 | 1.944 |
| SCFA | DER1 | 3017~2823/1805~1734 | LassoR | Milk | 0.789 | 2.180 | 0.701 | 1.714 |
| SCFA | DER2 | 3017~2823/1805~1734 | LassoR | Milk | 0.778 | 2.128 | 0.701 | 1.752 |
| SCFA | SNV | 3017~2823/1805~1734 | LassoR | Milk | 0.762 | 2.056 | 0.756 | 1.996 |
| SCFA | SG | 3017~2823/1805~1734 | LassoR | Milk | 0.756 | 2.027 | 0.669 | 1.657 |
| SCFA | MSC | 3017~2823/1805~1734 | PLSR | Milk | 0.722 | 1.911 | 0.686 | 1.797 |
| SCFA | DER1 | 3017~2823/1805~1734 | PLSR | Milk | 0.711 | 1.873 | 0.638 | 1.674 |
| SCFA | DER2 | 3017~2823/1805~1734 | PLSR | Milk | 0.697 | 1.834 | 0.664 | 1.736 |
| SCFA | SNV | 3017~2823/1805~1734 | PLSR | Milk | 0.722 | 1.914 | 0.709 | 1.866 |
| SCFA | SG | 3017~2823/1805~1734 | PLSR | Milk | 0.722 | 1.907 | 0.620 | 1.633 |
| SCFA | MSC | 3017~2823/1805~1734 | RFR | Milk | 0.607 | 1.589 | 0.719 | 1.883 |
| SCFA | DER1 | 3017~2823/1805~1734 | RFR | Milk | 0.640 | 1.670 | 0.768 | 2.060 |
| SCFA | DER2 | 3017~2823/1805~1734 | RFR | Milk | 0.624 | 1.633 | 0.769 | 1.990 |
| SCFA | SNV | 3017~2823/1805~1734 | RFR | Milk | 0.606 | 1.590 | 0.731 | 1.921 |
| SCFA | SG | 3017~2823/1805~1734 | RFR | Milk | 0.586 | 1.555 | 0.741 | 1.898 |
| SCFA | MSC | 3017~2823/1805~1734 | RidgeR | Milk | 0.702 | 1.832 | 0.733 | 1.935 |
| SCFA | DER1 | 3017~2823/1805~1734 | RidgeR | Milk | 0.726 | 1.911 | 0.647 | 1.619 |
| SCFA | DER2 | 3017~2823/1805~1734 | RidgeR | Milk | 0.744 | 1.977 | 0.662 | 1.646 |
| SCFA | SNV | 3017~2823/1805~1734 | RidgeR | Milk | 0.697 | 1.818 | 0.733 | 1.933 |
| SCFA | SG | 3017~2823/1805~1734 | RidgeR | Milk | 0.657 | 1.709 | 0.611 | 1.560 |
| SCFA | MSC | 4000~400 | RidgeR | Fat | 0.434 | 1.259 | 0.257 | 1.139 |
| SCFA | DER1 | 4000~400 | RidgeR | Fat | 0.551 | 1.406 | 0.274 | 1.148 |
| SCFA | DER2 | 4000~400 | RidgeR | Fat | 0.664 | 1.521 | 0.321 | 1.181 |
| SCFA | SNV | 4000~400 | RidgeR | Fat | 0.423 | 1.253 | 0.270 | 1.144 |
| SCFA | SG | 4000~400 | RidgeR | Fat | 0.373 | 1.218 | 0.236 | 1.121 |
| SCFA | MSC | 4000~400 | RFR | Fat | 0.110 | 1.059 | 0.234 | 1.140 |
| SCFA | DER1 | 4000~400 | RFR | Fat | 0.182 | 1.108 | 0.264 | 1.144 |
| SCFA | DER2 | 4000~400 | RFR | Fat | 0.175 | 1.103 | 0.267 | 1.140 |
| SCFA | SNV | 4000~400 | RFR | Fat | 0.104 | 1.056 | 0.223 | 1.127 |
| SCFA | SG | 4000~400 | RFR | Fat | 0.081 | 1.038 | 0.203 | 1.115 |
| SCFA | MSC | 4000~400 | PLSR | Fat | 0.235 | 1.158 | 0.275 | 1.182 |
| SCFA | DER1 | 4000~400 | PLSR | Fat | 0.234 | 1.148 | 0.237 | 1.152 |
| SCFA | DER2 | 4000~400 | PLSR | Fat | 0.185 | 1.121 | 0.254 | 1.165 |
| SCFA | SNV | 4000~400 | PLSR | Fat | 0.212 | 1.143 | 0.233 | 1.149 |
| SCFA | SG | 4000~400 | PLSR | Fat | 0.136 | 1.095 | 0.118 | 1.072 |
| SCFA | MSC | 4000~400 | LassoR | Fat | 0.843 | 2.472 | 0.267 | 1.096 |
| SCFA | DER1 | 4000~400 | LassoR | Fat | 0.947 | 4.041 | 0.264 | 1.061 |

| | | | | | | | | |
|---|---|---|---|---|---|---|---|---|
| SCFA | DER2 | 4000~400 | LassoR | Fat | 0.991 | 8.974 | 0.336 | 1.175 |
| SCFA | SNV | 4000~400 | LassoR | Fat | 0.847 | 2.510 | 0.269 | 1.095 |
| SCFA | SG | 4000~400 | LassoR | Fat | 0.751 | 1.971 | 0.238 | 1.104 |
| SCFA | MSC | 4000~400 | LassoR | Milk | 0.931 | 3.791 | 0.685 | 1.733 |
| SCFA | DER1 | 4000~400 | LassoR | Milk | 0.974 | 6.004 | 0.695 | 1.705 |
| SCFA | DER2 | 4000~400 | LassoR | Milk | 0.999 | 30.665 | 0.695 | 1.692 |
| SCFA | SNV | 4000~400 | LassoR | Milk | 0.927 | 3.671 | 0.711 | 1.826 |
| SCFA | SG | 4000~400 | LassoR | Milk | 0.879 | 2.861 | 0.689 | 1.761 |
| SCFA | MSC | 4000~400 | PLSR | Milk | 0.667 | 1.761 | 0.727 | 1.927 |
| SCFA | DER1 | 4000~400 | PLSR | Milk | 0.644 | 1.685 | 0.714 | 1.881 |
| SCFA | DER2 | 4000~400 | PLSR | Milk | 0.657 | 1.780 | 0.676 | 1.769 |
| SCFA | SNV | 4000~400 | PLSR | Milk | 0.669 | 1.777 | 0.729 | 1.931 |
| SCFA | SG | 4000~400 | PLSR | Milk | 0.630 | 1.663 | 0.686 | 1.796 |
| SCFA | MSC | 4000~400 | RFR | Milk | 0.612 | 1.608 | 0.692 | 1.768 |
| SCFA | DER1 | 4000~400 | RFR | Milk | 0.664 | 1.729 | 0.771 | 2.034 |
| SCFA | DER2 | 4000~400 | RFR | Milk | 0.655 | 1.705 | 0.773 | 2.040 |
| SCFA | SNV | 4000~400 | RFR | Milk | 0.623 | 1.632 | 0.706 | 1.825 |
| SCFA | SG | 4000~400 | RFR | Milk | 0.626 | 1.638 | 0.771 | 2.017 |
| SCFA | MSC | 4000~400 | RidgeR | Milk | 0.684 | 1.766 | 0.676 | 1.725 |
| SCFA | DER1 | 4000~400 | RidgeR | Milk | 0.730 | 1.911 | 0.691 | 1.765 |
| SCFA | DER2 | 4000~400 | RidgeR | Milk | 0.781 | 2.083 | 0.694 | 1.767 |
| SCFA | SNV | 4000~400 | RidgeR | Milk | 0.698 | 1.803 | 0.701 | 1.793 |
| SCFA | SG | 4000~400 | RidgeR | Milk | 0.674 | 1.747 | 0.631 | 1.605 |
| SFA | MSC | 3017~2823/1805~1734 | LassoR | Fat | 0.295 | 1.191 | 0.163 | 1.084 |
| SFA | DER1 | 3017~2823/1805~1734 | LassoR | Fat | 0.372 | 1.262 | 0.224 | 1.123 |
| SFA | DER2 | 3017~2823/1805~1734 | LassoR | Fat | 0.385 | 1.275 | 0.184 | 1.090 |
| SFA | SNV | 3017~2823/1805~1734 | LassoR | Fat | 0.292 | 1.188 | 0.156 | 1.080 |
| SFA | SG | 3017~2823/1805~1734 | LassoR | Fat | 0.320 | 1.212 | 0.254 | 1.155 |
| SFA | MSC | 3017~2823/1805~1734 | RidgeR | Fat | 0.205 | 1.119 | 0.102 | 1.052 |
| SFA | DER1 | 3017~2823/1805~1734 | RidgeR | Fat | 0.268 | 1.161 | 0.212 | 1.127 |
| SFA | DER2 | 3017~2823/1805~1734 | RidgeR | Fat | 0.300 | 1.192 | 0.193 | 1.115 |
| SFA | SNV | 3017~2823/1805~1734 | RidgeR | Fat | 0.186 | 1.107 | 0.076 | 1.036 |
| SFA | SG | 3017~2823/1805~1734 | RidgeR | Fat | 0.153 | 1.088 | 0.071 | 1.036 |
| SFA | MSC | 3017~2823/1805~1734 | RFR | Fat | 0.074 | 1.019 | 0.057 | 1.002 |
| SFA | DER1 | 3017~2823/1805~1734 | RFR | Fat | 0.028 | 0.995 | 0.127 | 1.061 |
| SFA | DER2 | 3017~2823/1805~1734 | RFR | Fat | 0.072 | 1.025 | 0.090 | 1.043 |
| SFA | SNV | 3017~2823/1805~1734 | RFR | Fat | 0.058 | 1.005 | 0.054 | 0.991 |
| SFA | SG | 3017~2823/1805~1734 | RFR | Fat | 0.026 | 0.984 | 0.108 | 1.047 |
| SFA | MSC | 3017~2823/1805~1734 | PLSR | Fat | 0.194 | 1.123 | 0.087 | 1.053 |
| SFA | DER1 | 3017~2823/1805~1734 | PLSR | Fat | 0.205 | 1.128 | 0.136 | 1.082 |
| SFA | DER2 | 3017~2823/1805~1734 | PLSR | Fat | 0.159 | 1.099 | 0.113 | 1.069 |
| SFA | SNV | 3017~2823/1805~1734 | PLSR | Fat | 0.201 | 1.137 | 0.166 | 1.102 |
| SFA | SG | 3017~2823/1805~1734 | PLSR | Fat | 0.218 | 1.138 | 0.225 | 1.143 |
| SFA | MSC | 3017~2823/1805~1734 | LassoR | Milk | 0.731 | 1.931 | 0.689 | 1.770 |
| SFA | DER1 | 3017~2823/1805~1734 | LassoR | Milk | 0.767 | 2.074 | 0.639 | 1.591 |
| SFA | DER2 | 3017~2823/1805~1734 | LassoR | Milk | 0.768 | 2.079 | 0.660 | 1.667 |
| SFA | SNV | 3017~2823/1805~1734 | LassoR | Milk | 0.745 | 1.984 | 0.660 | 1.687 |
| SFA | SG | 3017~2823/1805~1734 | LassoR | Milk | 0.741 | 1.970 | 0.655 | 1.629 |
| SFA | MSC | 3017~2823/1805~1734 | PLSR | Milk | 0.694 | 1.814 | 0.706 | 1.856 |
| SFA | DER1 | 3017~2823/1805~1734 | PLSR | Milk | 0.717 | 1.890 | 0.592 | 1.576 |

| | | | | | | | | |
|---|---|---|---|---|---|---|---|---|
| SFA | DER2 | 3017~2823/1805~1734 | PLSR | Milk | 0.702 | 1.839 | 0.638 | 1.672 |
| SFA | SNV | 3017~2823/1805~1734 | PLSR | Milk | 0.697 | 1.825 | 0.685 | 1.794 |
| SFA | SG | 3017~2823/1805~1734 | PLSR | Milk | 0.713 | 1.876 | 0.619 | 1.631 |
| SFA | MSC | 3017~2823/1805~1734 | RFR | Milk | 0.645 | 1.678 | 0.732 | 1.922 |
| SFA | DER1 | 3017~2823/1805~1734 | RFR | Milk | 0.662 | 1.720 | 0.750 | 2.001 |
| SFA | DER2 | 3017~2823/1805~1734 | RFR | Milk | 0.660 | 1.717 | 0.739 | 1.963 |
| SFA | SNV | 3017~2823/1805~1734 | RFR | Milk | 0.648 | 1.688 | 0.719 | 1.874 |
| SFA | SG | 3017~2823/1805~1734 | RFR | Milk | 0.664 | 1.727 | 0.758 | 2.009 |
| SFA | MSC | 3017~2823/1805~1734 | RidgeR | Milk | 0.693 | 1.809 | 0.722 | 1.874 |
| SFA | DER1 | 3017~2823/1805~1734 | RidgeR | Milk | 0.726 | 1.912 | 0.669 | 1.696 |
| SFA | DER2 | 3017~2823/1805~1734 | RidgeR | Milk | 0.739 | 1.959 | 0.665 | 1.680 |
| SFA | SNV | 3017~2823/1805~1734 | RidgeR | Milk | 0.696 | 1.818 | 0.711 | 1.842 |
| SFA | SG | 3017~2823/1805~1734 | RidgeR | Milk | 0.694 | 1.812 | 0.667 | 1.689 |
| SFA | MSC | 4000~400 | RidgeR | Fat | 0.198 | 1.109 | 0.030 | 1.009 |
| SFA | DER1 | 4000~400 | RidgeR | Fat | 0.535 | 1.368 | 0.123 | 1.065 |
| SFA | DER2 | 4000~400 | RidgeR | Fat | 0.547 | 1.361 | 0.145 | 1.084 |
| SFA | SNV | 4000~400 | RidgeR | Fat | 0.211 | 1.116 | 0.032 | 1.010 |
| SFA | SG | 4000~400 | RidgeR | Fat | 0.234 | 1.134 | 0.070 | 1.036 |
| SFA | MSC | 4000~400 | RFR | Fat | 0.148 | 1.085 | 0.054 | 1.014 |
| SFA | DER1 | 4000~400 | RFR | Fat | 0.210 | 1.126 | 0.062 | 1.018 |
| SFA | DER2 | 4000~400 | RFR | Fat | 0.196 | 1.114 | 0.128 | 1.069 |
| SFA | SNV | 4000~400 | RFR | Fat | 0.150 | 1.086 | 0.025 | 0.991 |
| SFA | SG | 4000~400 | RFR | Fat | 0.143 | 1.082 | 0.123 | 1.069 |
| SFA | MSC | 4000~400 | PLSR | Fat | 0.098 | 1.056 | -0.027 | 0.993 |
| SFA | DER1 | 4000~400 | PLSR | Fat | 0.076 | 1.063 | 0.011 | 1.012 |
| SFA | DER2 | 4000~400 | PLSR | Fat | 0.149 | 1.090 | 0.161 | 1.098 |
| SFA | SNV | 4000~400 | PLSR | Fat | 0.094 | 1.054 | -0.027 | 0.993 |
| SFA | SG | 4000~400 | PLSR | Fat | 0.126 | 1.073 | 0.028 | 1.021 |
| SFA | MSC | 4000~400 | LassoR | Fat | 0.720 | 1.798 | 0.059 | 0.925 |
| SFA | DER1 | 4000~400 | LassoR | Fat | 0.941 | 3.773 | 0.068 | 0.856 |
| SFA | DER2 | 4000~400 | LassoR | Fat | 0.988 | 7.648 | 0.096 | 0.881 |
| SFA | SNV | 4000~400 | LassoR | Fat | 0.725 | 1.818 | 0.063 | 0.926 |
| SFA | SG | 4000~400 | LassoR | Fat | 0.631 | 1.595 | 0.105 | 1.002 |
| SFA | MSC | 4000~400 | LassoR | Milk | 0.893 | 3.033 | 0.636 | 1.576 |
| SFA | DER1 | 4000~400 | LassoR | Milk | 0.977 | 6.489 | 0.667 | 1.596 |
| SFA | DER2 | 4000~400 | LassoR | Milk | 0.997 | 16.389 | 0.622 | 1.555 |
| SFA | SNV | 4000~400 | LassoR | Milk | 0.927 | 3.664 | 0.613 | 1.483 |
| SFA | SG | 4000~400 | LassoR | Milk | 0.882 | 2.904 | 0.621 | 1.543 |
| SFA | MSC | 4000~400 | PLSR | Milk | 0.671 | 1.748 | 0.676 | 1.769 |
| SFA | DER1 | 4000~400 | PLSR | Milk | 0.679 | 1.886 | 0.665 | 1.737 |
| SFA | DER2 | 4000~400 | PLSR | Milk | 0.693 | 1.818 | 0.677 | 1.771 |
| SFA | SNV | 4000~400 | PLSR | Milk | 0.672 | 1.751 | 0.681 | 1.783 |
| SFA | SG | 4000~400 | PLSR | Milk | 0.684 | 1.785 | 0.641 | 1.679 |
| SFA | MSC | 4000~400 | RFR | Milk | 0.664 | 1.727 | 0.746 | 1.991 |
| SFA | DER1 | 4000~400 | RFR | Milk | 0.713 | 1.869 | 0.730 | 1.934 |
| SFA | DER2 | 4000~400 | RFR | Milk | 0.696 | 1.817 | 0.741 | 1.971 |
| SFA | SNV | 4000~400 | RFR | Milk | 0.666 | 1.732 | 0.714 | 1.872 |
| SFA | SG | 4000~400 | RFR | Milk | 0.704 | 1.840 | 0.732 | 1.937 |
| SFA | MSC | 4000~400 | RidgeR | Milk | 0.709 | 1.851 | 0.702 | 1.832 |
| SFA | DER1 | 4000~400 | RidgeR | Milk | 0.763 | 2.047 | 0.696 | 1.796 |

| | | | | | | | | |
|---|---|---|---|---|---|---|---|---|
| SFA | DER2 | 4000~400 | RidgeR | Milk | 0.804 | 2.217 | 0.692 | 1.797 |
| SFA | SNV | 4000~400 | RidgeR | Milk | 0.714 | 1.865 | 0.715 | 1.878 |
| SFA | SG | 4000~400 | RidgeR | Milk | 0.723 | 1.900 | 0.675 | 1.738 |
| UFA | MSC | 3017~2823/1805~1734 | LassoR | Fat | 0.419 | 1.313 | 0.478 | 1.382 |
| UFA | DER1 | 3017~2823/1805~1734 | LassoR | Fat | 0.412 | 1.305 | 0.388 | 1.276 |
| UFA | DER2 | 3017~2823/1805~1734 | LassoR | Fat | 0.513 | 1.434 | 0.372 | 1.264 |
| UFA | SNV | 3017~2823/1805~1734 | LassoR | Fat | 0.418 | 1.312 | 0.471 | 1.363 |
| UFA | SG | 3017~2823/1805~1734 | LassoR | Fat | 0.368 | 1.259 | 0.440 | 1.323 |
| UFA | MSC | 3017~2823/1805~1734 | RidgeR | Fat | 0.323 | 1.214 | 0.369 | 1.243 |
| UFA | DER1 | 3017~2823/1805~1734 | RidgeR | Fat | 0.374 | 1.262 | 0.381 | 1.262 |
| UFA | DER2 | 3017~2823/1805~1734 | RidgeR | Fat | 0.437 | 1.331 | 0.383 | 1.268 |
| UFA | SNV | 3017~2823/1805~1734 | RidgeR | Fat | 0.323 | 1.215 | 0.378 | 1.242 |
| UFA | SG | 3017~2823/1805~1734 | RidgeR | Fat | 0.264 | 1.167 | 0.292 | 1.179 |
| UFA | MSC | 3017~2823/1805~1734 | RFR | Fat | 0.178 | 1.089 | 0.253 | 1.144 |
| UFA | DER1 | 3017~2823/1805~1734 | RFR | Fat | 0.204 | 1.119 | 0.200 | 1.107 |
| UFA | DER2 | 3017~2823/1805~1734 | RFR | Fat | 0.258 | 1.163 | 0.256 | 1.148 |
| UFA | SNV | 3017~2823/1805~1734 | RFR | Fat | 0.173 | 1.085 | 0.199 | 1.099 |
| UFA | SG | 3017~2823/1805~1734 | RFR | Fat | 0.167 | 1.084 | 0.194 | 1.092 |
| UFA | MSC | 3017~2823/1805~1734 | PLSR | Fat | 0.335 | 1.231 | 0.429 | 1.332 |
| UFA | DER1 | 3017~2823/1805~1734 | PLSR | Fat | 0.289 | 1.192 | 0.396 | 1.294 |
| UFA | DER2 | 3017~2823/1805~1734 | PLSR | Fat | 0.320 | 1.218 | 0.391 | 1.289 |
| UFA | SNV | 3017~2823/1805~1734 | PLSR | Fat | 0.344 | 1.239 | 0.445 | 1.351 |
| UFA | SG | 3017~2823/1805~1734 | PLSR | Fat | 0.302 | 1.205 | 0.463 | 1.373 |
| UFA | MSC | 3017~2823/1805~1734 | LassoR | Milk | 0.602 | 1.589 | 0.776 | 1.998 |
| UFA | DER1 | 3017~2823/1805~1734 | LassoR | Milk | 0.601 | 1.585 | 0.785 | 1.999 |
| UFA | DER2 | 3017~2823/1805~1734 | LassoR | Milk | 0.619 | 1.622 | 0.815 | 2.149 |
| UFA | SNV | 3017~2823/1805~1734 | LassoR | Milk | 0.612 | 1.609 | 0.788 | 2.029 |
| UFA | SG | 3017~2823/1805~1734 | LassoR | Milk | 0.587 | 1.559 | 0.772 | 1.953 |
| UFA | MSC | 3017~2823/1805~1734 | PLSR | Milk | 0.553 | 1.502 | 0.740 | 1.975 |
| UFA | DER1 | 3017~2823/1805~1734 | PLSR | Milk | 0.533 | 1.470 | 0.743 | 1.985 |
| UFA | DER2 | 3017~2823/1805~1734 | PLSR | Milk | 0.527 | 1.461 | 0.771 | 2.101 |
| UFA | SNV | 3017~2823/1805~1734 | PLSR | Milk | 0.557 | 1.508 | 0.752 | 2.022 |
| UFA | SG | 3017~2823/1805~1734 | PLSR | Milk | 0.544 | 1.486 | 0.725 | 1.918 |
| UFA | MSC | 3017~2823/1805~1734 | RFR | Milk | 0.497 | 1.408 | 0.676 | 1.710 |
| UFA | DER1 | 3017~2823/1805~1734 | RFR | Milk | 0.496 | 1.407 | 0.708 | 1.775 |
| UFA | DER2 | 3017~2823/1805~1734 | RFR | Milk | 0.501 | 1.417 | 0.728 | 1.785 |
| UFA | SNV | 3017~2823/1805~1734 | RFR | Milk | 0.501 | 1.411 | 0.638 | 1.611 |
| UFA | SG | 3017~2823/1805~1734 | RFR | Milk | 0.499 | 1.413 | 0.598 | 1.563 |
| UFA | MSC | 3017~2823/1805~1734 | RidgeR | Milk | 0.560 | 1.511 | 0.710 | 1.769 |
| UFA | DER1 | 3017~2823/1805~1734 | RidgeR | Milk | 0.574 | 1.535 | 0.778 | 1.921 |
| UFA | DER2 | 3017~2823/1805~1734 | RidgeR | Milk | 0.589 | 1.561 | 0.809 | 2.050 |
| UFA | SNV | 3017~2823/1805~1734 | RidgeR | Milk | 0.559 | 1.508 | 0.710 | 1.747 |
| UFA | SG | 3017~2823/1805~1734 | RidgeR | Milk | 0.546 | 1.488 | 0.724 | 1.760 |
| UFA | MSC | 4000~400 | RidgeR | Fat | 0.404 | 1.269 | 0.271 | 1.161 |
| UFA | DER1 | 4000~400 | RidgeR | Fat | 0.490 | 1.357 | 0.322 | 1.196 |
| UFA | DER2 | 4000~400 | RidgeR | Fat | 0.554 | 1.414 | 0.340 | 1.206 |
| UFA | SNV | 4000~400 | RidgeR | Fat | 0.416 | 1.290 | 0.274 | 1.158 |
| UFA | SG | 4000~400 | RidgeR | Fat | 0.368 | 1.241 | 0.248 | 1.148 |
| UFA | MSC | 4000~400 | RFR | Fat | 0.209 | 1.123 | 0.190 | 1.103 |
| UFA | DER1 | 4000~400 | RFR | Fat | 0.239 | 1.148 | 0.298 | 1.181 |

| | | | | | | | | |
|---|---|---|---|---|---|---|---|---|
| UFA | DER2 | 4000~400 | RFR | Fat | 0.235 | 1.145 | 0.330 | 1.209 |
| UFA | SNV | 4000~400 | RFR | Fat | 0.200 | 1.116 | 0.182 | 1.092 |
| UFA | SG | 4000~400 | RFR | Fat | 0.197 | 1.114 | 0.182 | 1.100 |
| UFA | MSC | 4000~400 | PLSR | Fat | 0.263 | 1.174 | 0.234 | 1.150 |
| UFA | DER1 | 4000~400 | PLSR | Fat | 0.203 | 1.129 | 0.316 | 1.217 |
| UFA | DER2 | 4000~400 | PLSR | Fat | 0.185 | 1.138 | 0.299 | 1.202 |
| UFA | SNV | 4000~400 | PLSR | Fat | 0.261 | 1.173 | 0.234 | 1.150 |
| UFA | SG | 4000~400 | PLSR | Fat | 0.226 | 1.149 | 0.165 | 1.101 |
| UFA | MSC | 4000~400 | LassoR | Fat | 0.825 | 2.337 | 0.082 | 0.965 |
| UFA | DER1 | 4000~400 | LassoR | Fat | 0.936 | 3.597 | 0.163 | 1.056 |
| UFA | DER2 | 4000~400 | LassoR | Fat | 0.994 | 10.575 | 0.146 | 1.042 |
| UFA | SNV | 4000~400 | LassoR | Fat | 0.834 | 2.403 | 0.072 | 0.953 |
| UFA | SG | 4000~400 | LassoR | Fat | 0.732 | 1.896 | 0.171 | 1.063 |
| UFA | MSC | 4000~400 | LassoR | Milk | 0.864 | 2.683 | 0.595 | 1.543 |
| UFA | DER1 | 4000~400 | LassoR | Milk | 0.974 | 5.910 | 0.534 | 1.417 |
| UFA | DER2 | 4000~400 | LassoR | Milk | 0.994 | 11.922 | 0.620 | 1.605 |
| UFA | SNV | 4000~400 | LassoR | Milk | 0.861 | 2.653 | 0.605 | 1.563 |
| UFA | SG | 4000~400 | LassoR | Milk | 0.805 | 2.247 | 0.632 | 1.606 |
| UFA | MSC | 4000~400 | PLSR | Milk | 0.529 | 1.461 | 0.680 | 1.779 |
| UFA | DER1 | 4000~400 | PLSR | Milk | 0.520 | 1.479 | 0.580 | 1.553 |
| UFA | DER2 | 4000~400 | PLSR | Milk | 0.460 | 1.405 | 0.672 | 1.756 |
| UFA | SNV | 4000~400 | PLSR | Milk | 0.530 | 1.463 | 0.679 | 1.777 |
| UFA | SG | 4000~400 | PLSR | Milk | 0.522 | 1.450 | 0.649 | 1.698 |
| UFA | MSC | 4000~400 | RFR | Milk | 0.512 | 1.433 | 0.662 | 1.657 |
| UFA | DER1 | 4000~400 | RFR | Milk | 0.527 | 1.457 | 0.712 | 1.765 |
| UFA | DER2 | 4000~400 | RFR | Milk | 0.522 | 1.450 | 0.708 | 1.735 |
| UFA | SNV | 4000~400 | RFR | Milk | 0.495 | 1.408 | 0.659 | 1.655 |
| UFA | SG | 4000~400 | RFR | Milk | 0.492 | 1.405 | 0.612 | 1.580 |
| UFA | MSC | 4000~400 | RidgeR | Milk | 0.594 | 1.568 | 0.719 | 1.766 |
| UFA | DER1 | 4000~400 | RidgeR | Milk | 0.660 | 1.703 | 0.745 | 1.825 |
| UFA | DER2 | 4000~400 | RidgeR | Milk | 0.714 | 1.818 | 0.766 | 1.866 |
| UFA | SNV | 4000~400 | RidgeR | Milk | 0.595 | 1.569 | 0.722 | 1.768 |
| UFA | SG | 4000~400 | RidgeR | Milk | 0.566 | 1.518 | 0.721 | 1.750 |
